# Body-mass index and risk of obesity-related complex multimorbidity: an observational multicohort study

**DOI:** 10.1016/S2213-8587(22)00033-X

**Published:** 2022-04

**Authors:** Mika Kivimäki, Timo Strandberg, Jaana Pentti, Solja T Nyberg, Philipp Frank, Markus Jokela, Jenni Ervasti, Sakari B Suominen, Jussi Vahtera, Pyry N Sipilä, Joni V Lindbohm, Jane E Ferrie

**Affiliations:** a Department of Epidemiology and Public Health, University College London, London, UK; b Clinicum, Faculty of Medicine, University of Helsinki, Helsinki, Finland; c Department of Psychology and Logopedics, University of Helsinki, Helsinki, Finland; d Finnish Institute of Occupational Health, Helsinki, Finland; e Department of Medicine, University of Helsinki and Helsinki University Hospital, Helsinki, Finland; f Center for Life Course Health Research, University of Oulu, Oulu, Finland; g Department of Public Health, University of Turku, Turku, Finland; h Centre for Population Health Research, University of Turku, Turku, Finland; i Turku University Hospital, Turku, Finland; j School of Health Science, University of Skövde, Skövde, Sweden; k Bristol Medical School, University of Bristol, Bristol, UK

## Abstract

**Background:**

The accumulation of disparate diseases in complex multimorbidity makes prevention difficult if each disease is targeted separately. We aimed to examine obesity as a shared risk factor for common diseases, determine associations between obesity-related diseases, and examine the role of obesity in the development of complex multimorbidity (four or more comorbid diseases).

**Methods:**

We did an observational study and used pooled prospective data from two Finnish cohort studies (the Health and Social Support Study and the Finnish Public Sector Study) comprising 114 657 adults aged 16–78 years at study entry (1998–2013). A cohort of 499 357 adults (aged 38–73 years at study entry; 2006–10) from the UK Biobank provided replication in an independent population. BMI and clinical characteristics were assessed at baseline. BMIs were categorised as obesity (≥30·0 kg/m^2^), overweight (25·0–29·9 kg/m^2^), healthy weight (18·5–24·9 kg/m^2^), and underweight (<18·5 kg/m^2^). Via linkage to national health records, participants were followed-up for death and diseases diagnosed according to the International Classification of Diseases 10th Revision (ICD-10). Hazard ratios (HRs) with 95% CIs and population attributable fractions (PAFs) for associations between BMI and multimorbidity were calculated.

**Findings:**

Mean follow-up duration was 12·1 years (SD 3·8) in the Finnish cohorts and 11·8 years (1·7) in the UK Biobank cohort. Obesity was associated with 21 non-overlapping cardiometabolic, digestive, respiratory, neurological, musculoskeletal, and infectious diseases after Bonferroni multiple testing adjustment and ignoring HRs of less than 1·50. Compared with healthy weight, the confounder-adjusted HR for obesity was 2·83 (95% CI 2·74–2·93; PAF 19·9% [95% CI 19·3–20·5]) for developing at least one obesity-related disease, 5·17 (4·84–5·53; 34·4% [33·2–35·5]) for two diseases, and 12·39 (9·26–16·58; 55·2% [50·9–57·5]) for complex multimorbidity. The proportion of participants of healthy weight with complex multimorbidity by age 75 years was observed by age 55 years in participants with obesity, and degree of obesity was associated with complex multimorbidity in a dose–response relationship. Compared with obesity, the association between overweight and complex multimorbidity was more modest (HR 2·67, 95% CI 1·94–3·68; PAF 13·3% [95% CI 9·6–16·3]). The same pattern of results was observed in the UK Biobank cohort.

**Interpretation:**

Obesity is associated with diverse, increasing disease burdens, and might represent an important target for multimorbidity prevention that avoids the complexities of multitarget preventive regimens.

**Funding:**

Wellcome Trust, Medical Research Council, National Institute on Aging.

## Introduction

Obesity prevalence and obesity-related disease burdens are increasing worldwide.^1^, ^2^ WHO has estimated that globally there are more than 650 million people with obesity (BMI ≥30 kg/m^2^).^3^ According to large observational studies, obesity is associated with a reduction in disease-free life of 3–8 years^4^ and with an approximately 1·3 times excess risk of premature death compared with people with healthy weight.^5^, ^6^ In addition, obesity predisposes people to a large array of clinical conditions, including type 2 diabetes, cardiovascular disease, chronic kidney disease, site-specific cancers, musculoskeletal disorders, and infections.^7^, ^8^ However, it is unclear whether these diseases are distributed across all people with obesity, or cluster in smaller groups of individuals with obesity-related multimorbidity.

There is evidence to suggest that obesity leads to disease clustering, frailty, and poor health-related quality of life.^9^, ^10^, ^11^, ^12^, ^13^, ^14^, ^15^ In the IPD-Work multicohort study of 120 000 adults, people with mild obesity (BMI 30·0–34·9 kg/m^2^) had more than four times increased risk of cardiometabolic multimorbidity (two or all three of myocardial infarction, stroke, and type 2 diabetes) compared with individuals with healthy weight.^15^ A study of more than 11 000 adults found that participants who had overweight or obesity were twice as likely to have comorbidities, including hypertension, dyslipidaemia, unspecified diabetes, and osteoarthritis.^16^ A large cohort study of patients aged 45–64 years in the USA found that the most prevalent disease clusters included hypertension and dorsalgia in people with healthy weight; together with joint disorders, dyslipidaemia, type 2 diabetes, sleep disorders, and chronic kidney disease in people who had overweight or obesity.^17^ However, few studies have characterised a wide range of obesity-related conditions that result in complex multimorbidity (four or more comorbid diseases).^18^

**Research in context**

**Evidence before this study**

Although obesity is a risk factor for many diseases, little is known about the co-occurrence of these conditions. We searched PubMed from database inception to June 9, 2021, with no language restrictions, using the terms “obesity” AND “outcome-wide” OR “phenome-wide” OR “multimorbidity”, and identified more than 400 studies. The search results suggested that obesity is linked to more than 250 genetic variants and a wide array of clinical conditions, including diseases of the circulatory, endocrine, digestive, neurological, dermatological, musculoskeletal, respiratory, and genitourinary systems, cancer, injuries, and poisonings. Despite reports of obesity-related disease clustering, few studies have examined the role of obesity in the development of complex multimorbidity, defined as four or more comorbid diseases, and, to our knowledge, no studies have yet defined multimorbidity using a wide range of obesity-related conditions.

**Added value of this study**

To facilitate a more comprehensive evaluation of obesity-related complex multimorbidity, we analysed individual-level data from two Finnish cohort studies and the UK Biobank, examined obesity as a risk factor for incident cases of 78 predefined diseases, determined associations between obesity-related diseases, and characterised resulting patterns of obesity-related complex multimorbidity. Ignoring lesser effects (hazard ratio <1·50) and controlling for multiple testing, obesity was associated with 21 major diseases, but there was only a 1·3 times excess risk of death among participants with obesity in the Finnish cohorts compared with those with healthy weight. These diseases were interconnected, leading to disease clustering within individuals and a 12·4 times excess risk of complex multimorbidity among participants with obesity compared with participants with healthy weight, and a population attributable fraction of 55·2%. Within the group of participants with obesity, degree of obesity was associated with complex multimorbidity in a dose–response relationship. This obesity-related complex multimorbidity was highly variable, as the first four diseases formed 140 different combinations. The same pattern of findings was replicated in the UK Biobank cohort.

**Implications of all the available evidence**

Due to the accumulation of disparate diseases, complex multimorbidity presents a difficult prevention target if each disease is targeted separately. Our findings show that obesity exposes people to gradually worsening burdens of heterogeneous multimorbidities, including cardiometabolic, digestive, respiratory, neurological, musculoskeletal, infectious, and malignant diseases. This association suggests that obesity is an amenable target for complex multimorbidity prevention that avoids the problems of multitarget regimens. Creating healthy living conditions with less risk factors that predispose to obesity presents a parsimonious approach to reduce multimorbidity at the population level, while obesity treatments, such as lifestyle interventions, pharmacotherapy, and bariatric surgery could prevent multimorbidity among those who receive them.

We aimed to examine the risk of common health conditions among people with obesity, determine associations in the emergence of obesity-related conditions, and characterise the role of obesity in the development of complex multimorbidity at older ages.

## Methods

### Study design and participants

We did an observational study and used pooled prospective data from two Finnish cohort studies: the Health and Social Support Study (HeSSup)^15^ and the Finnish Public Sector Study (FPS).^16^ In HeSSup, 64 797 men and women aged 20–54 years and living in Finland were sent a survey between June 7, 1998, and May 23, 2000; or Jan 7 and Aug 12, 2003. Responders were linked electronically to national hospitalisation and mortality registers until Dec 31, 2015. In FPS, 113 578 men and women aged 16–78 years and in employment were sent a survey between March 1, 2000, and June 30, 2002; March 1, 2004, and June 30, 2005; March 1, 2008, and Nov 30, 2009; or Dec 1, 2011, and Nov 30, 2013. Study participants were linked to electronic health records until Dec 31, 2018.

To examine the robustness and generalisability of our findings, we repeated our main analyses in an independent cohort from the UK Biobank. This cohort included 502 665 adults aged 38–73 years, identified from National Health Service (NHS) records in the UK, who participated in baseline examination between March 13, 2006, and Oct 1, 2010, and who were followed-up for hospital admissions and deaths until March 31, 2021.

Ethical approval was obtained from local committees on the ethics of human research. Analyses of the UK Biobank cohort were conducted under generic approval from the NHS National Research Ethics Service (2CFFAA23-CEC4-4AF0-9133-405139170B01). Participants provided electronic informed consent for baseline assessments and register linkage. Further details on methods, including cohort descriptions, are provided in the appendix (pp 3–17).

### Procedures

Weight and height at baseline were self-reported in HeSSup and FPS and were measured as part of the examination in the UK Biobank. We calculated BMI using the formula: weight (kg) divided by height (m) squared. BMIs were categorised as obesity (≥30·0 kg/m^2^), overweight (25·0–29·9 kg/m^2^), healthy weight (18·5–24·9 kg/m^2^), and underweight (<18·5 kg/m^2^). Obesity was further divided into class 1 (BMI 30·0–34·9 kg/m^2^), class 2 (35·0–39·9 kg/m^2^), and class 3 (≥40 kg/m^2^).

In addition to age, sex, and cohort, baseline characteristics included level of education and neighbourhood deprivation—factors that have been shown to correlate with death and a wide range of diseases and predict the development of obesity. In social experiments, a reduction in socioeconomic deprivation has been associated with subsequent reduction in obesity.^19^ Further covariates included lifestyle factors: current smoking (yes *vs* no), heavy drinking (>21 units of alcohol per week for men and >14 for women *vs* 0–21 units of alcohol for men and 0–14 for women), and physical activity (low [none or little moderate or vigorous leisure-time physical activity] *vs* high [some or much moderate or vigorous leisure-time physical activity]).

Participants from HeSSup and FPS were linked by their unique identification number to national hospital discharge (recorded by the Finnish Institute for Health and Welfare) and mortality (recorded by Statistics Finland) registries. These electronic health records included cause and date of hospitalisation or mortality, or both, from Jan 1, 1996, to Dec 31, 2018. Additional information on site-specific cancers, all diabetes, cardiovascular diseases (including hypertension), psychotic disorder, dementia, Parkinson's disease, multiple sclerosis, epilepsy, asthma, chronic obstructive pulmonary disease, inflammatory bowel disease, rheumatoid arthritis, gout, and renal failure was available via record linkage to the Drug Reimbursement Register of the Social Insurance Institution of Finland (from Jan 1, 1980, to Dec 30, 2018). UK Biobank participants were linked to the UK NHS Hospital Episode Statistics database for hospital admissions and the NHS Central Registry for mortality, from March 18, 1995, to March 31, 2021.

In all three cohort studies, diseases were coded according to WHO's International Classification of Diseases 10th Revision (ICD-10), capturing a total of 1204 three-character diagnostic codes. We excluded hospitalisations due to obesity and focused on a predefined list of 78 common ICD-10 disease chapters and diagnostic groups constructed for outcome-wide studies by investigators who were masked to exposure data (including BMI).^20^

### Statistical analysis

Participants with morbidity and mortality follow-up and no missing data on age, sex, or BMI were included in our analyses. Missing data were treated as a separate category for other covariates. After we assessed the proportional hazards assumption, we examined associations between obesity and the 78 health outcomes in separate models using Cox proportional hazards regression in the Finnish cohorts. In these analyses, follow-up started from BMI assessment (baseline, at study entry) and continued until onset of the diseases of interest, loss to follow-up, death, or end of follow-up, whichever came first. Hazard ratios (HRs) and 95% CIs for obesity, with healthy weight as the reference, were adjusted for age, sex, education, neighbourhood deprivation, and cohort (the basic model). To focus on diseases that are more common in participants with obesity, and differences in disease risk that were likely to be meaningful for public health and health care, we considered only obesity-disease associations that yielded HRs greater than or equal to 1·50,^21^ and were significant at a Bonferroni corrected α level, with a p value of less than 6·3 × 10^–4^ (78 tests). Sex differences in associations between obesity and incident disease were examined by including a sex–BMI interaction term in a Cox model in addition to their main effects and adjustments as in the basic model.

Further analyses focused on the obesity-related diseases after excluding overlapping diagnoses. To examine obesity in relation to the co-occurrence of obesity-related diseases, we constructed four disease outcomes: onset of the first, second, third, and fourth obesity-related disease. The second outcome (two obesity-related diseases) refers to simple multimorbidity and the last outcome (four or more obesity-related diseases) refers to complex multimorbidity.^18^, ^22^ The number of new-onset obesity-related diseases accrued by the end of follow-up or death determined the allocation of participants to each of the four health outcomes. To assess the dose–response relationship within the obesity category, we stratified obesity into classes 1, 2, and 3. In addition to HRs and 95% CIs, we calculated population attributable fractions (PAFs) to evaluate the potential reduction in obesity-related multimorbidity if exposure to obesity was removed.

To further increase understanding of obesity-related multimorbidity, we examined temporal sequences in the emergence of obesity-related diseases by testing prospective associations between all obesity-related disease pairs in individuals with obesity. To describe patterns of complex multimorbidity, we computed the frequency of each obesity-related disease in participants with obesity who developed four or more obesity-related diseases during follow-up.

To examine reproducibility of the findings from the Finnish cohorts, associations between obesity, diseases, and obesity-related multimorbidity were tested in a replication analysis in the UK Biobank cohort. Additional sensitivity analyses tested whether the main findings were reproducible using alternative reference groups, definitions of multimorbidity, and statistical methods.

All analyses were performed using SAS version 9.4.

### Role of the funding source

The funders of the study had no role in study design, data collection, data analysis, data interpretation, or writing of the report.

## Results

**Figure 1:**
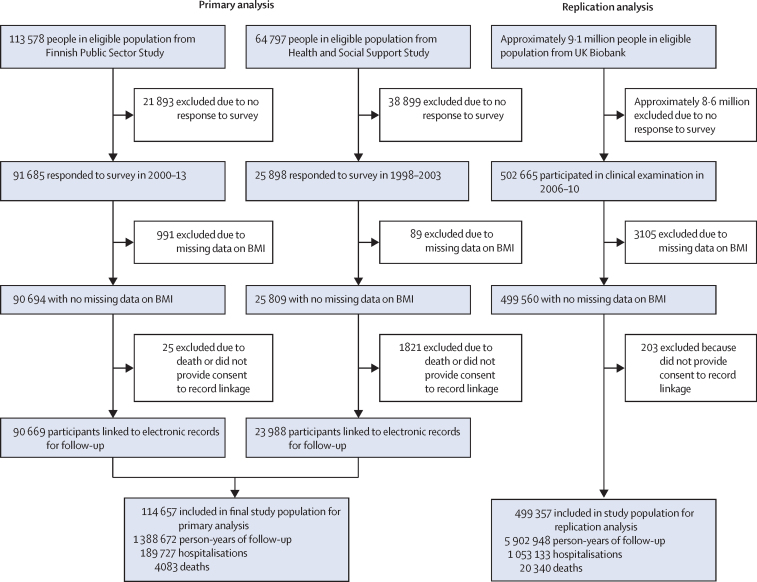
Study profile

Of the 178 375 adults in the eligible population from the two Finnish cohorts, 117 583 (65·9%) participated in the baseline survey, 116 503 (65·3%) had data on height and weight, and 114 657 (64·3%) were successfully linked to national health registers and were included in the sample for primary analysis (figure 1). In the primary analysis population, the mean age at BMI assessment (baseline) was 42·6 years (SD 11·0), 86 632 (75·6%) were women and 28 025 (24·4%) were men, and 57 142 (49·8%) had completed tertiary education or higher (table 1). For comparison, in the eligible population, mean age (40·5 years [SD 10·8]), the proportion of women (120 170 [67·4%]), and the proportion who had completed tertiary education or higher (85 442 [47·9%]) were lower than in the primary analysis population.

**Table 1:** Baseline characteristics

	**Finnish Public Sector Study cohort (n=90 669)**	**Health and Social Support Study cohort (n=23 988)**	**UK Biobank cohort (n=499 357)**
**Year of study entry**			
1998	0	22 901 (95·5%)	0
2000–03	42 633 (47·0%)	1087 (4·5%)	0
2004–05	20 298 (22·4%)	0	0
2006–07	0	0	53 251 (10·7%)
2008–09	17 284 (19·1%)	0	358 790 (71·9%)
2010	0	0	87 316 (17·5%)
2012–13	10 454 (11·5%)	0	0
**Age, years**			
Mean (SD)	44·1 (10·3)	37·0 (11·5)	57·0 (8·1)
<40	31 232 (34·5%)	12 162 (50·7%)	3 (<0·1%)
40–49	28 893 (31·9%)	5694 (23·7%)	109 977 (22·0%)
50–59	25 490 (28·1%)	6132 (25·6%)	162 990 (32·6%)
≥60	5054 (5·6%)	0	226 387 (45·3%)
**Sex**			
Male	18 194 (20·1%)	9831 (41·0%)	227 461 (45·6%)
Female	72 475 (79·9%)	14 157 (59·0%)	271 896 (54·5%)
**Ethnicity**			
White	0	0	470 526 (94·2%)
Non-White	0	0	26 475 (5·3%)
Missing	90 669 (100%)	23 988 (100%)	2356 (0·5%)
**Level of education**			
Primary	7685 (8·5%)	7121 (29·7%)	84 560 (16·9%)
Secondary	29 830 (32·9%)	12 630 (52·7%)	244 799 (49·0%)
Tertiary or higher	53 153 (58·6%)	3989 (16·6%)	160 383 (32·1%)
Missing	1 (<0·1%)	248 (1·0%)	9615 (1·9%)
**Neighbourhood deprivation**			
Low	34 706 (38·3%)	6351 (26·5%)	125 656 (25·2%)
Medium	23 995 (26·5%)	5831 (24·3%)	248 966 (49·9%)
High	26 319 (29·0%)	8554 (35·7%)	124 127 (24·9%)
Missing	5649 (6·2%)	3252 (13·6%)	608 (0·1%)
**BMI, kg/m^2^**			
Mean (SD)	25·5 (4·6)	24·8 (4·5)	27·4 (4·8)
<18·5 (underweight)	1084 (1·2%)	555 (2·3%)	2626 (0·5%)
18·5–24·9 (healthy weight)	48 105 (53·1%)	14 014 (58·4%)	162 395 (32·5%)
25·0–29·9 (overweight)	2 574 (26·0%)	6086 (25·4%)	212 098 (42·5%)
≥30·0 (obesity)	17 906 (19·8%)	3333 (13·9%)	122 238 (24·5%)
**Obesity severity by BMI, kg/m^2^**			
30·0–34·9 (class 1)	14 792 (16·3%)	2746 (11·5%)	87 543 (17·5%)
35·0–39·9 (class 2)	2377 (2·6%)	446 (1·9%)	24 992 (5·0%)
≥40·0 (class 3)	737 (0·8%)	141 (0·6%)	9703 (1·9%)
**Smoking at baseline**			
No	72 794 (80·3%)	17 806 (74·2%)	445 929 (89·3%)
Yes	14 780 (16·3%)	6082 (25·4%)	52 508 (10·5%)
Missing	3095 (3·4%)	100 (0·4%)	920 (0·2%)
**Heavy drinking**			
No	82 197 (90·7%)	21 819 (91·0%)	396 942 (79·5%)
Yes	7113 (7·9%)	2134 (8·9%)	101 327 (20·3%)
Missing	1359 (1·5%)	35 (0·2%)	1088 (0·2%)
**Physical inactivity**			
No	58 315 (64·3%)	16 438 (68·5%)	324 926 (65·1%)
Yes	30 981 (34·2%)	7370 (30·7%)	75 461 (15·1%)
Missing	1373 (1·5%)	180 (0·8%)	98 970 (19·8%)

Data are n (%) unless otherwise stated.

**Figure 2:**
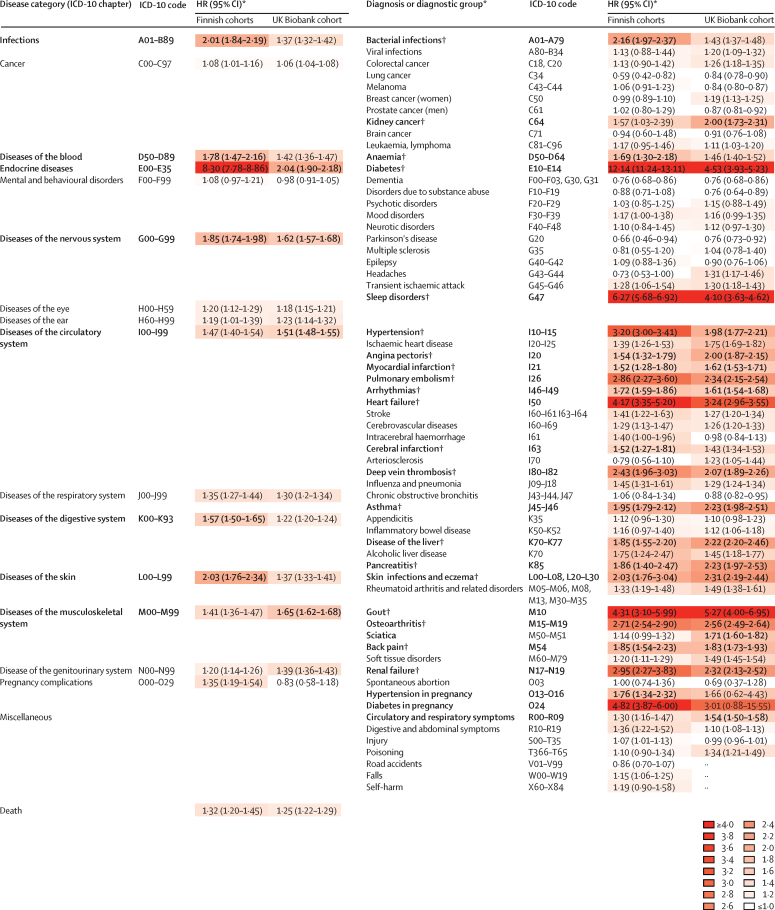
Associations between obesity and incidence of 78 diseases and death ICD-10=International Classification of Diseases 10th Revision. HR=hazard ratio. *HR for obesity (BMI ≥30·0 kg/m^2^) versus healthy weight (18·5–24·9 kg/m^2^), adjusted for age, sex, cohort (Finnish cohorts), education, and neighbourhood deprivation (basic model); HRs greater than or equal to 1·50 and significant at p<0·0006 (Bonferroni correction for multiple testing) are shown in bold. †Non-overlapping disease diagnoses included in analysis of obesity-related multimorbidity.

In the Finnish cohorts, 62 119 (54·2%) of 114 657 participants had healthy weight, 29 660 (25·9%) were overweight, 21 239 (18·5%) had obesity, and 1639 (1·4%) were underweight. During 1 388 672 person-years at risk (mean follow-up 12·1 years [SD 3·8]), 189 727 incident diseases and 4083 deaths were recorded. Obesity was associated with a 1·32 times (95% CI 1·20–1·45) increased risk of death (the corresponding HR for class 3 obesity was 1·90 [95% CI 1·46–2·48]). Ignoring lesser effects (adjusted HR <1·50) and associations with a p value of 6·3 × 10^–4^ or greater, overweight was associated with only one disease (hypertension in pregnancy in women) and underweight with none (appendix pp 18–20). By contrast, obesity was associated with 28 of the 78 diseases studied (figure 2).

Further analysis omitted six broad obesity-related ICD-10 chapters where specific disease outcomes within these were more strongly related to obesity, to focus on non-overlapping obesity-related disease diagnoses. Pregnancy-related hypertension and gestational diabetes were also omitted as their association with obesity was weaker than for hypertension and all adult-onset diabetes (except gestational diabetes). Due to small numbers, kidney cancer reached an HR greater than or equal to 1·50 but did not reach Bonferroni significance in the Finnish cohorts (p=0·036). As kidney cancer reached an HR greater than or equal to 1·50 and significance in the UK Biobank cohort, it was included in our multimorbidity analyses. Thus, the multimorbidity analyses were based on 21 non-overlapping obesity-related diseases, including endocrine (all adult-onset diabetes), cardiovascular (hypertension, angina, myocardial infarction, heart failure, arrhythmia, cerebral infarction, deep vein thrombosis, pulmonary embolism), digestive (pancreatitis, liver disease), infectious (bacterial infections), musculoskeletal (gout, osteoarthritis, back pain), respiratory (asthma), malignant (kidney cancer), skin (skin infections and eczema), blood (anaemia), genitourinary (renal failure), and nervous system (sleep disorders—mostly sleep apnoea) diseases.

**Figure 3:**
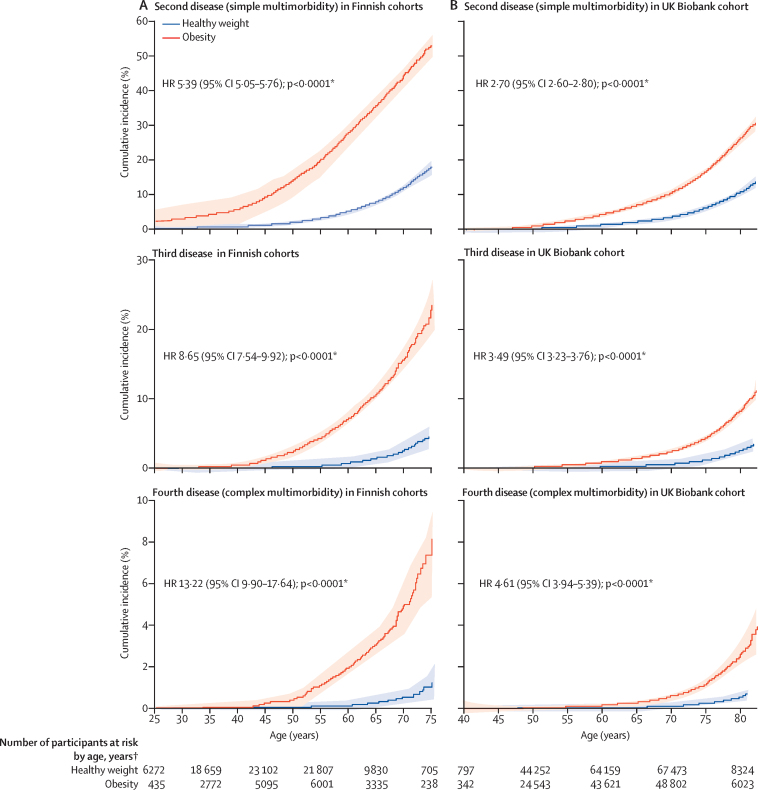
Cumulative incidence of obesity-related simple multimorbidity and complex multimorbidity by age with obesity versus healthy weight (A) Finnish cohorts. (B) UK Biobank cohort. HR=hazard ratio. *Hazard ratio (95% CI) adjusted for age, sex, ethnicity (UK Biobank), and cohort (Finnish cohorts). †Maximum number of participants included in follow-up.

Participants with obesity were at higher risk of developing simple (two diseases) and complex (four or more diseases) multimorbidity compared with participants with healthy weight (figure 3). Separation between the groups in the hazard curves for these outcomes started at age 30 years for simple multimorbidity and at 45 years for complex multimorbidity, and continued across the full age range. The proportion of participants with simple or complex multimorbidity by age 75·0 years among those of healthy weight was reached by age 53·6 years for simple multimorbidity and 55·0 years for complex multimorbidity in those with obesity (figure 3A). By age 75 years in participants with obesity, the estimated incidence of simple multimorbidity was 53·3% (95% CI 50·1–56·3) and of complex multimorbidity was 8·3% (6·0–10·4). Among participants with healthy weight, these incidences were 8·3% (6·0–10·4) and 1·0% (0·6–1·4), respectively (figure 3A).

The relative risks of developing the first, second, third, and fourth obesity-related disease by BMI category, and the corresponding PAFs, are shown in table 2. The HR for obesity versus healthy weight was 2·83 (95% CI 2·74–2·93; PAF 19·9% [95% CI 19·3–20·5]) for developing at least one obesity-related disease, 5·17 (4·84–5·53; 34·4% [33·2–35·5]) for developing two diseases (simple multimorbidity), and 12·39 (9·26–16·58; 55·2% [50·9–57·5]) for four diseases (complex multimorbidity), whereas the HR for overweight versus healthy weight for complex multimorbidity was more modest (2·67, 1·94–3·68; 13·3% [9·6–16·3]). Degree of obesity was associated with complex multimorbidity in a dose–response relationship; the association was strongest in participants with class 3 obesity. Additional adjustments for lifestyle factors had little effect on the estimates, and subgroup and sensitivity analyses suggested our results were robust (appendix pp 21–24). However, obesity before age 50 years was more strongly related to incident complex multimorbidity (HR 22·11, 95% CI 13·68–35·74) than obesity at older ages (7·90, 5·48–11·41; p=0·0091). A similar difference between age groups was also observed for simple multimorbidity (appendix p 24).

**Table 2:** Association of BMI category with incident obesity-related disease and multimorbidity

	**First disease**			**Second disease**			**Third disease**			**Fourth disease**		
	Incident cases	HR (95% CI)*	PAF (95% CI)	Incident cases	HR (95% CI)*	PAF (95% CI)	Incident cases	HR (95% CI)*	PAF (95% CI)	Incident cases	HR (95% CI)*	PAF (95% CI)
**Finnish cohorts**												
Underweight (n=1476)	161	0·80 (0·69- to 0·94)	−0·2% (−0·4 to −0·1)	29	0·95 (0·66 to 1·37)	0% (−0·3 to 0·3)	8	..	..	1	..	..
Healthy weight (n=55 857)	9373	1 (ref)	0% (ref)	1676	1 (ref)	0% (ref)	309	1 (ref)	0% (ref)	62	1 (ref)	0% (ref)
Overweight (n=24 495)	6813	1·53 (1·49 to 1·58)	9·6% (8·9 to 10·2)	1764	1·89 (1·77 to 2·03)	12·1% (10·9 to 13·3)	431	2·30 (1·98 to 2·66)	13·4% (11·3 to 15·4)	104	2·67 (1·94 to 3·68)	13·3% (9·6 to 16·3)
Obesity (n=14 867)	5672	2·83 (2·74 to 2·93)	19·9% (19·3 to 20·5)	2058	5·17 (4·84 to 5·53)	34·4% (33·2 to 35·5)	645	8·18 (7·12 to 9·39)	45·2% (43·1 to 47·0)	195	12·39 (9·26 to 16·58)	55·2% (50·9 to 57·5)
Class 1 obesity (n=12 496)	4519	2·63 (2·53 to 2·72)	14·8% (14·3 to 15·3)	1565	4·61 (4·30 to 4·95)	24·9% (24·1 to 25·7)	454	6·77 (5·85 to 7·85)	30·4% (29·0 to 31·5)	127	9·56 (7·02 to 13·02)	34·9% (32·2 to 35·9)
Class 2 obesity (n=1839)	875	3·80 (3·54 to 4·07)	3·8% (3·5 to 4·0)	358	7·39 (6·59 to 8·29)	6·5% (6·0 to 7·0)	136	13·63 (11·12 to 16·71)	9·8% (8·9 to 10·6)	53	25·55 (17·63 to 37·04)	14·7% (13·1 to 15·9)
Class 3 obesity (n=532)	278	4·99 (4·43 to 5·62)	1·5% (1·4 to 1·7)	135	12·43 (10·42 to 14·82)	3·4% (2·9 to 3·8)	55	25·22 (18·91 to 33·64)	5·4% (4·6 to 6·4)	15	33·56 (19·04 to 59·13)	5·7% (4·1 to 7·4)
**UK Biobank cohort**												
Underweight (n=2387)	423	1·09 (0·99 to 1·20)	<0·1% (0·0 to 0·1)	74	1·09 (0·87 to 1·38)	0% (−0·1 to 0·1)	20	1·46 (0·94 to 2·28)	0·2% (0·0 to 0·4)	5	..	..
Healthy weight (n=150 423)	25787	1 (ref)	0% (ref)	4620	1 (ref)	0% (ref)	949	1 (ref)	0% (ref)	203	1 (ref)	0% (ref)
Overweight (n=188 761)	43 734	1·28 (1·26 to 1·30)	8·9% (8·4 to 9·4)	9149	1·36 (1·31 to 1·41)	10·3% (9·2 to 11·4)	2032	1·40 (1·30 to 1·51)	10·3% (8·1 to 12·4)	482	1·49 (1·27 to 1·76)	11·1% (6·9 to 14·9)
Obesity (n=100 510)	31 931	1·88 (1·85 to 1·91)	15·2% (14·8 to 15·5)	8638	2·48 (2·39 to 2·57)	22·6% (21·8 to 23·3)	2390	3·12 (2·89 to 3·36)	29·1% (27·6 to 30·5)	691	3·99 (3·41 to 4·68)	35·9% (33·0 to 38·4)
Class 1 obesity (n=73 494)	21 912	1·71 (1·68 to 1·74)	8·9% (8·6 to 9·2)	5569	2·12 (2·04 to 2·20)	12·4% (11·9 to 12·9)	1433	2·47 (2·27 to 2·68)	14·6% (13·6 to 15·6)	388	2·95 (2·49 to 3·51)	16·9% (14·9 to 18·5)
Class 2 obesity (n=19 767)	7048	2·24 (2·18 to 2·30)	4·2% (4·0 to 4·3)	2051	3·15 (2·99 to 3·32)	6·4% (6·1 to 6·7)	623	4·37 (3·95 to 4·84)	9·0% (8·5 to 9·6)	198	6·18 (5·07 to 7·53)	12·0% (11·0 to 13·0)
Class 3 obesity (n=7249)	2971	2·90 (2·79 to 3·01)	2·4% (2·3 to 2·5)	1018	4·95 (4·62 to 5·30)	4·3% (4·1 to 4·5)	334	7·53 (6·64 to 8·54)	6·4% (5·9 to 6·9)	105	10·64 (8·39 to 13·50)	8·2% (7·3 to 9·1)

Data are n unless otherwise stated. HR=hazard ratio. PAF=population attributable fraction.

* Adjusted for age, sex, ethnicity (UK Biobank), cohort (Finnish cohorts), education, and neighbourhood deprivation.

The 21 obesity-related diseases were highly interconnected, such that the presence of one disease increased the risk of developing another disease, and many associations were bidirectional (appendix pp 25–30). Among the first four diseases in participants with obesity and complex multimorbidity, there were 140 different disease combinations, each of low prevalence (≤12 [6·2%)] of 195 participants; appendix p 31). Four (2·9%) of 140 disease combinations included diseases from one ICD-10 chapter only, 23 (16·4%) from two chapters, and 68 (48·6%) from three or four chapters. The obesity-related diseases that occurred most frequently in participants with obesity and complex multimorbidity were all adult-onset diabetes (147 [75·4%] of 195 participants), hypertension (140 [71·8%]), sleep disorders (83 [42·6%]), osteoarthritis (82 [42·1%]), arrhythmias (67 [34·4%]), bacterial infections (61 [31·3%]), and asthma (43 [22·1%]; appendix p 34).

The replication UK Biobank cohort included 499 357 participants, among whom 1 053 133 hospital admissions with incident diseases and 20 340 deaths were recorded during 5 902 948 person-years at risk (mean follow-up 11·8 years [SD 1·7]). Participants in the UK Biobank cohort were older, more equally distributed by sex, had a lower level of education, and had a higher prevalence of obesity than participants in the Finnish cohorts (table 1, appendix p 36). Because follow-up in UK Biobank participants started at age 38 years or older (compared with age 17 years in the Finnish cohorts), incident obesity-related disease cases in early adulthood were not captured in the replication cohort.

The pattern of results in the UK Biobank cohort replicated those observed in the primary analysis, although with smaller effect estimates. Obesity was associated with all 21 obesity-related diseases identified in the Finnish cohorts and there was a moderate association with mortality (HRs 1·43–5·27, p<6·3 × 10^–4^; table 2, appendix p 37). Obesity was similarly associated with incidence of simple and complex multimorbidity, and dose–response relationships that increased in strength with increasing severity of obesity were replicated (figure 3, table 2). After adjustment for age, sex, level of education, and neighbourhood deprivation, the HR (*vs* healthy weight) for developing complex multimorbidity was 2·95 (95% CI 2·49–3·51) for obesity class 1, 6·18 (5·07–7·53) for class 2, and 10·64 (8·39–13·50) for class 3 (table 2). Adjustment for lifestyle factors did not substantially alter these estimates (appendix pp 38–39).

## Discussion

Associations between obesity and a range of diseases are well recognised, but three aspects of our results are novel, or better characterised than in previous studies. First, simultaneous assessment of 78 disease outcomes facilitates a comprehensive understanding of the health effects of obesity. Obesity was robustly (HR ≥1·50, p<0·0006) associated with 21 non-overlapping diseases across multiple organ systems. These diseases were interconnected, such that each obesity-related disease predicted or was predicted by one or more other obesity-related diseases. Second, this interconnectedness accelerated the development of obesity-related multimorbidity. In the Finnish cohorts, the proportion of people with complex multimorbidity by age 75 years among those of healthy weight had already been reached 20 years earlier, at age 55 years, in those with obesity. Compared with participants with healthy weight, people with obesity were at a five times increased risk of simple multimorbidity and more than 12 times increased risk of complex multimorbidity. The risk of complex multimorbidity was greater in participants who had obesity at younger than 50 years than in those who had obesity at older ages. Analyses that stratified obesity into classes 1, 2, and 3 showed a dose–response pattern; the greater the degree of obesity, the greater the relative risk of complex multimorbidity. Third, overall patterns of complex obesity-related multimorbidity were highly variable, including 140 different combinations of the 21 cardiometabolic, digestive, respiratory, neurological, musculoskeletal, infectious, and malignant diseases among the first four obesity-related conditions. Despite the high morbidity burden, obesity was only moderately associated with mortality, suggesting that obesity decreases disease-free survival more than overall survival. These three findings were confirmed in an independent cohort of older participants from the UK Biobank.

Multimorbidity, defined as the presence of at least two co-occurring diseases, is common at ages older than 65 years.^23^ Unlike most previous studies, we focused also on complex multimorbidity (four or more co-occurring diseases), a health outcome that is associated with poor health-related quality of life and increased disability and frailty.^11^, ^24^, ^25^, ^26^ Our findings are supported by several strands of evidence. We identified a broad range of obesity-related conditions in line with genetic studies that have linked obesity-associated genes to many of the same diseases, suggesting that these associations are causal (appendix pp 40–41).^27^ In our analysis, adult-onset diabetes and hypertension were the most common obesity-related conditions at older ages, both as single diseases and as part of complex multimorbidity. This finding is in agreement with pooled data from a WHO study that showed the combination of obesity, type 2 diabetes, and hypertension to be one of the most common multimorbidities across countries.^14^ Furthermore, the excess risk of all-cause mortality in participants with obesity in our study (HR 1·32 overall and 1·90 for class 3 obesity) is in agreement with meta-analyses of up to 239 cohort studies.^5^, ^6^, ^28^

The greater than 12 times increased risk of complex multimorbidity and the five times increased risk of simple multimorbidity associated with obesity in the Finnish cohorts and among those with class 3 obesity in the UK Biobank cohort are similar to effect estimates for multimorbidity from obesity-related diseases in other studies.^10^, ^14^, ^15^, ^16^, ^17^ The IPD-Work multicohort study, which defined multimorbidity as at least two of myocardial infarction, stroke, and type 2 diabetes, found a four times increased risk of multimorbidity in participants with class 1 obesity and an almost 15 times increased risk in the those with class 2 and class 3 obesity compared with participants with healthy weight.^15^ Other studies that have used multimorbidity definitions including diseases related and unrelated to obesity have observed lower effect estimates. In a Korean study of 12 000 adults, participants who had overweight or obesity were twice as likely to have simple multimorbidity (two or more of 30 diseases) than participants with healthy weight.^16^ In an Australian study of 4865 women, participants with obesity were about three times as likely to have an “increasing multimorbidity trajectory” (based on changes in 18 self-reported conditions).^10^ In a register-based study of more than 150 000 patients with one or more diseases, the prevalence ratio for multimorbidity (two or more of 82 diseases) between participants with obesity and those with healthy weight was 1·3.^17^

Our results support a life course model of disease accumulation, in which excess risk of simple multimorbidity emerges in young adulthood and is followed by complex multimorbidity, which becomes increasingly common from age 45 years. In the Finnish cohorts, there was a three times increased risk of a first obesity-related disease in people with obesity compared with people with healthy weight, which rose to more than 12 times increased risk for the fourth obesity-related disease. Corresponding risk ratios in the older UK Biobank participants were three times and 11 times increased risk in people with class 3 obesity compared with people with healthy weight, respectively. A growing difference between participants with healthy weight and those with obesity as multimorbidity increases might result from a cascade in which obesity contributes to the development of the first obesity-related disease, but both obesity and accumulation of obesity-related disease contribute to the development of all subsequent obesity-related diseases.

Our results should be interpreted in the context of the study's limitations. Baseline survey participation varied between 40% and 69% in the Finnish cohort studies and was only 5·5% in the UK Biobank cohort. Low participation might contribute to overestimation or underestimation of true associations between risk factors and health, although substantial bias is less likely to be introduced by low participation than by a large number of dropouts during follow-up, which we largely avoided.^29^ Our application of a data-driven HR cutoff point to a predefined list of diseases of public health importance limits our definition of obesity-related complex multimorbidity to diseases both robustly associated with obesity and common causes of disease burden, disability, and reduced quality of life. The exclusion of diseases that were causally but less strongly associated with obesity, such as gastrointestinal cancer, means our results are likely to underestimate the disease burden associated with obesity. Use of electronic health records means that our study shares the limitations of most other multimorbidity studies and did not include undiagnosed conditions and those that seldom lead to hospitalisation. The UK Biobank additionally missed disease cases diagnosed and treated in primary care, including diabetes and hypertension, which were captured in the Finnish cohorts via drug reimbursement data. This limitation suggests that our findings are likely to underestimate the true incidence of obesity-related multimorbidity, although relative differences between healthy weight and other groups might not be largely distorted.^30^, ^31^ Analysis of incident disease diluted the effect estimates in the UK Biobank cohort, which did not cover obesity-related disease before age 38 years. Study participants did not include children and older adults. Thus, further research is warranted to examine the generalisability of our findings to all age groups.

These findings have clinical and public health implications. Due to the accumulation of disparate diseases, complex multimorbidity presents a difficult prevention target if each disease is approached separately.^23^ The strong link between obesity and multimorbidities from a range of cardiometabolic, digestive, respiratory, neurological, musculoskeletal, and infectious diseases suggests obesity is an important, amenable target for disease prevention that avoids the burden of multitarget regimens. The creation of healthy living environments with less factors that predispose to obesity presents a parsimonious approach to reduce multimorbidity at the population level, while obesity treatments, such as lifestyle interventions, pharmacotherapy, and bariatric surgery could prevent multimorbidity among those who receive them.

## Data sharing

In the FPS and HeSSup studies, pseudonymised questionnaire data as used in this study can be shared upon request to the investigators. Linked health records require separate permission from the National Institute of Health and Welfare and Statistics Finland. Researchers registered with UK Biobank can apply for access to the database by completing an application, which must include a summary of the research plan, data fields required, any new data or variables that will be generated, and payment to cover the incremental costs of servicing an application (https://www.ukbiobank.ac.uk/enable-your-research/apply-for-access). Statistical code and complete summary data for all figures and tables are provided in the appendix (pp 12–17).

## Declaration of interests

TS reports cooperation (consultative, educational, research) with companies and entities interested in cardiovascular prevention and diabetes (not directly obesity), including Amgen, Pfizer, MSD, Orion Pharma, Sanofi, Sankyo, and NovoNordisk. All other authors declare no competing interests.
