# Supplementary material for: Body-mass index and risk of obesity-related complex multimorbidity: an observational multicohort study
Source: Lancet Diabetes Endocrinol. 2022 Apr;10(4):253–63. doi: 10.1016/S2213-8587(22)00033-X (PMC8938400; doi:10.1016/S2213-8587(22)00033-X)
Supplement: Supplementary appendix [file mmc1.pdf]

THE LANCET
Diabetes & Endocrinology

Supplementary appendix

This appendix formed part of the original submission and has been peer reviewed. We post it as supplied by the authors.

Supplement to: Kivimäki M, Strandberg T, Pentti J, et al. Body-mass index and risk of obesity-related complex multimorbidity: an observational multicohort study. *Lancet Diabetes Endocrinol* 2022; published online March 3. https://doi.org/10.1016/S2213-8587(22)00033-X.

CONTENTS

Extended methods 3

Study population 3

Supplement figure 1. Timeline for data collection by cohort 4

Baseline assessment 4

Follow-up for morbidity and mortality 4

Supplement table 1. Hierarchy of ICD-10 codes and disease frequencies for 78 health outcomes in Finnish cohorts 6

Supplement table 2. The Social Insurance Institution of Finland Clinical criteria for eligibility of drug reimbursement for obesity-related diseases 7

Data analysis 7

Supplement table 3. Test of the proportional hazards assumption: interaction between log(time) and obesity 8

Supplement figure 2. Log-log plot: log(follow-up) versus log(-log(survival)) for outcomes with unmet proportionality assumption 9

Statistical code 12

Additional results 17

Single diseases in Finnish cohorts 17

Supplement table 4. Associations of obesity versus normal weight with 78 health outcomes in Finnish cohorts 18

Supplement table 5. Associations of overweight versus normal weight with 78 health outcomes in Finnish cohorts 19

Supplement table 6. Associations of underweight versus normal weight with 78 health outcomes in Finnish cohorts 20

Obesity-related multimorbidity in Finnish cohorts 21

Supplement table 7. Associations of BMI category with incident obesity-related disease and multimorbidity in FPS and HeSSup cohorts 21

Supplement table 8. Associations of BMI category with incident obesity-related disease and multimorbidity based on pooled analysis and meta-analysis in Finnish cohorts 21

Supplement table 9. Lifestyle-adjusted associations of BMI category with incident obesity-related disease and multimorbidity in Finnish cohorts 22

Supplement table 10. Associations of BMI category with incident obesity-related disease and multimorbidity using alternative reference group of normal weight participants with BMI 22.5 to <25kg/m^2 in Finnish cohorts 23

Supplement table 11. Associations of BMI category with incident obesity-related disease and multimorbidity using alternative definitions of multimorbidity in Finnish cohorts 23

Supplement table 12. Associations of BMI category with incident obesity-related disease and multimorbidity by sex in Finnish cohorts 24

Supplement table 13. Associations of BMI category with incident obesity-related disease and multimorbidity by age group in Finnish cohorts 24

Supplement figure 3. Temporal associations between obesity-related disease pairs in Finnish cohorts 25

Supplement table 14. Age-, sex-, cohort-, education- and neighbourhood deprivation-adjusted associations between obesity-related disease pairs in Finnish cohorts 26

Supplementary table 15. Frequency of disease combinations in complex multimorbidity (the first 4 diseases irrespective of temporal order) 31

Supplementary table 16. Proportions of obesity-related diseases in obese participants by number of obesity-related diseases 34

Supplement table 17. Associations of BMI category with the rate of obesity-related diseases in Finnish cohorts 35

Supplement table 18. Difference in hazard of multimorbidity between participants with overweight and obesity compared to normal weight in Finnish cohorts 35

External replication in UK Biobank 36

Supplement figure 4. Age distribution at BMI assessment in the Finnish cohorts and UK Biobank 36

Supplement table 19. Associations of obesity versus normal weight with 78 health outcomes in UK Biobank 37

Supplement table 20. Lifestyle-adjusted associations of BMI category with incident obesity-related disease and multimorbidity in UK Biobank 38

Supplement table 21. Comparison of findings between Finnish cohorts and UK Biobank 39

Discussion 40

Supplement table 22. Mendelian randomisation evidence on causality for observed obesity-related diseases 41

Appendix references 42

Extended methods

Study population

In HeSSup 64,797 men and women were sent a survey between 1998 and 2000 or in 2003. Responders were linked electronically to national hospitalisation and mortality registers.[1]

Men and women participating in HeSSup were from a stratified random sample of the Finnish population based on four age groups (20-24, 30-34, 40-44, and 50-54). The eligible population (N=64,797) was identified from the Finnish population register and an invitation to participate was posted along with a baseline questionnaire. Between June 7, 1998 and May 23, 1999 and January 7 and August 12, 2003, 23,988 responded, provided data on BMI and were successfully linked to electronic health records from national registers until December 31, 2015. The Turku University Central Hospital Ethics Committee approved the study.

The study population of FPS comprised 113,578 men and women who were sent surveys between 2000 and 2002, 2004 and 2005, 2008 and 2009, and/or 2011 and 2013. Study participants were linked to electronic health records until December 31, 2018.

The FPS sample comprised the entire public sector personnel of 10 cities and 21 hospitals in the same geographical areas.[2] The participants had a job contract of at least 6 months between 1990 and 2005 and were eligible for at least one of the four surveys conducted between March 1, 2000 and June 30, 2002; March 1, 2004 and June 30, 2005; March 1, 2008 and November 30, 2009, December 1, 2011 and November 30, 2013. The sample included in the present analysis comprised 90,669 men and women aged 17 to 78 who responded to the survey, had data on BMI, and were successfully linked to electronic health records from national registers up to December 31, 2018. Helsinki Uusimaa Hospital District Ethics Committee approved the study.

Study population in the Finnish cohorts was ethnically homogeneous. According to linked Population Register records on primary language (the official language spoken in Finland versus not), less than 1% of participants had an immigrant background.

To examine the robustness and generalisability of our findings, we repeated and expanded the main analyses in an independent cohort study, UK Biobank. The study population included 502,665 UK adults participating in a baseline examination (2006–2010). Hospital admissions and deaths were followed via national health registers.

UK Biobank is a large-scale biomedical database and research resource, containing in-depth health information from half a million UK participants (www.ukbiobank.ac.uk, last accessed 15 December 2021).[3] Approximately 9.2 million people were invited to ensure ~500,000 participants. The present analysis was based on 499,357 men and women, age 38 to 72, with data on BMI measured during a clinical examination at baseline between 13-Mar-2006 and 1-Oct-2010 and linked electronically to the UK National Health Service's Hospital Episode Statistics database. Follow-up of hospital admissions and deaths was until 31-Mar-2021. The study was conducted under generic approval from the National Health Service National Research Ethics Service (June 17, 2011; Ref 11/NW/0382) and Material Transfer Agreement with Reference Number 60565. Ethical approval for these studies was obtained from local committees on the ethics of human research. Analyses of UK Bank were done under generic approval from the National Health Service National Research Ethics Service (2CFFAA23-CEC4-4AF0-9133-405139170B01). Supplement figure 1 shows timeline for data collection in FPS, HeSSup and UK Biobank.

Supplement figure 1. Timeline for data collection by cohort

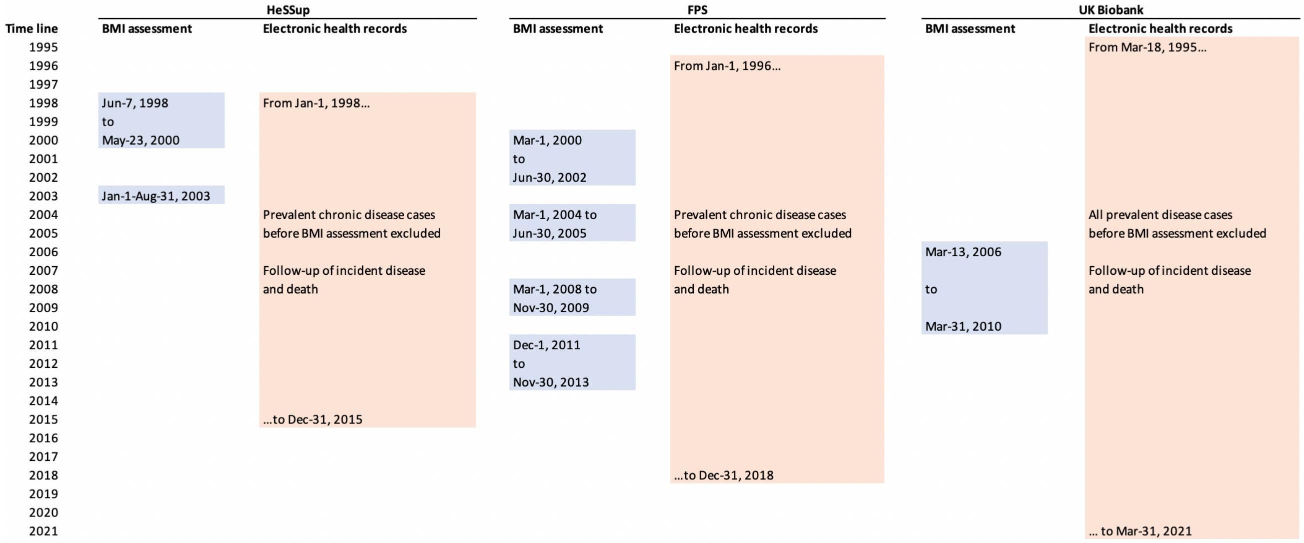

Baseline assessment

Weight and height at baseline were self-reported in HeSSup and FPS and were measured in UK Biobank. We calculated BMI using the formula:[4] weight in kg divided by height in metres squared and defined obesity as BMI equal to or greater than 30kg/m^2, overweight as BMI between 25 and 29.9 kg/m^2, normal weight as BMI between 18.5 and 24.9kg/m^2 and underweight as BMI lower than 18.5 kg/m^2. Obesity was further divided into class-1 (BMI 30–34.9kg/m^2), class-2 (35–39.9kg/m^2) and class-3 (40kg/m^2 or higher). Self-reported BMI tends to be underestimated, especially in the overweight and obese.[5]

In addition to age, sex and cohort, baseline characteristics included education and neighbourhood deprivation as these factors have been shown to correlate with death and a wide range of diseases,[6,7] predict the development of obesity[8] and, in social experiments, reduction of deprivation has been associated with subsequent reduction in obesity.[9] In combination, this evidence suggests that education and neighbourhood deprivation may act as confounding factors increasing the risk of both obesity and adverse health outcomes. Using predefined operationalisations, educational attainment, obtained from Statistics Finland via record linkage (FPS) or by survey (HeSSup, UK Biobank), was based on three categories: primary, secondary and tertiary qualification. Neighbourhood deprivation scores for HeSSup and FPS were obtained from Statistics Finland based on the proportion of adults with low education, the unemployment rate, and the proportion of people living in rented housing in each 250m x 250m grid area.[6] For each of the three variables, we derived a standardized z score based on the total Finnish population (mean=0, SD=1). A score for neighbourhood deprivation was then calculated by taking the mean value across the three z scores. Higher scores on the continuous index denote greater disadvantage.[6] In UK Biobank, continuous Townsend index was used to assess neighbourhood deprivation.[10]

Further covariates included lifestyle factors which were obtained using standard questionnaire measures and were categorised using predefined harmonised operationalisations across the three cohorts.[11,12] current smoking (yes vs no), heavy drinking (>21 alcoholic drinks per week for men and >14 for women versus 0-21 drinks for men and 0-14 drinks for women), and physical activity (low [no or little moderate/vigorous leisure-time physical activity] versus high [much or some moderate/vigorous leisure-time physical activity]).

Follow-up for morbidity and mortality

Participants from HeSSup and FPS were linked by their unique identification number to national registries of hospital discharge information (recorded by the Finnish Institute for Health and Welfare) and mortality (recorded by Statistics Finland). These electronic health records included cause and date of hospitalisation and/or mortality from 1-Jan-1996 up to 31-Dec-2018 and their coverage (all hospital types, including private hospitals, and emergencies) reflects the comprehensive nature of Finland's public health care system. Additional information on site-specific cancers, diabetes, cardiovascular diseases (including hypertension), psychotic disorder, dementia, Parkinson's disease, multiple sclerosis, epilepsy, asthma, chronic obstructive

pulmonary disease, inflammatory bowel disease, liver disease, rheumatoid arthritis, gout, and renal failure was available via record linkage to the Drug Reimbursement Register of the Social Insurance Institution of Finland (1-Jan-1980 to 30-Dec-2018). In UK Biobank, study participants were linked to the UK National Health Service's Hospital Episode Statistics (HES) database for hospital admissions and the NHS Central Registry for mortality from 18-Mar-1995 to 31-Mar-2021.

In all three cohort studies, diseases obtained from hospital records were coded according to the World Health Organization's International Classification of Diseases 10th Revision (ICD-10) which captures a total of 1204 3-digit diagnostic codes. Excluding hospitalisation due to obesity, we focussed on a pre-defined list of 78 common ICD-10 disease chapters and diagnostic groups constructed for outcome-wide studies by investigators blinded to exposure data including BMI (supplement table 1).[6,13,14]

Supplement table 1. Hierarchy of ICD-10 codes and disease frequencies for 78 health outcomes in Finnish cohorts

	ICD-10 code hierarchy					Additional drug	N
Disease	Level 1	Level 2	Level 3	Level 4	External cause	reimbursement data	(incident cases)
**Infections**	**A01-B89**						3446
Bacterial infections		A01-A79					2953
Viral infections		A80-B34					532
**Cancer**	**C00–C97**						6168
Colorectal cancer		C18, C20				✓	563
Lung cancer		C34				✓	319
Melanoma		C43–C44				✓	1303
Breast cancer		C50				✓	2563
Prostate cancer		C61				✓	570
Kidney cancer		C64				✓	152
Brain cancer		C71				✓	143
Leukaemia, lymphoma		C81–C96				✓	653
**Diseases of the blood**	**D50–D89**						706
Anaemia		D50–D64					396
**Endocrine diseases**	**E00–E90**					✓	6757
Diabetes		E10-E14				✓	5819
**Mental and behavioural disorders**	**F00–F99**						2440
Dementia		F00–F03, G30, G31				✓	543
Disorders due to substance abuse		F10–F19					728
Psychotic disorders		F20–F29				✓	858
Mood disorders		F30–F39					1174
Neurotic disorders		F40–F48					422
**Diseases of the nervous system**	**G00–G99**						6282
Parkinson disease		G20				✓	284
Multiple sclerosis		G35				✓	277
Epilepsy		G40-G42				✓	647
Headaches		G43-G44					424
TIA		G45-G46					827
Sleep disorders		G47				✓	2654
**Diseases of the eye**	**H00–H59**						5789
**Diseases of the ear**	**H60–H99**						1130
**Diseases of the circulatory system**	**I00-I99**						10220
Hypertension		I10–I15				✓	6190
Ischemic heart diseases		I20–I25				✓	2863
Angina pectoris			I20			✓	1213
Myocardial infarction			I21			✓	1000
Pulmonary embolism		I26					440
Arrhythmias		I46-I49				✓	4252
Heart failure		I50				✓	570
Cerebrovascular diseases		I60–I69					1538
Stroke			I60-I61, I63-I64				1281
Intracerebral haemorrhage				I61			223
Cerebral infarction				I63			873
Arteriosclerosis				I70			261
Deep vein thrombosis		I80-I82					506
**Diseases of the respiratory system**	**J00–J99**						7192
Influenza and Pneumonia		J09–J18					2487
Chronic obstructive bronchitis		J43-J44, J47				✓	513
Asthma		J45-J46				✓	3655
**Diseases of the digestive system**	**K00–K93**						11870
Appendicitis		K35					1421
Inflammatory bowel disease		K50–K52				✓	924
Diseases of liver		K70–K77					844
Alcoholic liver disease			K70				229
Pancreatitis		K85					339
**Diseases of the skin**	**L00-L99**						1250
Skin infections and excema		L00-L08, L20-L30					633
**Diseases of the musculoskeletal system**	**M00-M99**						16457
Rheumatoid arthritis and related disorders		M05-M06, M08, M13, M30-35, M45				✓	2316
Gout		M10				✓	254
Osteoarthritis		M15–M19					5597
Sciatica		M50-M51					1640
Back pain		M54					769
Soft tissue disorders		M60–M79					5350
**Diseases of the genitourinary system**	**N00-N99**						11784
Renal failure		N17–N19				✓	350
**Pregnancy complications**	**O00-O03, O05-O29**						2648
Spontaneous abortion		O03					503
Hypertension in pregnancy		O13–O16					472
Diabetes in pregnancy		O24					521
**Miscellaneous**							
Circulatory and respiratory symptoms		R00–R09					2074
Digestive and abdominal symptoms		R10–R19					2497
Injury		S00-T35					10187
Poisoning		T36-T65					732
Road accidents					V01-V99		720
Falls					W00-W19		4162
Self-harm					X60-X84		378
**Death**							4083

The Social Insurance Institution of Finland Clinical criteria for eligibility of drug reimbursement for obesity-related diseases were as follows:

Supplement table 2. The Social Insurance Institution of Finland Clinical criteria for eligibility of drug reimbursement for obesity-related diseases

Disease	Diagnostic criteria
Diabetes	Fasting plasma glucose $\geq$7.0mmol/l, 2-h postload plasma glucose $\geq$11.1mmol/L or blood HbA1c$\geq$6.5% and symptoms of diabetes (increased thirst, unplanned weight loss, frequent urination)
Hypertension	Non-response to lifestyle intervention in 6 months and systolic/diastolic blood pressure before medication $\geq$180/95mmHg, or $\geq$160/95mmHg if comorbid left ventricular hypertrophy, diabetes or signs of blood-pressure related organ damage.
Angina pectoris	Diagnosis based on clinical examination (ECG, stress test).
Myocardial infarction	History of myocardial infarction and treatment.
Gout	Diagnosis made by an internist/rheumatologist.
Asthma	A 12% increase in either FEV1 (forced exhalation volume in one second) or FVC (forced vital capacity) and a 200 ml increase in FEV1 or FVC; $\geq$20% daily change in PEF (peak expiratory flow) measurement (min 3 repeats) compared to morning or evening average in the same day; 15% increase in PEF as a result of corticosteroid treatment; reduction of 15% in physical challenge tests; Severe or moderately severe bronchoconstriction (narrowing or spasm of airways) in histamine/ methacholine challenge test.
Renal failure	Uraemia requiring dialysis, diagnosed by a specialist.
Kidney cancer	Diagnosis based on imaging tests (x-rays, magnetic fields, sound waves, or radioactive substances) or kidney biopsy.

Data analysis

After gaining permission to use electronic health records as research data and receiving the raw data, we assessed data quality in terms of completeness and accuracy. Ascertainment of data element completeness included examining metadata, such as a data dictionary, contained in the dataset against the requested variables required for the statistical analysis. In assessing accuracy, we compared the data to external sources, such as reported national disease and mortality statistics. Internal consistency checks and curation included computed logic checks for the correctness of linkage identifiers across electronic health records and the clinic and survey data and over time, detection of outlier values or impossible records, such as a date of death occurring before hospital admission, and implausible changes in clinical characteristics over time (e.g. a large increase in height in adulthood), and assessment of inconsistencies in records across electronic health records and clinical/survey assessment. Data were pseudonymised/anonymised before research use.

Participants with morbidity and mortality follow-up and no missing data on age, sex, and BMI were included in the analysis. Missing data were treated as a separate category for other covariates. We used pooled individual-level data from the two Finnish cohort studies for primary analysis. We assessed the proportional hazards assumption for the associations of obesity vs normal weight for each of the 78 health outcomes and found no major violations. Supplement table 3 and supplement figure 2 show that although the interaction term was statistically significant for 18 health outcomes, any differences in hazard ratios for the corresponding obesity-health outcome associations were not extreme between the two periods of follow-up, a finding also confirmed by the log-log plots. Non-significant associations remained so. There was dilution of the effect over time for many health outcomes, including diabetes, sleep disorders, asthma, gestational diabetes, pregnancy complications and injury. For some health outcomes the association with obesity strengthened over time. These were skin diseases (such as skin infections and eczema), back pain, diseases of the circulatory and genitourinary systems.

Supplement table 3. Test of the proportional hazards assumption: interaction between log(time) and obesity

					Hazard ratio (95% CI)	
					Follow-up period	
**Disease**	**Total**	**Event**	**ChiSq (df=1)**	**ProbChiSq**	**Years 0 to 5**	**Years 5+**
Infections	82078	2413	3.9	0.0493	1.97 (1.71-2.27)	2.04 (1.83-2.27)
Bacterial infections	82365	2077	2.5	0.1115	2.27 (1.95-2.65)	2.11 (1.89-2.37)
Viral infections	83069	367	0.1	0.7982	0.93 (0.64-1.37)	1.30 (0.95-1.79)
Cancer	82263	4264	1.8	0.1755	1.02 (0.91-1.15)	1.12 (1.03-1.21)
Colorectal cancer	83309	375	1.4	0.2401	0.77 (0.48-1.26)	1.26 (0.98-1.62)
Lung cancer	83351	197	0.0	0.9815	0.59 (0.31-1.10)	0.59 (0.39-0.88)
Melanoma	83255	898	1.5	0.2147	1.43 (1.03-1.98)	0.98 (0.82-1.16)
Breast cancer	65566	1883	1.6	0.2113	0.95 (0.79-1.13)	1.01 (0.88-1.15)
Prostate cancer	17175	310	0.6	0.4402	1.12 (0.69-1.82)	0.98 (0.74-1.30)
Kidney cancer	83340	97	1.0	0.3119	2.80 (1.25-6.27)	1.21 (0.72-2.04)
Brain cancer	83347	107	0.0	0.9272	1.20 (0.56-2.54)	0.83 (0.47-1.47)
Leukaemia, lymphoma	83212	423	0.0	0.9841	1.21 (0.82-1.78)	1.15 (0.89-1.50)
Diseases of the blood	83123	489	0.3	0.5691	1.86 (1.36-2.55)	1.75 (1.37-2.23)
Anemia	83246	269	0.0	0.8623	1.78 (1.12-2.83)	1.64 (1.20-2.25)
Endocrine diseases	81702	4795	0.4	0.5033	8.27 (7.29-9.37)	8.31 (7.70-8.97)
Diabetes	82117	4130	24.0	<0.0001	17.0 (14.2-20.3)	11.1 (10.2-12.1)
Mental and behavioural disorders	82384	1754	4.1	0.0417	1.02 (0.87-1.21)	1.09 (0.94-1.28)
Dementia	83342	345	0.5	0.4959	0.70 (0.39-1.27)	0.78 (0.59-1.01)
Disorders due to substance abuse	83171	524	2.1	0.1493	0.70 (0.51-0.97)	1.05 (0.80-1.37)
Psychotic disorders	82736	629	0.0	0.9480	1.04 (0.79-1.37)	1.03 (0.79-1.34)
Mood disorders	82870	849	0.0	0.8451	1.30 (1.03-1.63)	1.06 (0.84-1.33)
Neurotic disorders	83143	314	0.8	0.3768	1.04 (0.71-1.52)	1.17 (0.79-1.73)
Diseases of the nervous system	81231	4314	13.4	0.0002	2.05 (1.85-2.27)	1.72 (1.58-1.87)
Parkinson disease	83311	182	0.0	0.8976	0.72 (0.36-1.42)	0.63 (0.41-0.97)
Multiple sclerosis	83253	200	2.7	0.0991	1.18 (0.70-1.99)	0.54 (0.30-1.00)
Epilepsy	82554	440	0.6	0.4518	1.17 (0.82-1.68)	1.07 (0.81-1.40)
Headaches	83158	301	0.7	0.4172	0.49 (0.30-0.79)	1.02 (0.68-1.53)
TIA	83264	549	0.0	0.9836	1.38 (0.99-1.91)	1.23 (0.98-1.54)
Sleep disorders	82854	1926	30.7	<0.0001	8.85 (7.16-10.9)	5.65 (5.04-6.33)
Diseases of the eye	82410	3942	0.4	0.5202	1.22 (1.06-1.39)	1.21 (1.11-1.31)
Diseases of the ear	82841	803	0.0	0.8860	1.23 (0.97-1.56)	1.16 (0.94-1.44)
Diseases of the circulatory system	79238	6978	18.2	<0.0001	1.30 (1.20-1.40)	1.61 (1.51-1.73)
Hypertension	78318	4135	10.0	0.0015	3.40 (3.09-3.73)	3.03 (2.78-3.31)
Ischemic heart diseases	82744	1818	0.7	0.4159	1.34 (1.13-1.60)	1.41 (1.25-1.59)
Angina pectoris	82948	761	0.5	0.4811	1.39 (1.08-1.79)	1.62 (1.34-1.95)
Myocardial infarction	83223	606	1.4	0.2289	1.52 (1.13-2.03)	1.52 (1.24-1.87)
Pulmonary embolism	83273	323	0.5	0.4945	3.27 (2.11-5.07)	2.71 (2.06-3.55)
Arrhythmias	82747	2945	5.9	0.0151	1.99 (1.70-2.33)	1.63 (1.49-1.79)
Heart failure	83242	386	0.6	0.4552	4.21 (2.71-6.52)	4.17 (3.24-5.38)
Cerebrovascular diseases	83137	1013	0.2	0.6705	1.35 (1.06-1.72)	1.25 (1.06-1.47)
Stroke	83186	847	0.5	0.4837	1.49 (1.14-1.94)	1.37 (1.15-1.63)
Intracerebral haemorrhage	83336	157	1.0	0.3235	1.73 (0.97-3.10)	1.26 (0.83-1.91)
Cerebral infarction	83254	562	0.0	0.8344	1.54 (1.09-2.17)	1.50 (1.22-1.84)
Arteriosclerosis	83321	167	2.1	0.1457	0.57 (0.31-1.03)	0.93 (0.62-1.40)
Deep vein thrombosis	83156	362	2.9	0.0889	3.26 (2.34-4.55)	1.93 (1.44-2.59)
Diseases of the respiratory system	78671	5188	3.9	0.0481	1.37 (1.24-1.51)	1.34 (1.23-1.46)
Influenza and Pneumonia	82677	1794	1.4	0.2293	1.51 (1.26-1.80)	1.42 (1.25-1.62)
Chronic obstructive bronchitis	83298	343	0.0	0.8923	1.22 (0.76-1.95)	1.02 (0.78-1.33)
Asthma	79690	2636	5.2	0.0229	2.14 (1.88-2.44)	1.82 (1.63-2.03)
Diseases of the digestive system	78567	8244	0.4	0.5098	1.64 (1.52-1.77)	1.53 (1.43-1.63)
Appendicitis	82516	1047	0.3	0.6161	1.21 (0.97-1.51)	1.05 (0.86-1.30)
Inflammatory bowel disease	82665	689	2.3	0.1299	1.04 (0.76-1.43)	1.25 (1.00-1.57)
Diseases of liver	83219	589	3.0	0.0844	1.57 (1.15-2.15)	1.99 (1.61-2.44)
Alcoholic liver disease	83343	147	1.0	0.3288	1.27 (0.67-2.41)	2.01 (1.33-3.03)
Pancreatitis	83251	222	2.0	0.1577	2.33 (1.42-3.83)	1.66 (1.17-2.36)
Diseases of the skin	82693	904	4.3	0.0385	1.63 (1.30-2.04)	2.39 (1.98-2.87)
Skin infections and eczema	83022	448	6.2	0.0128	1.68 (1.22-2.32)	3.27 (2.53-4.23)
Diseases of the musculoskeletal system	75613	11446	0.2	0.6672	1.40 (1.32-1.48)	1.42 (1.34-1.51)
Rheumatoid arthritis and related disorders	82046	1669	2.2	0.1336	1.22 (1.02-1.46)	1.40 (1.22-1.61)
Gout	83277	170	2.1	0.1496	4.54 (2.26-9.13)	4.26 (2.93-6.18)
Osteoarthritis	82096	3763	8.1	0.0043	3.02 (2.69-3.39)	2.56 (2.36-2.79)
Sciatica	82547	1148	3.1	0.0785	1.15 (0.94-1.41)	1.12 (0.92-1.36)
Back pain	83017	553	3.9	0.0484	1.57 (1.19-2.07)	2.10 (1.65-2.69)
Soft tissue disorders	81353	3678	1.9	0.1629	1.21 (1.09-1.35)	1.17 (1.05-1.30)
Diseases of the genitourinary system	76832	8604	10.8	0.0010	1.09 (1.01-1.17)	1.30 (1.22-1.40)
Renal failure	83310	248	0.1	0.8053	3.47 (1.89-6.34)	2.82 (2.11-3.78)
Pregnancy complications	62765	2128	18.0	<0.0001	1.42 (1.23-1.65)	1.15 (0.90-1.47)
Spontaneous abortion	65518	409	0.0	0.9312	0.95 (0.65-1.39)	1.06 (0.64-1.77)
Hypertension in pregnancy	65684	372	0.0	0.8745	1.56 (1.10-2.22)	2.11 (1.36-3.28)
Diabetes in pregnancy	65511	379	25.3	<0.0001	6.43 (4.98-8.30)	1.98 (1.20-3.28)
Circulatory and respiratory symptoms	82534	1361	0.4	0.5399	1.48 (1.24-1.76)	1.18 (1.00-1.38)
Digestive and abdominal symptoms	81838	1822	5.4	0.0197	1.34 (1.15-1.58)	1.39 (1.20-1.61)
Injury	79400	7084	2.8	0.0968	1.09 (1.00-1.19)	1.05 (0.97-1.13)
Poisoning	83155	531	2.4	0.1228	1.21 (0.91-1.61)	0.99 (0.74-1.33)
Road accidents	65643	518	0.9	0.3487	0.77 (0.56-1.06)	0.95 (0.71-1.26)
Falls	64119	2903	2.3	0.1270	1.23 (1.09-1.40)	1.09 (0.98-1.22)
Self-harm	65867	262	1.0	0.3203	1.38 (0.92-2.05)	1.04 (0.70-1.55)
Death	83358	2064	1.0	0.3088	1.36 (1.14-1.63)	1.31 (1.17-1.46)

Supplement figure 2. Log-log plot: log(follow-up) versus log(-log(survival)) for outcomes with unmet proportionality assumption

Infections

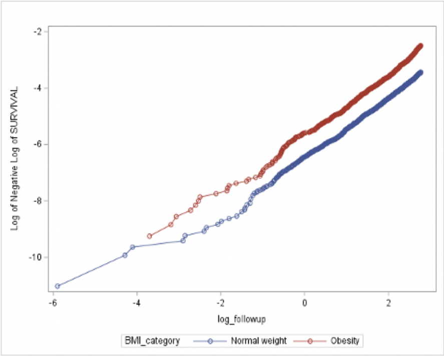

Diabetes

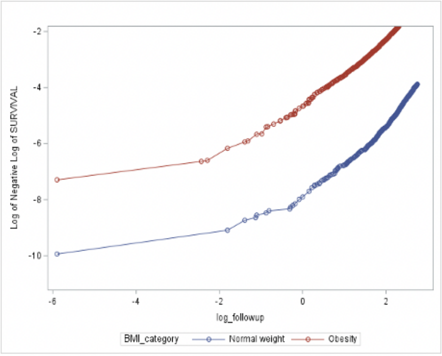

Mental and behavioural disorders

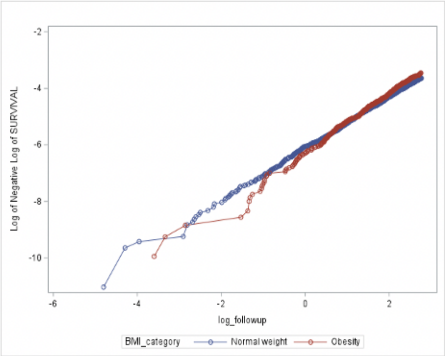

Diseases of the nervous system

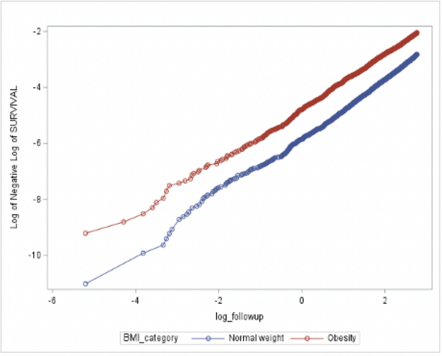

Sleep disorders

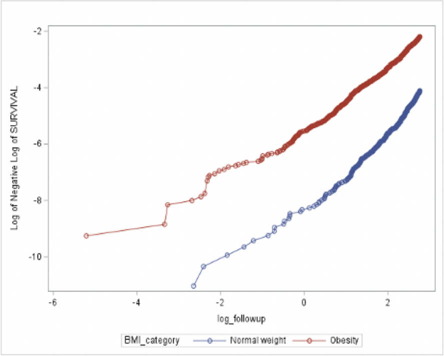

Diseases of the circulatory system

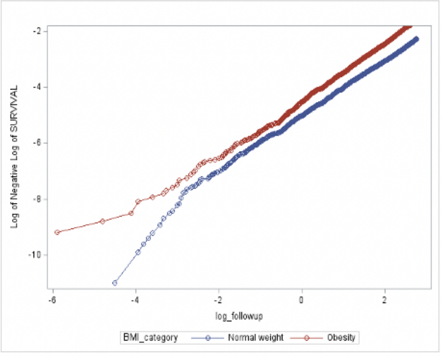

Hypertension

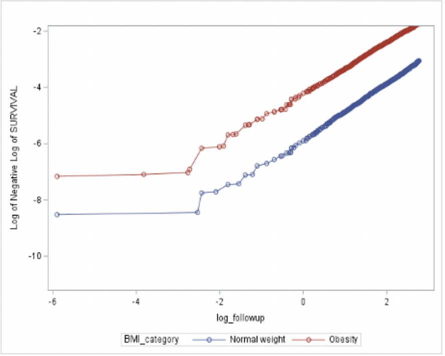

Arrhythmias

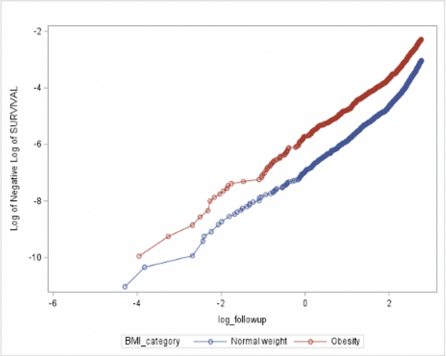

Diseases of the respiratory system

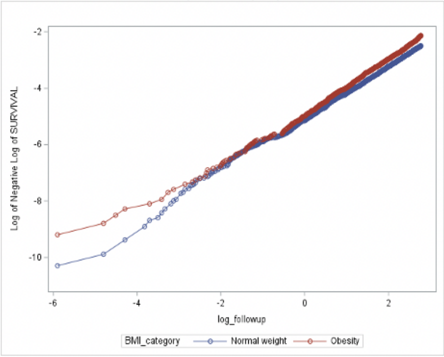

Asthma

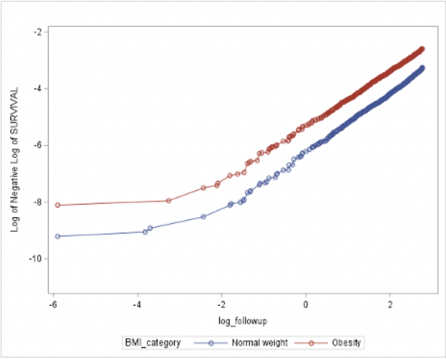

Diseases of the skin

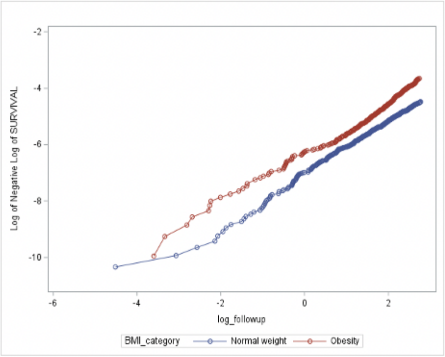

Skin infections and eczema

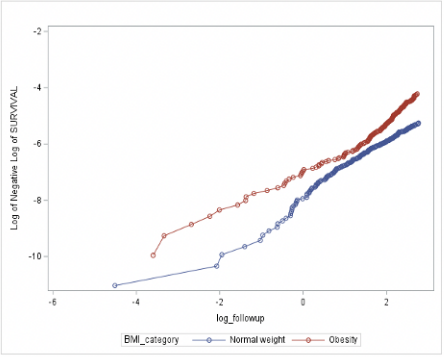

Osteoarthritis

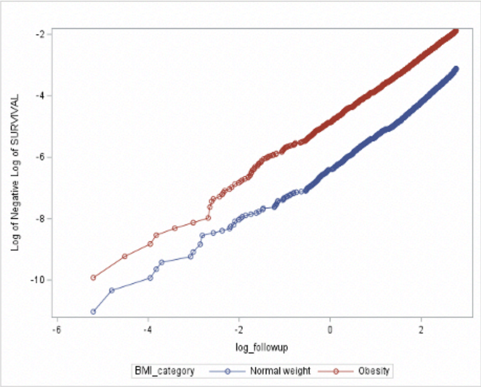

Back pain

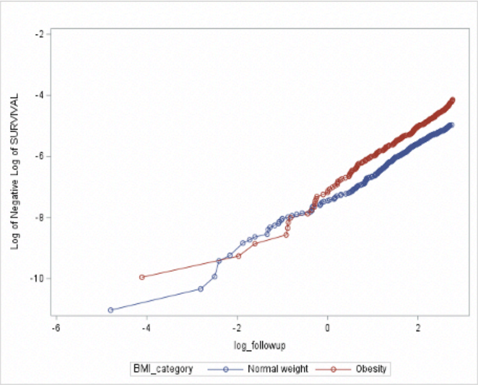

Diseases of the genitourinary system

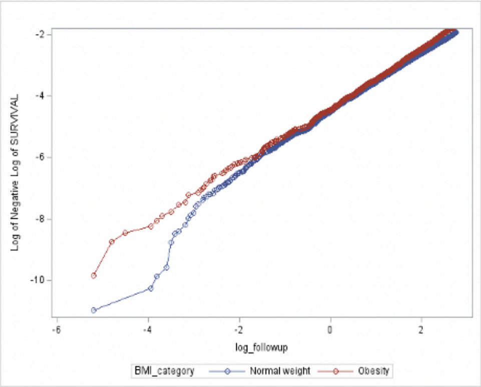

Pregnancy complications

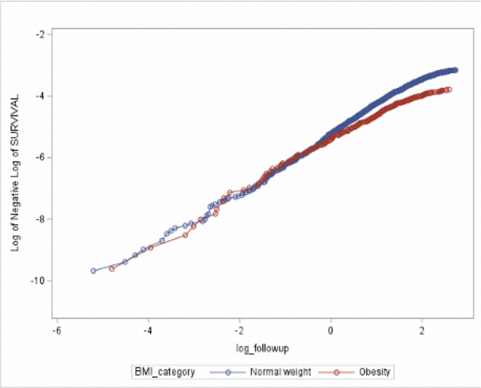

Diabetes in pregnancy

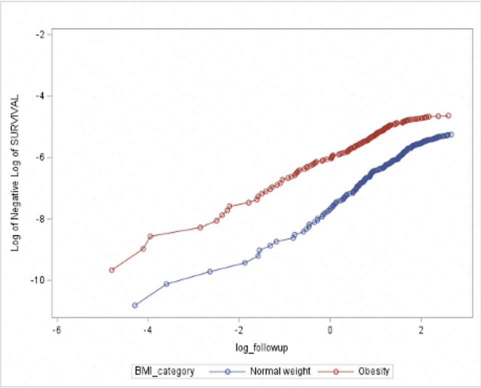

Digestive and abdominal symptoms

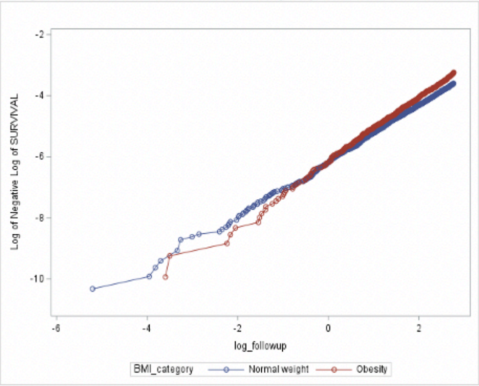

We examined associations between obesity and the 78 diseases in separate models using Cox proportional hazards regression. In these analyses, follow-up started from BMI assessment and continued until the onset of the disease of interest, date lost to follow-up, death, or end of follow-up, whichever came first. Hazard ratios (HRs) and 95% confidence intervals (CI) computed for obesity with normal weight as the reference, were adjusted for age, sex, education, neighbourhood deprivation and cohort (the basic model). To focus on diseases that are more common in participants with obesity and relate to differences in disease risk that are likely to be meaningful for public health and health care, we considered only obesity-disease associations which yielded hazard ratios $\geq$1.5[15] and were statistically significant at a Bonferroni corrected alpha-level, $P<6.3x10^{-4}$ (0.05/78 tests). Sex differences in the associations between obesity and disease outcomes were examined by including a 'sex x BMI' interaction term in a Cox model in addition to their main effects and adjustments as in the basic model.

Further analyses focussed on these obesity-related diseases after excluding overlapping conditions. To examine obesity in relation to the co-occurrence of obesity-related diseases, we constructed 4 disease outcomes: onset of the first, second, third and fourth obesity-related disease. The second outcome (2 obesity-related diseases) refers to 'simple multimorbidity" and the last outcome (4 or more obesity-related diseases) refers to 'complex multimorbidity'.[16,17] The number of new onset obesity-related diseases accrued by the end of follow-up or death determine the allocation of participants to each of the 4 health outcomes. To assess dose-response patterns within the obesity category, we stratified obesity into classes 1, 2 and 3. In addition to HRs and 95% CIs, we calculated population attributable fraction (PAF) to evaluate potential reduction in obesity-related multimorbidity were exposure to obesity removed.

To further increase understanding of obesity-related multimorbidity, we examined temporal sequences in the emergence of obesity-related diseases by testing prospective associations between all obesity-related disease pairs in individuals with obesity. For each disease pair we tested the extent to which Disease A in participants with no Disease B predicted Disease B over the follow-up period and the extent to which Disease B in participants with no Disease A predicted Disease A over the follow-up period. Follow-up started at recorded diagnosis for the first disease and continued until the date of diagnosis for the next disease, death, or end of follow-up, whichever came first. HRs and 95% CIs were adjusted for age, sex, cohort, education and neighbourhood deprivation.

To describe patterns of complex multimorbidity, we computed the frequency of each obesity-related disease in participants with obesity who developed four or more obesity-related health outcomes during the follow-up and calculated the proportion of disease combinations from one, two, three and four different disease categories. In addition, we listed all combinations of the first four diseases in this group and provided their frequencies and proportions of diseases from different disease categories included in these disease combinations.

We performed several sensitivity and subgroup analyses. First, to examine whether the association between BMI and multimorbidity differs between the two Finnish cohort studies, we repeated analyses of developing one, two, three or four or more obesity-related diseases separately for FPS and HeSSup. We also examined whether age-, sex-, cohort-, education and neighbourhood deprivation-adjusted estimates obtained from pooled individual-level data from the two cohorts differ from those obtained from fixed-effect meta-analysis of cohort-specific effect estimates by comparing results from these two statistical approaches.

Second, we examined whether the associations of BMI categories with multimorbidity were reproducible with two alternative definitions of multimorbidity. In the first definition, all diseases statistically significantly associated with obesity after Bonferroni significance were included as components of multimorbidity. These included diabetes, hypertension, angina pectoris, heart failure, myocardial infarction, arrhythmias, deep vein thrombosis, pulmonary embolism, cerebral infarction, anaemia, asthma, sleep disorders, back pain, osteoarthritis, gout, bacterial infections, skin infections and eczema, liver disease, renal failure, pancreatitis, influenza & pneumonia, diseases of the eye, rheumatoid arthritis and related disorders, soft tissue disorders, circulatory & respiratory symptoms, and digestive & abdominal symptoms. The second multimorbidity definition was based on disease categories rather than specific diseases. This outcome included the following ICD-10 disease chapters: Endocrine diseases, infections, cancers, diseases of the blood, eye, ear and skin, and diseases of the circulatory, digestive, genitourinary, musculoskeletal, respiratory and nervous systems. For both definitions, we constructed 4 health outcomes: onset of the first, second, third and fourth obesity-related disease.

Third, to describe the association between BMI and multimorbidity with alternative statistical methods, we repeated the analysis using Poisson regression with count of diseases as the outcome and rate ratios comparing BMI-categories (reference: normal weight) as effect estimates and Aalen additive hazard model estimating hazard differences per 10,000 person-years between BMI categories.[18,19]

Fourth, as the reference group of participants with normal weight covers a wide range of BMI-levels, we examined whether the associations with multimorbidity are replicable using a more homogeneous comparison group. In agreement with large-scale mortality analyses, we chose the BMI category 22.5 to <25 kg/m^2 as the reference in this sensitivity analysis.[20]

Fifth, to examine whether age modifies the association between BMI and obesity-related multimorbidity, we stratified analysis by age group (BMI assessment before age 50 versus BMI assessment at age 50 or older) and tested whether the estimates differed between the two age groups. A corresponding analysis was performed to test whether sex is an effect modifier.

To examine reproducibility of the findings from the Finnish cohorts in an independent external cohort and different health care setting, we repeated the main analyses in the UK Biobank cohort.

Analyses were performed using SAS statistical software version 9.4 and R version 4.0.0. The statistical code for the main analyses is as follows:

SAS (version 9.4):

```
********************************************************************;
**  Table 2  **;
********************************************************************;
** disease=dg, exposure=altiste, output file=resfile **;
** data sets: fh_taudit (diseases), hlot1 (persons) **;
********************************************************************;
%macro coxit (dg,altiste,resfile);
data tauti;
     set fh_taudit;
     IF dgnro=&dg;
data t1;
     merge hlot1(in=i) tauti;
     by tutknro;
     if i;
data t2;
     set t1;
     if &altiste>.;
     if slaalkupvm>. then status=1; else status=0;
     if kohortti=1 then seuraika=(min(slaalkupvm,kuolinpvm,mdy(12,31,2016))-alkupvm+1)/365.25;
     if kohortti=1 and &dg=79 then seuraika=(min(slaalkupvm,kuolinpvm,mdy(12,31,2018))-
alkupvm+1)/365.25;
     if kohortti=2 then seuraika=(min(slaalkupvm,kuolinpvm,mdy(12,31,2012))-alkupvm+1)/365.25;
     if kohortti=2 and &dg=79 then seuraika=(min(slaalkupvm,kuolinpvm,mdy(12,31,2015))-
alkupvm+1)/365.25;
     if exslaalkupvm>. then extauti=1;
     if extauti=1 or (sex=1 and &dg IN (8,67,68,69,70)) or (sex=2 and &dg=9)
        then do; status=.; seuraika=.; end;
     if kohortti=2 and &dg IN (75,76,77,78) then do; status=.; seuraika=.; end;
proc phreg data=t2;
     class educ ases;
     model seuraika*status(0) = sex age educ ases kohortti &altiste / rl;
     ods output ParameterEstimates=pe CensoredSummary=cs;
     data pe; set pe; if Parameter='obes2';
     data res; merge pe cs; dgnro=&dg;
     keep parameter dgnro Total Event HazardRatio HRLowerCL HRUpperCL ProbChiSq;
     data res; merge res(in=i) Tautiselitteet; by dgnro; if i;
run;
proc append base=&resfile data=res;
run;
%mend;
*** all diseases **;
proc datasets lib=work memtype=data nolist; delete results_all; quit;
%MACRO coxkaikki;
%DO I = 1 %TO 80;
    %coxit(&I,obes2,results_all);
%END;
%MEND coxkaikki;
%coxkaikki;
proc print data=results_all; where Total>.;
     id dgnro;
     var selite parameter Total Event HazardRatio HRLowerCL HRUpperCL ProbChiSq;
run;
```

```
*******************************************************************;
**  Figure 2 (Cumulative incidence)  **;
*******************************************************************;
proc phreg data=hlot5;
     model (age age_end)*status(0) = ;
     strata obes2;
     baseline out=apu1 survival=survival lower=slower upper=supper;
run;
data apu2;
     set apu1;
     rename age_end=time obes2=group;
run;
data apu3;
     set apu2;
     by group;
     retain prehazard prelower preupper 0;
     hazard=1-survival;
     lower=1-supper;
     upper=1-slower;
     if (hazard>. and hazard NE prehazard) or first.group;
     prehazard=hazard;
     prelower=lower;
     preupper=upper;
     keep group time hazard lower upper;
data apu4;
     set apu3;
     retain prehazard prelower preupper pregroup 0;
     if group=pregroup then do;
        cumhazard=prehazard; cumlower=prelower; cumupper=preupper; output; end;
     cumhazard=hazard; cumlower=lower; cumupper=upper; output;
     pregroup=group;
     prehazard=hazard; prelower=lower; preupper=upper;
     keep group time cumhazard cumlower cumupper;
data apu4;
     set apu4;
     cumhazard=100*cumhazard; ** % **;
     cumlower=100*cumlower;
     cumupper=100*cumupper;
run;
proc print data=apu4; ** => Excel figure **;
run;

proc lifetest data=hlot5 plots=(survival(atrisk) logsurv);
time seuraika*status(0);
strata obes2;
run;

*******************************************************************;
**  Table 3 (multimorbidity)  **;
*******************************************************************;
proc means data=fh_taudit nway noprint;
     where dgnro IN (2,10,15,29,33,35,36,37,38,39,43,45,49,53,55,57,60,61,63,66,80) and
exslaalkupvm>.;
     var exslaalkupvm;
     class tutknro;
     output out=pois min=;
run;
data hlot3;
     merge hlot2(in=i) pois(in=j);
     by tutknro;
     if i and NOT j;
run;
data taudit1;
     set fh_taudit;
  IF dgnro IN (2,10,15,29,33,35,36,37,38,39,43,45,49,53,55,57,60,61,63,66,80) and slaalkupvm>.;
proc sort data=taudit1;
     by tutknro;
data taudit2;
     merge hlot3(in=i) taudit1(in=j);
     by tutknro;
     if i and j;
     keep tutknro slaalkupvm dgnro obes2 BMI4;
proc sort data=taudit2;
     by tutknro slaalkupvm;
data taudit3;
     set taudit2;
     by tutknro;
     retain order 0;
     if first.tutknro then order=0;
     order=order+1;
```

```
run;
** 1.-4. disease **;
data tauti1; set taudit3;
     if order=1; rename slaalkupvm=slaalkupvm1;
data tauti2; set taudit3;
     if order=2; rename slaalkupvm=slaalkupvm2;
data tauti3; set taudit3;
     if order=3; rename slaalkupvm=slaalkupvm3;
data tauti4; set taudit3;
     if order=4; rename slaalkupvm=slaalkupvm4;
run;
*********************************************;
data hlot4;
     merge hlot3(in=i) tauti1 tauti2 tauti3 tauti4;
     by tutknro;
     if i;
run;
data hlot5;
     set hlot4;
   slaalkupvm=slaalkupvm1;
*  slaalkupvm=slaalkupvm2;
*  slaalkupvm=slaalkupvm3;
*  slaalkupvm=slaalkupvm4;
     if slaalkupvm>. then status=1; else status=0;
     if kohortti=1 then seuraika=(min(slaalkupvm,kuolinpvm,mdy(12,31,2016))-alkupvm+1)/365.25;
     if kohortti=2 then seuraika=(min(slaalkupvm,kuolinpvm,mdy(12,31,2012))-alkupvm+1)/365.25;
     age_end=round(age+seuraika,0.1);
run;
proc freq data=hlot5;
     tables BMI4*status / nopercent nocol norow;
run;
proc phreg data=hlot5;
     class educ ases BMI4(ref='2');
     model seuraika*status(0) = sex age educ ases kohortti BMI4 / rl;
run;
proc phreg data=hlot5;
     class educ ases alko2 smoke met2 BMI4(ref='2');
     model seuraika*status(0) = sex age educ ases alko2 smoke met2 kohortti BMI4 / rl;
run;

*********************************************************************;
**  Supplement (Test of the proportional hazards assumption)  **;
*********************************************************************;
** disease=dg, exposure=altiste, output file=resfile **;
** data sets: fh_taudit (diseases), hlot1 (persons) **;
*********************************************************************;
%macro coxph (dg,altiste,resfile);
data tauti;
     set fh_taudit;
     IF dgnro=&dg;
data t1;
     merge hlot1(in=i) tauti;
     by tutknro;
     if i;
data t2;
     set t1;
     if slaalkupvm>. then status=1; else status=0;
     if kohortti=1 then seuraika=(min(slaalkupvm,kuolinpvm,mdy(12,31,2016))-alkupvm+1)/365.25;
     if kohortti=1 and &dg=79 then seuraika=(min(slaalkupvm,kuolinpvm,mdy(12,31,2018))-
alkupvm+1)/365.25;
     if kohortti=2 then seuraika=(min(slaalkupvm,kuolinpvm,mdy(12,31,2012))-alkupvm+1)/365.25;
     if kohortti=2 and &dg=79 then seuraika=(min(slaalkupvm,kuolinpvm,mdy(12,31,2015))-
alkupvm+1)/365.25;
     if exslaalkupvm>. then extauti=1;
     if extauti=1 or (sex=1 and &dg IN (8,67,68,69,70)) or (sex=2 and &dg=9)
        then do; status=.; seuraika=.; end;
     if kohortti=2 and &dg IN (75,76,77,78) then do; status=.; seuraika=.; end;
     IF &altiste>. and status>.;
proc phreg data=t2;
     class educ ases;
     model seuraika*status(0) = sex age educ ases kohortti &altiste alttime / rl;
     alttime=&altiste*log(seuraika);
     proportionality_test: test alttime;
     ods output CensoredSummary=cs TestStmts=ph;
     data res; merge cs ph; dgnro=&dg;
     keep dgnro Total Event label WaldChiSq DF ProbChiSq;
     data res; merge res(in=i) Tautiselitteet; by dgnro; if i;
proc append base=&resfile data=res;
run;
%mend;
```

```
** all diseases **;
proc datasets lib=work memtype=data nolist; delete results_all; quit;
%MACRO coxkaikki;
%DO I = 1 %TO 80;
    %coxph(&I,obes2,results_all);
%END;
%MEND coxkaikki;
%coxkaikki;
proc print data=results_all;
     id dgnro;
     var selite Total Event label WaldChiSq DF ProbChiSq;
run;

** log-log plot **;
proc lifetest data=t2 notable plots=(logsurv, lls);
time seuraika*status(0);
strata obes2;
run;

*********************************************************************;
**  Supplement (Associations between obesity-related health outcomes)**;
*********************************************************************;
** predictor=altdg, outcome=vasdg, output file=resfile **;
** data sets: valitut_taudit2 (diseases), hlot2 (persons) **;
*********************************************************************;
data valitut_taudit;
input dgnro jarj;
cards;
2    1    Bacterial infections
80    2    Anaemia
15    3    Diabetes
29    4    Sleep disorders
33    5    Hypertension
35    6    Angina pectoris
36    7    Myocardial infarction
37    8    Pulmonary embolism
38    9    Arrhythmias
39    10    Heart failure
43    11    Cerebral infarction
45    12    Deep vein thrombosis
49    13    Asthma
53    14    Diseases of liver
55    15    Pancreatitis
57    16    Skin infections and excema
60    17    Gout
61    18    Osteoarthritis
63    19    Back pain
66    20    Renal failure
10    21    Kidney Cancer
;
run;
proc sort data=valitut_taudit; by dgnro;
proc sort data=fh_taudit; by dgnro;
data valitut_taudit2;
     merge valitut_taudit(in=i) fh_taudit;
     by dgnro;
     if i;
proc sort data=valitut_taudit2; by tutknro dgnro;
run;
********************************************;
%macro coxtimedep (altdg,vasdg,resfile);
data altiste;
     set valitut_taudit2;
     IF jarj=&altdg;
     altistepvm=min(exslaalkupvm,slaalkupvm);
     keep tutknro altistepvm;
data tauti;
     set valitut_taudit2;
     IF jarj=&vasdg;
     rename exslaalkupvm=extautipvm slaalkupvm=tautipvm;
     keep tutknro exslaalkupvm slaalkupvm;
data t1;
     merge hlot2(in=i) altiste tauti;
     by tutknro;
     if i;
data t2;
     set t1;
     if kohortti=1 then seurloppupvm=mdy(12,31,2016);
     if kohortti=2 then seurloppupvm=mdy(12,31,2012);
     if altistepvm>seurloppupvm then altistepvm=.;
```

```
      if tautipvm>seurloppupvm then tautipvm=.;

      if .<altistepvm<alkupvm then altistepvm=alkupvm;
      altaika=round((altistepvm-alkupvm+1)/365.25,0.01);
      if .<tautipvm<altistepvm then do; altaika=.; tauti_alt=1; end;

      if tautipvm>. then status=1; else status=0;
      seuraika=(min(tautipvm,kuolinpvm,seurloppupvm)-alkupvm+1)/365.25;
      seuraika=round(seuraika,0.01);

      if altaika=. then altaika=seuraika;
      if 0<=altaika<seuraika then altistus=1; else altistus=0;
      if altaika=0 then altistus0=1; else altistus0=0;

      if extautipvm>. then delete;
proc phreg data=t2;
      model seuraika*status(0) = sex age educ ases kohortti timedep_altistus / rl;
      if seuraika<=altaika then timedep_altistus=0; else timedep_altistus=1;
      ods output ParameterEstimates=pe CensoredSummary=cs;
      data pe; set pe; if parameter='timedep_altistus';
      data res; merge pe cs; vjarj=&vasdg; ajarj=&altdg;
      keep parameter vjarj ajarj Total Event HazardRatio HRLowerCL HRUpperCL ProbChiSq;
      data Tautiselitteet2; merge valitut_taudit(in=i) Tautiselitteet; by dgnro; if i;
      proc sort data=Tautiselitteet2; by jarj;
      data vTautiselitteet; set Tautiselitteet2; rename jarj=vjarj dgnro=vastedg selite=vtauti;
      data aTautiselitteet; set Tautiselitteet2; rename jarj=ajarj dgnro=altistedg selite=atauti;
      data res; merge res(in=i) vTautiselitteet; by vjarj; if i;
      data res; merge res(in=i) aTautiselitteet; by ajarj; if i; run;
proc append base=&resfile data=res;
run;
%mend;
** all diseases **;
proc datasets lib=work memtype=data nolist; delete results; quit;
%MACRO coxkaikkiv;
%DO I = 1 %TO 21;
    %DO J = 1 %TO 21;
    %coxtimedep(&I,&J,results);
    %END;
%END;
%MEND coxkaikkiv;
%coxkaikkiv;
proc print data=results; where altistedg NE vastedg;
      id altistedg;
      var atauti vastedg vtauti HazardRatio HRLowerCL HRUpperCL ProbChiSq Total Event;
run;

********************************************************************;
**  Supplement (Count of diseases, Poisson regression)  **;
********************************************************************;
proc freq data=taudit2 noprint;
      tables tutknro / out=tsum1;
data tsum1; set tsum1;
      tautisum=count;
data tsum2;
      merge hlot3(in=i) tsum1;
      by tutknro;
      if i;
data tsum3;
      set tsum2;
      if tautisum=. then tautisum=0;
      lnseuraika=log(maxseura/10000);
run;
proc freq data=tsum3;
      tables bmi4 bmi6;
run;
proc genmod data=tsum3;
      class educ ases BMI4;
      model tautisum = sex age educ ases kohortti BMI4 / dist=poisson offset=lnseuraika;
      lsmeans BMI4 / exp cl;
      estimate 'overw vs normal' BMI4 0 -1 1 0;
      estimate 'obese vs normal' BMI4 0 -1 0 1;
run;
proc genmod data=tsum3;
      class educ ases BMI6;
      model tautisum = sex age educ ases kohortti BMI6 / dist=poisson offset=lnseuraika;
      lsmeans BMI6 / exp cl;
      estimate 'overw vs normal'  BMI6 0 -1 1 0 0 0;
      estimate 'obese1 vs normal' BMI6 0 -1 0 1 0 0;
      estimate 'obese2 vs normal' BMI6 0 -1 0 0 1 0;
      estimate 'obese3 vs normal' BMI6 0 -1 0 0 0 1;
```

```
run;
```

Statistical code (R, version 4.0.0):

```
############ Supplement (pooled cohort-adjusted analysis vs.  ############
############ fixed-effect meta-analysis)                      ############
library(meta)
labels<-c("FPS","HeSSup")
est1<-c  (0.4204,0.44553)
se1<-c   (0.01831,0.03636)
met1<-metagen(est1, se1,sm="HR",labels,comb.fixed=TRUE,comb.random=FALSE)
summary(met1)
forest(met1,leftcols="studlab",print.tau2=TRUE)

############ Supplement (Aalen additive hazard model) ############
library("survival")
library("timereg")
library("haven")
fitAalen<-aalen(Surv(seuraika1,status1==1)~const(bmi4)+
                  const(sex)+const(age)+const(kohortti)+const(educ)+const(ases),data=MORB)
summary(fitAalen)
```

Additional results

Single diseases in Finnish cohorts

Detailed results from disease-specific analyses on obesity, overweight and underweight in relation to 78 health outcomes in Finnish cohorts are shown in supplement tables 4 to 6.

Supplement table 4. Associations of obesity versus normal weight with 78 health outcomes in Finnish cohorts

Disease outcome	N (total)	N (incident cases)	Hazard ratio*	Lower CL	Upper CL	P-value
**Infections**	82078	2413	2.01	1.84	2.19	<.0001
Bacterial infections	82365	2077	2.16	1.97	2.37	<.0001
Viral infections	83069	367	1.13	0.88	1.44	0.3319
**Cancer**	82263	4264	1.08	1.01	1.16	0.0215
Colorectal cancer	83309	375	1.13	0.90	1.42	0.2769
Lung cancer	83351	197	0.59	0.42	0.82	0.0019
Melanoma	83255	898	1.06	0.91	1.23	0.4754
Breast cancer	65566	1883	0.99	0.89	1.10	0.8285
Prostate cancer	17175	310	1.02	0.80	1.29	0.8982
Kidney cancer	83340	97	1.57	1.03	2.39	0.0363
Brain cancer	83347	107	0.94	0.60	1.48	0.7811
Leukaemia, lymphoma	83212	423	1.17	0.95	1.46	0.1482
**Diseases of the blood**	83123	489	1.78	1.47	2.16	<.0001
Anaemia	83246	269	1.69	1.30	2.18	0.0001
**Endocrine diseases**	81702	4795	8.30	7.78	8.86	<.0001
Diabetes	82117	4130	12.14	11.24	13.11	<.0001
**Mental and behavioural disorders**	82384	1754	1.06	0.95	1.19	0.3288
Dementia	83342	345	0.76	0.60	0.97	0.0301
Disorders due to substance abuse	83171	524	0.88	0.71	1.08	0.2165
Psychotic disorders	82736	629	1.03	0.85	1.25	0.7579
Mood disorders	82870	849	1.17	1.00	1.38	0.0564
Neurotic disorders	83143	314	1.10	0.84	1.45	0.4842
**Diseases of the nervous system**	81231	4314	1.85	1.74	1.98	<.0001
Parkinson disease	83311	182	0.66	0.46	0.94	0.0220
Multiple sclerosis	83253	200	0.81	0.55	1.20	0.2983
Epilepsy	82554	440	1.09	0.88	1.36	0.4159
Headaches	83158	301	0.73	0.53	1.00	0.0482
TIA	83264	549	1.28	1.06	1.54	0.0099
Sleep disorders	82854	1926	6.27	5.68	6.92	<.0001
**Diseases of the eye**	82410	3942	1.20	1.12	1.29	<.0001
**Diseases of the ear**	82841	803	1.19	1.01	1.39	0.0359
**Diseases of the circulatory system**	79238	6978	1.47	1.39	1.54	<.0001
Hypertension	78318	4135	3.20	3.00	3.41	<.0001
Ischemic heart diseases	82744	1818	1.39	1.26	1.53	<.0001
Angina pectoris	82948	761	1.54	1.32	1.79	<.0001
Myocardial infarction	83223	606	1.52	1.28	1.80	<.0001
Pulmonary embolism	83273	323	2.86	2.27	3.60	<.0001
Arrhythmias	82747	2945	1.72	1.59	1.86	<.0001
Heart failure	83242	386	4.17	3.35	5.20	<.0001
Cerebrovascular diseases	83137	1013	1.29	1.13	1.47	0.0002
Stroke	83186	847	1.41	1.22	1.63	<.0001
Intracerebral haemorrhage	83336	157	1.40	1.00	1.96	0.0523
Cerebral infarction	83254	562	1.52	1.27	1.81	<.0001
Arteriosclerosis	83321	167	0.79	0.56	1.10	0.1661
Deep vein thrombosis	83156	362	2.43	1.96	3.03	<.0001
**Diseases of the respiratory system**	78671	5188	1.35	1.27	1.44	<.0001
Influenza and Pneumonia	82677	1794	1.45	1.31	1.61	<.0001
Chronic obstructive bronchitis	83298	343	1.06	0.84	1.34	0.6164
Asthma	79690	2636	1.95	1.79	2.12	<.0001
**Diseases of the digestive system**	78567	8244	1.57	1.50	1.65	<.0001
Appendicitis	82516	1047	1.12	0.96	1.30	0.1544
Inflammatory bowel disease	82665	689	1.16	0.97	1.40	0.1036
Diseases of liver	83219	589	1.85	1.55	2.20	<.0001
Alcoholic liver disease	83343	147	1.75	1.24	2.47	0.0015
Pancreatitis	83251	222	1.86	1.40	2.47	<.0001
**Diseases of the skin**	82693	904	2.03	1.76	2.34	<.0001
Skin infections and excema	83022	448	2.49	2.04	3.04	<.0001
**Diseases of the musculoskeletal system**	75613	11446	1.41	1.36	1.47	<.0001
Rheumatoid arthritis and related disorders	82046	1669	1.33	1.19	1.48	<.0001
Gout	83277	170	4.31	3.10	5.99	<.0001
Osteoarthritis	82096	3763	2.71	2.54	2.90	<.0001
Sciatica	82547	1148	1.14	0.99	1.32	0.0637
Back pain	83017	553	1.85	1.54	2.23	<.0001
Soft tissue disorders	81353	3678	1.20	1.11	1.29	<.0001
**Diseases of the genitourinary system**	76832	8604	1.20	1.14	1.26	<.0001
Renal failure	83310	248	2.95	2.27	3.83	<.0001
**Pregnancy complications**	62765	2128	1.35	1.19	1.54	<.0001
Spontaneous abortion	65518	409	1.00	0.74	1.36	0.9952
Hypertension in pregnancy	65684	372	1.76	1.34	2.32	0.0001
Diabetes in pregnancy	65511	379	4.82	3.87	6.00	<.0001
**Miscellaneous**						
Circulatory and respiratory symptoms	82534	1361	1.30	1.16	1.47	<.0001
Digestive and abdominal symptoms	81838	1822	1.36	1.22	1.52	<.0001
Injury	79400	7084	1.07	1.01	1.13	0.0291
Poisoning	83155	531	1.10	0.90	1.34	0.3736
Road accidents	65643	518	0.86	0.70	1.07	0.1711
Falls	64119	2903	1.15	1.06	1.25	0.0012
Self-harm	65867	262	1.19	0.90	1.58	0.2150
**Death**	83358	2064	1.32	1.20	1.45	<.0001

*Adjusted for age, sex, education, cohort and neighbourhood deprivation.

Supplement table 5. Associations of overweight versus normal weight with 78 health outcomes in Finnish cohorts

Disease outcome	N (total)	N (incident cases)	Hazard ratio*	Lower CL	Upper CL	P-value
**Infections**	90557	2453	1.24	1.14	1.35	<.0001
Bacterial infections	90856	2059	1.25	1.14	1.37	<.0001
Viral infections	91474	427	1.12	0.91	1.38	0.2784
**Cancer**	90667	4875	1.04	0.98	1.11	0.1555
Colorectal cancer	91737	438	1.10	0.91	1.34	0.3215
Lung cancer	91771	269	0.96	0.75	1.24	0.7720
Melanoma	91667	1050	1.00	0.88	1.14	0.9468
Breast cancer	68356	2071	1.01	0.92	1.11	0.7911
Prostate cancer	22784	456	1.09	0.91	1.32	0.3473
Kidney cancer	91765	111	1.30	0.88	1.91	0.1855
Brain cancer	91767	115	0.72	0.48	1.09	0.1218
Leukaemia, lymphoma	91641	521	1.25	1.05	1.50	0.0135
**Diseases of the blood**	91568	514	1.25	1.04	1.50	0.0164
Anaemia	91681	289	1.22	0.95	1.55	0.1147
**Endocrine diseases**	90466	3324	2.49	2.32	2.67	<.0001
Diabetes	90890	2556	3.09	2.84	3.37	<.0001
**Mental and behavioural disorders**	90841	1949	0.89	0.81	0.98	0.0194
Dementia	91767	440	0.97	0.80	1.17	0.7338
Disorders due to substance abuse	91567	588	0.69	0.58	0.83	0.0001
Psychotic disorders	91269	690	0.88	0.74	1.04	0.1370
Mood disorders	91349	940	1.00	0.87	1.16	0.9784
Neurotic disorders	91577	339	0.91	0.71	1.16	0.4491
**Diseases of the nervous system**	90032	4617	1.34	1.26	1.42	<.0001
Parkinson disease	91738	242	0.96	0.74	1.25	0.7764
Multiple sclerosis	91677	240	1.25	0.94	1.66	0.1333
Epilepsy	90960	510	1.06	0.88	1.28	0.5351
Headaches	91589	360	1.10	0.87	1.39	0.4278
TIA	91685	634	1.15	0.97	1.35	0.1039
Sleep disorders	91491	1376	1.84	1.65	2.06	<.0001
**Diseases of the eye**	90900	4488	1.09	1.02	1.15	0.0090
**Diseases of the ear**	91225	888	1.03	0.89	1.19	0.7085
**Diseases of the circulatory system**	87811	7782	1.18	1.13	1.24	<.0001
Hypertension	87700	4183	1.74	1.64	1.85	<.0001
Ischemic heart diseases	91218	2155	1.22	1.12	1.33	<.0001
Angina pectoris	91383	890	1.28	1.12	1.47	0.0003
Myocardial infarction	91673	742	1.34	1.16	1.56	0.0001
Pulmonary embolism	91728	272	1.21	0.95	1.56	0.1286
Arrhythmias	91265	3153	1.18	1.10	1.27	<.0001
Heart failure	91675	314	1.70	1.35	2.15	<.0001
Cerebrovascular diseases	91583	1170	1.14	1.01	1.28	0.0368
Stroke	91627	954	1.13	0.99	1.29	0.0721
Intracerebral haemorrhage	91761	163	0.92	0.67	1.28	0.6258
Cerebral infarction	91700	644	1.22	1.04	1.43	0.0165
Arteriosclerosis	91748	205	0.88	0.66	1.17	0.3784
Deep vein thrombosis	91599	337	1.25	1.00	1.57	0.0529
**Diseases of the respiratory system**	86908	5679	1.06	1.00	1.12	0.0505
Influenza and Pneumonia	91084	1862	0.95	0.86	1.05	0.3318
Chronic obstructive bronchitis	91740	385	0.87	0.70	1.07	0.1811
Asthma	88228	2685	1.28	1.18	1.39	<.0001
**Diseases of the digestive system**	87252	9135	1.17	1.12	1.22	<.0001
Appendicitis	90883	1159	0.99	0.87	1.12	0.8340
Inflammatory bowel disease	91035	747	0.92	0.78	1.09	0.3302
Diseases of liver	91656	597	1.13	0.95	1.33	0.1725
Alcoholic liver disease	91759	165	1.20	0.87	1.64	0.2710
Pancreatitis	91711	248	1.39	1.07	1.80	0.0147
**Diseases of the skin**	91168	901	1.22	1.06	1.41	0.0055
Skin infections and excema	91462	444	1.39	1.14	1.70	0.0012
**Diseases of the musculoskeletal system**	84309	12881	1.21	1.17	1.26	<.0001
Rheumatoid arthritis and related disorders	90448	1815	1.13	1.02	1.25	0.0155
Gout	91701	144	1.63	1.16	2.30	0.0050
Osteoarthritis	90862	3738	1.64	1.54	1.75	<.0001
Sciatica	90952	1341	1.15	1.02	1.29	0.0239
Back pain	91463	563	1.19	0.99	1.42	0.0590
Soft tissue disorders	89927	4252	1.14	1.07	1.22	<.0001
**Diseases of the genitourinary system**	85390	9438	1.09	1.04	1.14	0.0002
Renal failure	91728	210	1.20	0.90	1.59	0.2177
**Pregnancy complications**	65726	2263	1.25	1.12	1.39	<.0001
Spontaneous abortion	68336	428	0.93	0.71	1.21	0.5725
Hypertension in pregnancy	68553	396	1.55	1.22	1.96	0.0004
Diabetes in pregnancy	68470	375	2.92	2.36	3.62	<.0001
**Miscellaneous**						
Circulatory and respiratory symptoms	90971	1609	1.25	1.13	1.39	<.0001
Digestive and abdominal symptoms	90307	1934	1.06	0.96	1.18	0.2254
Injury	87672	8342	1.07	1.02	1.12	0.0041
Poisoning	91582	586	0.93	0.78	1.11	0.4331
Road accidents	71310	598	0.94	0.79	1.13	0.5114
Falls	69773	3285	1.08	1.00	1.16	0.0416
Self-harm	71534	301	1.18	0.92	1.50	0.1891
**Death**	91779	2333	1.06	0.98	1.16	0.1529

*Adjusted for age, sex, education, cohort and neighbourhood deprivation.

Supplement table 6. Associations of underweight versus normal weight with 78 health outcomes in Finnish cohorts

Disease outcome	N (total)	N (incident cases)	Hazard ratio*	Lower CL	Upper CL	P-value
**Infections**	62935	1526	1.57	1.19	2.07	0.0013
Bacterial infections	63149	1267	1.52	1.12	2.08	0.0077
Viral infections	63543	286	1.68	0.94	3.02	0.0809
**Cancer**	63083	3097	1.11	0.87	1.43	0.3976
Colorectal cancer	63728	260	1.30	0.53	3.15	0.5648
Lung cancer	63754	153	1.48	0.47	4.66	0.5039
Melanoma	63699	675	1.33	0.80	2.23	0.2748
Breast cancer	51118	1439	0.79	0.53	1.19	0.2625
Prostate cancer	12258	200	2.47	0.61	10.05	0.2057
Kidney cancer	63754	56	NA			
Brain cancer	63752	81	0.58	0.08	4.21	0.5915
Leukaemia, lymphoma	63667	297	0.58	0.18	1.80	0.3420
**Diseases of the blood**	63614	315	1.36	0.70	2.64	0.3717
Anaemia	63688	174	1.78	0.78	4.04	0.1686
**Endocrine diseases**	63063	1398	0.64	0.40	1.02	0.0603
Diabetes	63314	883	0.52	0.26	1.05	0.0665
**Mental and behavioural disorders**	63181	1371	1.60	1.21	2.10	0.0009
Dementia	63748	256	2.19	1.03	4.64	0.0422
Disorders due to substance abuse	63633	404	1.26	0.67	2.37	0.4771
Psychotic disorders	63472	505	1.56	1.02	2.41	0.0425
Mood disorders	63499	663	1.28	0.85	1.94	0.2352
Neurotic disorders	63628	253	1.45	0.79	2.67	0.2334
**Diseases of the nervous system**	62779	2771	1.02	0.79	1.31	0.8876
Parkinson disease	63732	144	1.00	0.25	4.06	0.9980
Multiple sclerosis	63671	173	0.95	0.39	2.32	0.9083
Epilepsy	63162	325	1.70	0.93	3.12	0.0850
Headaches	63638	265	1.95	1.13	3.36	0.0161
TIA	63712	374	1.55	0.80	3.00	0.1989
Sleep disorders	63644	662	0.61	0.29	1.29	0.1975
**Diseases of the eye**	63209	2733	1.02	0.76	1.37	0.8994
**Diseases of the ear**	63422	591	1.17	0.70	1.95	0.5568
**Diseases of the circulatory system**	61308	4692	0.80	0.63	1.00	0.0483
Hypertension	61909	2166	0.44	0.28	0.70	0.0004
Ischemic heart diseases	63520	1136	0.82	0.47	1.41	0.4637
Angina pectoris	63590	454	1.33	0.66	2.69	0.4245
Myocardial infarction	63713	358	1.04	0.43	2.53	0.9246
Pulmonary embolism	63730	159	0.71	0.18	2.87	0.6281
Arrhythmias	63454	1908	0.91	0.64	1.30	0.6184
Heart failure	63692	134	1.09	0.27	4.44	0.8995
Cerebrovascular diseases	63646	671	1.19	0.69	2.07	0.5299
Stroke	63670	538	1.07	0.55	2.07	0.8400
Intracerebral haemorrhage	63748	99	0.61	0.08	4.37	0.6199
Cerebral infarction	63709	341	0.80	0.30	2.14	0.6513
Arteriosclerosis	63743	115	1.43	0.35	5.83	0.6150
Deep vein thrombosis	63650	199	0.71	0.23	2.22	0.5547
**Diseases of the respiratory system**	60530	3887	1.02	0.84	1.24	0.8560
Influenza and Pneumonia	63314	1245	1.48	1.07	2.05	0.0183
Chronic obstructive bronchitis	63737	241	3.94	2.24	6.92	<.0001
Asthma	61402	1748	0.93	0.68	1.28	0.6686
**Diseases of the digestive system**	61010	5805	1.17	0.99	1.38	0.0590
Appendicitis	63151	841	1.15	0.79	1.68	0.4706
Inflammatory bowel disease	63246	540	0.96	0.56	1.64	0.8873
Diseases of liver	63677	364	1.60	0.88	2.93	0.1267
Alcoholic liver disease	63748	89	2.12	0.66	6.78	0.2047
Pancreatitis	63712	139	1.40	0.51	3.81	0.5122
**Diseases of the skin**	63374	581	0.84	0.48	1.46	0.5304
Skin infections and excema	63566	269	0.66	0.27	1.59	0.3511
**Diseases of the musculoskeletal system**	59338	8168	0.78	0.67	0.92	0.0032
Rheumatoid arthritis and related disorders	62855	1220	0.88	0.59	1.30	0.5120
Gout	63733	62	1.25	0.17	9.08	0.8280
Osteoarthritis	63350	1948	0.61	0.40	0.92	0.0196
Sciatica	63276	893	0.97	0.63	1.49	0.8912
Back pain	63558	365	0.98	0.50	1.91	0.9555
Soft tissue disorders	62679	2680	0.83	0.63	1.10	0.2003
**Diseases of the genitourinary system**	59462	6634	1.07	0.92	1.23	0.3979
Renal failure	63722	122	3.39	1.56	7.33	0.0020
**Pregnancy complications**	49202	1925	0.86	0.70	1.06	0.1669
Spontaneous abortion	51025	386	1.33	0.89	1.99	0.1643
Hypertension in pregnancy	51213	318	0.63	0.34	1.15	0.1287
Diabetes in pregnancy	51255	245	0.43	0.19	0.96	0.0405
**Miscellaneous**						
Circulatory and respiratory symptoms	63348	930	0.94	0.58	1.52	0.7923
Digestive and abdominal symptoms	62713	1383	1.59	1.23	2.05	0.0004
Injury	61346	5485	0.99	0.82	1.18	0.8667
Poisoning	63628	401	0.73	0.36	1.47	0.3752
Road accidents	48949	406	0.60	0.25	1.46	0.2633
Falls	48081	2112	1.10	0.81	1.48	0.5535
Self-harm	49096	191	0.66	0.21	2.08	0.4828
**Death**	63758	1342	1.70	1.22	2.39	0.0020

*Adjusted for age, sex, education, cohort and neighbourhood deprivation.

Obesity-related multimorbidity in Finnish cohorts

The associations of BMI with simple and complex multimorbidity did not differ between the two Finnish cohorts, FPS and HeSSup (supplement table 7). Similarly, the results from pooled analysis of individual-level data from these cohorts did not differ from those obtained using an alternative analysis of fixed effect meta-analysis of cohort-specific estimates (supplement table 8).

Supplement table 7. Associations of BMI category with incident obesity-related disease and multimorbidity in FPS and HeSSup cohorts

		HR (95% CI)*			
**BMI category**	**N**	**1st disease**	**2nd disease**	**3rd disease**	**4th disease**
**FPS**					
Normal weight	42732	1.00 (reference)	1.00 (reference)	1.00 (reference)	1.00 (reference)
Overweight	19106	1.52 (1.47-1.58)	1.88 (1.75-2.03)	2.27 (1.92-2.68)	2.83 (1.98-4.05)
Obese	12264	2.87 (2.76-2.98)	5.12 (4.76-5.51)	8.06 (6.92-9.40)	11.85 (8.53-16.46)
**HeSSup**					
Normal weight	13125	1.00 (reference)	1.00 (reference)	1.00 (reference)	1.00 (reference)
Overweight	5389	1.56 (1.45-1.68)	1.92 (1.65-2.24)	2.39 (1.71-3.35)	2.18 (1.07-4.42)
Obese	2603	2.66 (2.45-2.88)	5.39 (4.62-6.30)	8.50 (6.14-11.77)	14.21 (7.58-26.62)

*Adjusted for age, sex, cohort, education, and neighbourhood deprivation.

Supplement table 8. Associations of BMI category with incident obesity-related disease and multimorbidity based on pooled analysis and meta-analysis in Finnish cohorts

		HR (95% CI)*			
**BMI category**	**N**	**1st disease**	**2nd disease**	**3rd disease**	**4th disease**
**Pooled analysis of FPS and HeSSup**					
Normal weight	55857	1.00 (reference)	1.00 (reference)	1.00 (reference)	1.00 (reference)
Overweight	24495	1.53 (1.49-1.58)	1.89 (1.77-2.03)	2.30 (1.98-2.66)	2.67 (1.94-3.68)
Obese	14867	2.83 (2.74-2.93)	5.17 (4.84-5.53)	8.18 (7.12-9.39)	12.39 (9.26-16.58)
**Fixed meta-analysis FPS vs HeSSup**					
Normal weight	55857	1.00 (reference)	1.00 (reference)	1.00 (reference)	1.00 (reference)
Overweight	24495	1.53 (1.48-1.58)	1.89 (1.77-2.02)	2.29 (1.98-2.66)	2.68 (1.95-3.69)
Obese	14867	2.83 (2.73-2.93)	5.17 (4.84-5.52)	8.14 (7.09-9.35)	12.32 (9.21-16.48)

*Adjusted for age, sex, cohort, education, and neighbourhood deprivation.

The associations of BMI with simple and complex multimorbidity and the dose-response association across severity levels of obesity in table 3 were robust in analyses accounting for further baseline characteristics. As shown in supplement table 9, additional adjustments for lifestyle factors had little effect on these associations.

Supplement table 9. Lifestyle-adjusted associations of BMI category with incident obesity-related disease and multimorbidity in Finnish cohorts

		1st disease			2nd disease			3rd disease			4th disease		
**BMI category**	**N (total)**	**N (cases)**	**HR (95% CI)***	**PAF (95% CI)**	**N (cases)**	**HR (95% CI)***	**PAF (95% CI)**	**N (cases)**	**HR (95% CI)***	**PAF (95% CI)**	**N (cases)**	**HR (95% CI)***	**PAF (95% CI)**
**Finnish cohorts**													
Underweight	1476	161	0.79 (0.68-0.93)	-0.22 (-0.36--0.08)	29	0.92 (0.64-1.33)	-0.06 (-0.31-0.26)	8	–	–	<5	–	–
Normal weight	55857	9373	1.00 (reference)	0.00 (reference)	1676	1.00 (reference)	0.00 (reference)	309	1.00 (reference)	0.00 (reference)	62	1.00 (reference)	0.00 (reference)
Overweight	24495	6813	1.53 (1.48-1.58)	9.5 (8.9-10.2)	1764	1.89 (1.76-2.02)	12.2 (11-13.3)	431	2.29 (1.97-2.65)	13.7 (11.5-15.6)	104	2.67 (1.94-3.68)	13.8 (9.8-16.8)
Obese	14867	5672	2.80 (2.70-2.90)	19.6 (19-20.3)	2058	5.07 (4.74-5.42)	33.8 (32.6-35)	645	7.83 (6.81-9.01)	44.1 (41.8-46)	195	11.76 (8.76-15.78)	53.8 (49.3-56.3)
Obese, class 1	12496	4519	2.60 (2.51-2.70)	14.7 (14.1-15.2)	1565	4.54 (4.23-4.87)	24.6 (23.7-25.4)	454	6.55 (5.65-7.60)	29.9 (28.4-31)	127	9.20 (6.74-12.56)	34.4 (31.6-35.6)
Obese, class 2	1839	875	3.77 (3.51-4.04)	3.7 (3.5-4)	358	7.29 (6.49-8.18)	6.4 (5.9-6.9)	136	13.07 (10.64-16.07)	9.6 (8.7-10.4)	53	24.08 (16.53-35.09)	14.2 (12.6-15.4)
Obese, class 3	532	278	4.95 (4.39-5.58)	1.5 (1.4-1.7)	135	12.12 (10.15-14.47)	3.3 (2.9-3.8)	55	23.71 (17.72-31.72)	5.2 (4.4-6.1)	15	31.17 (17.58-55.24)	5.4 (3.9-7.1)

*Adjusted for age, sex, cohort, education, neighbourhood socioeconomic deprivation, smoking, alcohol consumption and physical activity.

Sensitivity analyses using an alternative reference group (supplement table 10) and alternative definitions of obesity-related multimorbidity supported the robustness of our results (supplement table 11). The excess risk of obesity-related multimorbidity was observed when using a more homogeneous reference group with BMI between 22.5 and <25kg/m^2. It was also observed with alternative definitions of multimorbidity, such as having four or more of the 26 specific diseases associated with obesity at a Bonferroni-adjusted significance level irrespective of HR cut-point (including no cut-point), or of having diseases from two or more of the 13 ICD-10 chapters.

Supplement table 10. Associations of BMI category with incident obesity-related disease and multimorbidity using alternative reference group of normal weight participants with BMI 22.5 to <25kg/m^2 in Finnish cohorts

		HR (95% CI)*			
**BMI category**	**N**	**1st disease**	**2nd disease**	**3rd disease**	**4th disease**
**Reference category 22·5–<25·0 kg/m²**					
Normal weight	26623	1.00 (reference)	1.00 (reference)	1.00 (reference)	1.00 (reference)
Overweight	24495	1.39 (1.34-1.44)	1.61 (1.49-1.74)	1.91 (1.62-2.26)	2.48 (1.71-3.60)
Obese	14867	2.56 (2.46-2.66)	4.39 (4.08-4.73)	6.78 (5.79-7.94)	11.54 (8.13-16.38)
Obese, class 1	12496	2.38 (2.28-2.48)	3.93 (3.63-4.25)	5.64 (4.78-6.66)	8.94 (6.20-12.88)
Obese, class 2	1839	3.44 (3.20-3.70)	6.28 (5.57-7.08)	11.31 (9.10-14.06)	24.00 (15.76-36.54)
Obese, class 3	532	4.49 (3.98-5.07)	10.49 (8.76-12.55)	20.83 (15.46-28.08)	31.45 (17.25-57.34)

*Adjusted for age, sex, cohort, education, and neighbourhood deprivation.

Supplement table 11. Associations of BMI category with incident obesity-related disease and multimorbidity using alternative definitions of multimorbidity in Finnish cohorts

		HR (95% CI)*			
**BMI category**	**N**	**1st disease**	**2nd disease**	**3rd disease**	**4th disease**
**All 26 Bonferroni significant obesity-related conditions included in outcomes†**					
Normal weight	52645	1.00 (reference)	1.00 (reference)	1.00 (reference)	1.00 (reference)
Overweight	22773	1.34 (1.30-1.38)	1.57 (1.49-1.65)	1.84 (1.67-2.02)	1.94 (1.63-2.32)
Obese	13442	2.10 (2.03-2.16)	3.24 (3.07-3.42)	4.51 (4.11-4.95)	5.91 (5.01-6.99)
Obese, class 1	11297	1.96 (1.90-2.03)	2.94 (2.78-3.12)	3.93 (3.56-4.35)	4.82 (4.02-5.78)
Obese, class 2	1667	2.76 (2.58-2.95)	4.51 (4.08-5.00)	6.90 (5.89-8.09)	10.91 (8.51-13.98)
Obese, class 3	478	3.36 (2.98-3.79)	6.57 (5.54-7.78)	10.53 (8.19-13.53)	14.24 (9.43-21.52)
**All 13 obesity-related disease categories (ICD-chapters) included in outcomes‡**					
Normal weight	45543	1.00 (reference)	1.00 (reference)	1.00 (reference)	1.00 (reference)
Overweight	19817	1.20 (1.17-1.23)	1.28 (1.23-1.34)	1.43 (1.33-1.53)	1.47 (1.30-1.66)
Obese	11477	1.53 (1.49-1.57)	2.01 (1.93-2.10)	2.54 (2.37-2.73)	3.34 (2.96-3.77)
Obese, class 1	9507	1.46 (1.41-1.5)	1.87 (1.79-1.96)	2.33 (2.16-2.52)	2.89 (2.53-3.30)
Obese, class 2	1524	1.86 (1.75-1.98)	2.61 (2.39-2.85)	3.38 (2.96-3.87)	5.23 (4.28-6.39)
Obese, class 3	446	2.13 (1.90-2.39)	3.02 (2.59-3.52)	3.98 (3.16-5.01)	5.90 (4.15-8.38)

*Adjusted for age, sex, cohort, education, and neighbourhood deprivation.

†Diabetes, hypertension, angina pectoris, heart failure, myocardial infarction, arrhythmias, deep vein thrombosis, pulmonary embolism, cerebral infarction, anaemia, asthma, sleep disorders, back pain, osteoarthritis, gout, bacterial infections, skin infections and eczema, liver disease, renal failure, pancreatitis, influenza & pneumonia, diseases of the eye, rheumatoid arthritis and related disorders, soft tissue disorders, circulatory & respiratory symptoms, and digestive & abdominal symptoms.

‡Endocrine diseases, infections, cancers, diseases of the blood, eye, ear and skin, and diseases of the circulatory, digestive, genitourinary, musculoskeletal, respiratory and nervous systems.

We observed no sex differences in associations of BMI with simple and complex multimorbidity (P = 0.91, supplement table 12). In contrast, these associations were stronger (p = 0.009) in participants who were younger than 50 at the time of BMI assessment than those aged 50 or older (supplement table 13).

Supplement table 12. Associations of BMI category with incident obesity-related disease and multimorbidity by sex in Finnish cohorts

		HR (95% CI)*			
BMI category	N	1st disease	2nd disease	3rd disease	4th disease
**Men**					
Normal weight	10787	1.00 (reference)	1.00 (reference)	1.00 (reference)	1.00 (reference)
Overweight	8789	1.57 (1.48-1.66)	1.75 (1.56-1.97)	1.74 (1.38-2.20)	2.48 (1.51-4.07)
Obese	3363	2.82 (2.63-3.02)	4.68 (4.14-5.29)	6.35 (5.04-7.99)	10.67 (6.58-17.29)
**Women**					
Normal weight	45070	1.00 (reference)	1.00 (reference)	1.00 (reference)	1.00 (reference)
Overweight	15706	1.52 (1.46-1.58)	1.97 (1.80-2.14)	2.74 (2.26-3.32)	2.74 (1.79-4.18)
Obese	11504	2.84 (2.73-2.95)	5.39 (4.98-5.83)	9.33 (7.85-11.09)	13.53 (9.4-19.47)
P for sex interaction		0.1546	0.3775	0.0806	0.9102

*Adjusted for age, sex, cohort, education, and neighbourhood deprivation.

Supplement table 13. Associations of BMI category with incident obesity-related disease and multimorbidity by age group in Finnish cohorts

		HR (95% CI)*			
BMI category	N	1st disease	2nd disease	3rd disease	4th disease
**BMI at age <50**					
Normal weight	43266	1.00 (reference)	1.00 (reference)	1.00 (reference)	1.00 (reference)
Overweight	15726	1.58 (1.51-1.65)	2.03 (1.83-2.24)	2.78 (2.19-3.52)	4.19 (2.46-7.13)
Obese	9422	3.05 (2.91-3.19)	6.48 (5.91-7.11)	12.20 (9.88-15.06)	22.11 (13.68-35.74)
**BMI at age >=50**					
Normal weight	12591	1.00 (reference)	1.00 (reference)	1.00 (reference)	1.00 (reference)
Overweight	8769	1.46 (1.40-1.54)	1.72 (1.57-1.89)	1.92 (1.59-2.32)	1.90 (1.28-2.83)
Obese	5445	2.56 (2.43-2.70)	4.11 (3.74-4.51)	5.84 (4.86-7.00)	7.90 (5.48-11.41)
P for age interaction		<0.0001	<0.0001	<0.0001	0.0091

*Adjusted for age, sex, cohort, education, and neighbourhood deprivation.

Temporal associations between pairs of obesity-related diseases pairs are shown in supplement figure 3 and related statistics in supplement table 14. The 21 obesity-related diseases were highly inter-connected such that having one disease increased the risk of developing another disease and many associations were bidirectional. Among the first four diseases in obese participants, there were 140 different disease combinations, each of low prevalence (6.2% or less, N<12, supplement table 13). The obesity-related diseases that occurred most frequently were diabetes (75.4%, N=147), hypertension (671.8%, N=140), sleep disorders (42.6%, N=83), osteoarthritis (42.1%, N=82), arrhythmias (34.4%, N=67), bacterial infections (31.3%, N=61) and asthma (22.1%, N=43)

Supplement figure 3. Temporal associations between obesity-related disease pairs in Finnish cohorts

Outcome

**Disease category**	**Disease**	Bacterial infections	Anemia	Diabetes	Sleep disorders	Hypertension	Angina pectoris	Myocardial infarction	Pulmonary embolism	Arrhythmias	Heart failure	Cerebral infarction	Deep vein thrombosis	Asthma	Diseases of liver	Pancreatitis	Skin infections and eczema	Gout	Osteoarthritis	Back pain	Renal failure	Kidney cancer
Infections	Bacterial infections	–	3.7	1.2	1.9	1.0	1.7	2.1	2.4	1.5	2.8	1.8	1.9	1.5	2.0	2.2	5.2	2.7	1.0	1.8	3.7	1.6
Diseases of the blood	Anemia	4.6	–	0.9	0.7	0.8	1.6	1.0	–	1.7	5.1	–	3.1	1.8	16.6	–	3.0	7.9	1.1	–	3.4	–
Endocrine diseases	Diabetes	1.7	2.3	–	1.7	1.5	2.1	1.4	0.9	1.1	1.9	1.3	0.7	1.0	3.0	1.0	2.5	2.6	1.0	1.3	4.4	1.3
Diseases of the nervous system	Sleep disorders	1.8	2.1	1.7	–	1.6	1.5	1.1	1.4	1.6	2.1	1.4	1.3	2.6	1.9	1.3	1.5	2.4	1.1	1.6	3.2	1.1
Diseases of the circulatory system	Hypertension	1.6	1.7	2.1	1.4	–	1.8	1.7	1.1	1.4	1.6	1.6	1.0	1.2	1.8	1.2	1.9	2.8	1.2	1.2	5.1	1.9
	Angina pectoris	1.9	0.7	1.7	0.8	1.3	–	5.2	0.8	1.7	2.6	2.5	0.5	1.0	1.1	1.4	2.7	1.7	0.7	3.0	2.9	1.7
	Myocardial infarction	2.0	1.7	1.7	1.2	1.2	12.3	–	0.9	3.0	4.6	3.5	–	1.1	1.9	0.9	3.2	2.6	0.7	2.3	4.6	–
	Pulmonary embolism	2.9	2.5	1.4	2.5	0.8	0.4	1.1	–	2.6	1.8	3.9	6.4	1.2	0.6	2.0	–	–	1.2	4.8	3.6	–
	Arrhythmias	2.0	2.3	1.3	1.7	1.4	1.8	1.4	1.1	–	7.8	2.4	0.7	1.3	1.7	2.5	2.2	2.4	1.2	1.9	2.6	0.4
	Heart failure	4.3	3.1	2.1	3.5	1.3	3.4	3.1	2.5	7.2	–	2.9	1.6	2.0	1.7	4.2	6.5	9.0	0.8	2.3	16.1	4.0
	Cerebral infarction	2.2	2.1	1.4	2.6	2.1	1.6	2.2	0.7	2.7	2.1	–	1.7	0.9	1.4	1.4	3.0	1.6	0.7	4.9	5.0	2.0
	Deep vein thrombosis	1.9	2.7	0.9	1.5	0.9	0.6	–	6.4	1.4	1.4	2.1	–	0.9	1.7	1.3	0.6	0.9	1.4	1.8	3.3	2.4
Diseases of the respiratory system	Asthma	1.7	0.8	1.2	1.7	1.1	1.2	0.9	1.0	1.2	2.0	1.4	1.4	–	1.1	0.7	1.3	2.2	1.3	2.3	2.1	0.6
Diseases of the digestive system	Diseases of liver	5.9	5.9	1.6	1.3	1.1	2.1	1.0	3.4	1.1	2.6	1.6	1.0	2.0	–	5.8	2.7	1.1	0.8	0.9	5.3	–
	Pancreatitis	2.4	–	1.7	1.0	0.6	1.9	0.7	1.2	1.0	0.7	1.6	1.4	0.7	6.2	–	1.2	1.4	1.8	1.2	3.7	–
Diseases of the skin	Skin infections and eczema	2.8	2.0	1.3	1.7	1.0	1.0	2.0	1.2	1.2	3.9	1.4	–	0.7	2.0	1.1	–	2.6	1.1	1.5	4.4	–
Diseases of the musculoskeletal system	Gout	2.4	5.4	2.1	1.2	1.7	3.1	1.7	1.8	1.9	4.4	1.4	8.9	1.4	2.3	1.6	0.9	–	1.7	1.0	9.0	2.8
	Osteoarthritis	1.1	1.4	1.1	1.3	1.1	1.3	1.4	1.1	1.1	1.3	1.0	1.6	1.3	1.2	1.1	1.6	1.7	–	1.7	1.8	1.1
	Back pain	2.4	2.4	1.0	0.9	0.8	1.7	1.3	1.0	1.7	2.2	2.5	1.6	1.4	1.4	2.0	1.4	0.7	1.6	–	1.8	2.1
Diseases of the genitourinary system	Renal failure	11.6	14.4	1.1	3.8	2.7	4.7	5.7	2.0	2.2	4.8	3.4	8.9	3.4	9.7	4.1	12.0	8.0	1.1	–	–	7.0
Cancers	Kidney cancer	1.3	–	1.2	1.1	0.7	–	–	15.0	0.7	1.3	1.7	3.8	–	–	–	–	8.7	0.3	–	4.9	–

Hazard ratio

4
3.5
3
2.5
2
1.5
<1.1

Supplementary table 14. Age-, sex-, cohort-, education- and neighbourhood deprivation-adjusted associations between obesity-related disease pairs in Finnish cohorts

Predictor	Outcome	HazardRatio	HRLowerCL	HRUpperCL	ProbChiSq	Total	Incident cases
Anemia	Diseases of liver	16.64	9.46	29.28	<.0001	21176	236
Heart failure	Renal failure	16.08	10.09	25.65	<.0001	21227	133
Kidney cancer	Pulmonary embolism	15.01	6.09	36.99	<.0001	21179	166
Renal failure	Anemia	14.37	5.20	39.74	<.0001	21193	101
Myocardial infarction	Angina pectoris	12.30	8.53	17.72	<.0001	20996	315
Renal failure	Infections and excema	12.01	4.88	29.57	<.0001	21090	184
Renal failure	Bacterial infections	11.60	7.61	17.68	<.0001	20838	852
Renal failure	Diseases of liver	9.70	4.52	20.82	<.0001	21176	236
Heart failure	Gout	9.03	4.88	16.69	<.0001	21183	109
Gout	Renal failure	9.00	4.87	16.65	<.0001	21227	133
Gout	Deep vein thrombosis	8.87	4.05	19.42	<.0001	21142	166
Renal failure	Deep vein thrombosis	8.86	2.80	28.02	0.0002	21142	166
Kidney cancer	Gout	8.66	2.71	27.66	0.0003	21183	109
Renal failure	Gout	8.05	2.91	22.23	0.0001	21183	109
Anemia	Gout	7.94	2.50	25.21	0.0004	21183	109
Arrhythmias	Heart failure	7.78	5.84	10.38	<.0001	21187	254
Heart failure	Arrhythmias	7.16	5.31	9.65	<.0001	20923	1068
Renal failure	Kidney cancer	7.01	0.95	51.88	0.0567	21225	41
Heart failure	Infections and excema	6.50	3.29	12.86	<.0001	21090	184
Deep vein thrombosis	Pulmonary embolism	6.38	3.35	12.13	<.0001	21179	166
Pulmonary embolism	Deep vein thrombosis	6.37	2.81	14.47	<.0001	21142	166
Pancreatitis	Diseases of liver	6.22	2.93	13.22	<.0001	21176	236
Diseases of liver	Anemia	5.93	2.17	16.22	0.0005	21193	101
Diseases of liver	Bacterial infections	5.88	4.05	8.53	<.0001	20838	852
Diseases of liver	Pancreatitis	5.81	1.83	18.49	0.0029	21176	87
Renal failure	Myocardial infarction	5.65	2.32	13.79	0.0001	21148	253
Gout	Anemia	5.40	1.68	17.42	0.0047	21193	101
Diseases of liver	Renal failure	5.29	2.32	12.05	0.0001	21227	133
Bacterial infections	Infections and excema	5.21	3.51	7.75	<.0001	21090	184
Angina pectoris	Myocardial infarction	5.20	3.60	7.53	<.0001	21148	253
Anemia	Heart failure	5.07	2.25	11.44	0.0001	21187	254
Hypertension	Renal failure	5.05	3.44	7.43	<.0001	21227	133
Cerebral infarction	Renal failure	4.99	2.42	10.32	<.0001	21227	133
Cerebral infarction	Back pain	4.87	2.14	11.09	0.0002	21090	197
Kidney cancer	Renal failure	4.86	1.19	19.81	0.0274	21227	133
Renal failure	Heart failure	4.85	1.98	11.84	0.0005	21187	254
Pulmonary embolism	Back pain	4.84	1.98	11.83	0.0005	21090	197
Renal failure	Angina pectoris	4.75	1.95	11.57	0.0006	20996	315
Anemia	Bacterial infections	4.63	2.73	7.86	<.0001	20838	852
Myocardial infarction	Heart failure	4.59	2.87	7.34	<.0001	21187	254
Myocardial infarction	Renal failure	4.55	2.42	8.56	<.0001	21227	133
Infections and excema	Renal failure	4.44	1.96	10.10	0.0004	21227	133
Gout	Heart failure	4.42	2.38	8.18	<.0001	21187	254
Diabetes	Renal failure	4.41	3.07	6.34	<.0001	21227	133
Heart failure	Bacterial infections	4.33	3.01	6.23	<.0001	20838	852
Heart failure	Pancreatitis	4.18	1.30	13.39	0.0163	21176	87
Renal failure	Pancreatitis	4.14	0.57	30.09	0.1604	21176	87
Heart failure	Kidney cancer	4.03	0.96	16.98	0.0575	21225	41
Pulmonary embolism	Cerebral infarction	3.90	1.83	8.31	0.0004	21183	225
Infections and excema	Heart failure	3.87	1.99	7.54	0.0001	21187	254
Renal failure	Sleep disorders	3.81	2.16	6.74	<.0001	20845	1271
Kidney cancer	Deep vein thrombosis	3.81	0.53	27.35	0.1835	21142	166
Pancreatitis	Renal failure	3.74	1.19	11.76	0.0243	21227	133
Bacterial infections	Renal failure	3.72	2.34	5.91	<.0001	21227	133
Bacterial infections	Anemia	3.66	2.07	6.47	<.0001	21193	101
Pulmonary embolism	Renal failure	3.56	1.31	9.68	0.0127	21227	133
Myocardial infarction	Cerebral infarction	3.48	1.97	6.16	<.0001	21183	225
Heart failure	Sleep disorders	3.48	2.45	4.93	<.0001	20845	1271
Heart failure	Angina pectoris	3.44	1.88	6.32	0.0001	20996	315
Renal failure	Asthma	3.44	1.54	7.69	0.0026	19851	929
Anemia	Renal failure	3.44	0.85	13.95	0.0841	21227	133
Diseases of liver	Pulmonary embolism	3.43	1.27	9.29	0.0153	21179	166
Renal failure	Cerebral infarction	3.38	1.08	10.64	0.0373	21183	225
Deep vein thrombosis	Renal failure	3.32	1.36	8.14	0.0087	21227	133
Myocardial infarction	Infections and excema	3.22	1.41	7.37	0.0057	21090	184
Sleep disorders	Renal failure	3.17	2.04	4.91	<.0001	21227	133
Heart failure	Myocardial infarction	3.13	1.60	6.13	0.0009	21148	253
Gout	Angina pectoris	3.11	1.59	6.09	0.0009	20996	315
Anemia	Deep vein thrombosis	3.08	0.76	12.44	0.1147	21142	166
Heart failure	Anemia	3.05	0.96	9.74	0.0597	21193	101
Cerebral infarction	Infections and excema	3.02	1.11	8.20	0.0300	21090	184
Myocardial infarction	Arrhythmias	3.01	2.22	4.07	<.0001	20923	1068
Angina pectoris	Back pain	2.98	1.55	5.74	0.0011	21090	197
Anemia	Infections and excema	2.96	0.73	11.98	0.1273	21090	184
Diabetes	Diseases of liver	2.96	2.21	3.96	<.0001	21176	236
Pulmonary embolism	Bacterial infections	2.92	1.78	4.79	<.0001	20838	852
Angina pectoris	Renal failure	2.91	1.67	5.08	0.0002	21227	133
Heart failure	Cerebral infarction	2.87	1.41	5.85	0.0037	21183	225
Bacterial infections	Heart failure	2.84	1.96	4.13	<.0001	21187	254
Hypertension	Gout	2.83	1.90	4.20	<.0001	21183	109
Infections and excema	Bacterial infections	2.80	1.81	4.32	<.0001	20838	852
Gout	Kidney cancer	2.76	0.37	20.46	0.3214	21225	41
Diseases of liver	Infections and excema	2.74	0.87	8.59	0.0846	21090	184
Deep vein thrombosis	Anemia	2.72	0.86	8.62	0.0886	21193	101

Angina pectoris	Infections and excema	2.71	1.41	5.23	0.0029	21090	184
Cerebral infarction	Arrhythmias	2.70	1.83	3.96	<.0001	20923	1068
Bacterial infections	Gout	2.69	1.50	4.83	0.0009	21183	109
Renal failure	Hypertension	2.69	1.12	6.47	0.0271	18018	1988
Diseases of liver	Heart failure	2.65	1.17	5.97	0.0191	21187	254
Pulmonary embolism	Arrhythmias	2.64	1.71	4.07	<.0001	20923	1068
Infections and excema	Gout	2.64	0.84	8.33	0.0984	21183	109
Arrhythmias	Renal failure	2.61	1.60	4.26	0.0001	21227	133
Myocardial infarction	Gout	2.60	1.13	6.00	0.0252	21183	109
Diabetes	Gout	2.60	1.72	3.93	<.0001	21183	109
Angina pectoris	Heart failure	2.58	1.68	3.95	<.0001	21187	254
Cerebral infarction	Sleep disorders	2.58	1.72	3.87	<.0001	20845	1271
Sleep disorders	Asthma	2.56	2.02	3.27	<.0001	19851	929
Pulmonary embolism	Sleep disorders	2.55	1.64	3.97	<.0001	20845	1271
Arrhythmias	Pancreatitis	2.55	1.21	5.38	0.0143	21176	87
Angina pectoris	Cerebral infarction	2.53	1.58	4.06	0.0001	21183	225
Pulmonary embolism	Anemia	2.51	0.62	10.23	0.1985	21193	101
Diabetes	Infections and excema	2.49	1.74	3.56	<.0001	21090	184
Back pain	Cerebral infarction	2.49	1.17	5.29	0.0176	21183	225
Heart failure	Pulmonary embolism	2.48	0.91	6.76	0.0747	21179	166
Arrhythmias	Cerebral infarction	2.45	1.62	3.70	<.0001	21183	225
Bacterial infections	Pulmonary embolism	2.44	1.45	4.10	0.0008	21179	166
Pancreatitis	Bacterial infections	2.43	1.30	4.54	0.0052	20838	852
Arrhythmias	Gout	2.43	1.39	4.26	0.0019	21183	109
Back pain	Anemia	2.42	0.77	7.66	0.1312	21193	101
Deep vein thrombosis	Kidney cancer	2.42	0.33	17.69	0.3835	21225	41
Back pain	Bacterial infections	2.40	1.59	3.64	<.0001	20838	852
Sleep disorders	Gout	2.40	1.44	4.00	0.0008	21183	109
Gout	Bacterial infections	2.38	1.40	4.06	0.0014	20838	852
Asthma	Back pain	2.32	1.60	3.36	<.0001	21090	197
Diabetes	Anemia	2.32	1.46	3.67	0.0003	21193	101
Gout	Diseases of liver	2.32	0.85	6.29	0.0994	21176	236
Myocardial infarction	Back pain	2.31	0.85	6.29	0.1014	21090	197
Arrhythmias	Anemia	2.26	1.12	4.56	0.0227	21193	101
Heart failure	Back pain	2.26	0.72	7.12	0.1644	21090	197
Asthma	Gout	2.25	1.33	3.78	0.0024	21183	109
Bacterial infections	Pancreatitis	2.24	1.03	4.88	0.0420	21176	87
Cerebral infarction	Bacterial infections	2.23	1.36	3.67	0.0016	20838	852
Arrhythmias	Infections and excema	2.23	1.25	3.97	0.0066	21090	184
Back pain	Heart failure	2.21	1.04	4.69	0.0385	21187	254
Cerebral infarction	Myocardial infarction	2.20	0.98	4.98	0.0575	21148	253
Renal failure	Arrhythmias	2.20	1.10	4.42	0.0268	20923	1068
Cerebral infarction	Heart failure	2.15	1.01	4.58	0.0476	21187	254
Back pain	Kidney cancer	2.15	0.30	15.68	0.4504	21225	41
Sleep disorders	Heart failure	2.14	1.47	3.12	0.0001	21187	254
Cerebral infarction	Anemia	2.14	0.52	8.75	0.2904	21193	101
Heart failure	Diabetes	2.14	1.61	2.83	<.0001	20434	3255
Gout	Diabetes	2.14	1.53	2.98	<.0001	20434	3255
Diseases of liver	Angina pectoris	2.12	0.87	5.14	0.0969	20996	315
Cerebral infarction	Hypertension	2.11	1.31	3.41	0.0022	18018	1988
Asthma	Renal failure	2.11	1.33	3.35	0.0015	21227	133
Sleep disorders	Anemia	2.10	1.05	4.23	0.0371	21193	101
Diabetes	Angina pectoris	2.08	1.60	2.71	<.0001	20996	315
Bacterial infections	Myocardial infarction	2.07	1.33	3.22	0.0012	21148	253
Deep vein thrombosis	Cerebral infarction	2.06	0.85	5.02	0.1103	21183	225
Hypertension	Diabetes	2.06	1.91	2.22	<.0001	20434	3255
Infections and excema	Diseases of liver	2.05	0.84	4.97	0.1139	21176	236
Bacterial infections	Diseases of liver	2.04	1.27	3.28	0.0030	21176	236
Arrhythmias	Bacterial infections	2.03	1.57	2.62	<.0001	20838	852
Myocardial infarction	Bacterial infections	2.03	1.31	3.14	0.0016	20838	852
Back pain	Pancreatitis	2.03	0.50	8.24	0.3246	21176	87
Infections and excema	Myocardial infarction	2.02	0.83	4.90	0.1200	21148	253
Infections and excema	Anemia	2.00	0.49	8.13	0.3313	21193	101
Cerebral infarction	Kidney cancer	2.00	0.27	14.78	0.4959	21225	41
Asthma	Heart failure	1.99	1.41	2.80	0.0001	21187	254
Pulmonary embolism	Pancreatitis	1.98	0.28	14.28	0.4973	21176	87
Heart failure	Asthma	1.97	1.05	3.69	0.0339	19851	929
Renal failure	Pulmonary embolism	1.96	0.27	14.07	0.5036	21179	166
Diseases of liver	Asthma	1.95	1.05	3.64	0.0356	19851	929
Hypertension	Infections and excema	1.94	1.41	2.68	0.0001	21090	184
Arrhythmias	Back pain	1.93	1.04	3.60	0.0380	21090	197
Myocardial infarction	Diseases of liver	1.93	0.85	4.38	0.1157	21176	236
Angina pectoris	Bacterial infections	1.93	1.40	2.64	0.0001	20838	852
Hypertension	Kidney cancer	1.91	1.02	3.57	0.0441	21225	41
Bacterial infections	Deep vein thrombosis	1.90	1.03	3.52	0.0403	21142	166
Diabetes	Heart failure	1.90	1.44	2.52	<.0001	21187	254
Bacterial infections	Sleep disorders	1.89	1.52	2.36	<.0001	20845	1271
Gout	Arrhythmias	1.89	1.15	3.12	0.0120	20923	1068
Deep vein thrombosis	Bacterial infections	1.88	1.13	3.14	0.0155	20838	852
Sleep disorders	Diseases of liver	1.87	1.21	2.91	0.0053	21176	236
Pancreatitis	Angina pectoris	1.86	0.60	5.81	0.2848	20996	315
Deep vein thrombosis	Back pain	1.85	0.59	5.80	0.2919	21090	197
Sleep disorders	Bacterial infections	1.84	1.44	2.35	<.0001	20838	852
Bacterial infections	Back pain	1.83	1.02	3.30	0.0435	21090	197
Osteoarthritis	Renal failure	1.80	1.19	2.74	0.0057	21227	133
Gout	Pulmonary embolism	1.80	0.44	7.34	0.4118	21179	166
Hypertension	Diseases of liver	1.80	1.37	2.37	<.0001	21176	236

Anemia	Asthma	1.79	0.80	4.00	0.1555	19851	929
Arrhythmias	Angina pectoris	1.78	1.19	2.67	0.0049	20996	315
Hypertension	Angina pectoris	1.78	1.41	2.24	<.0001	20996	315
Bacterial infections	Cerebral infarction	1.78	1.10	2.89	0.0200	21183	225
Pulmonary embolism	Heart failure	1.77	0.66	4.77	0.2581	21187	254
Back pain	Renal failure	1.77	0.56	5.56	0.3290	21227	133
Pancreatitis	Osteoarthritis	1.76	1.04	2.97	0.0360	20383	1837
Pancreatitis	Diabetes	1.75	1.18	2.59	0.0054	20434	3255
Heart failure	Diseases of liver	1.75	0.65	4.72	0.2720	21176	236
Hypertension	Anemia	1.74	1.15	2.64	0.0087	21193	101
Gout	Osteoarthritis	1.74	1.04	2.90	0.0342	20383	1837
Anemia	Arrhythmias	1.73	0.86	3.48	0.1218	20923	1068
Gout	Myocardial infarction	1.73	0.71	4.22	0.2288	21148	253
Bacterial infections	Angina pectoris	1.73	1.12	2.67	0.0141	20996	315
Infections and excema	Sleep disorders	1.72	1.14	2.60	0.0098	20845	1271
Diabetes	Sleep disorders	1.72	1.49	1.98	<.0001	20845	1271
Asthma	Sleep disorders	1.72	1.46	2.03	<.0001	20845	1271
Cerebral infarction	Deep vein thrombosis	1.72	0.42	6.98	0.4481	21142	166
Myocardial infarction	Anemia	1.72	0.42	7.05	0.4532	21193	101
Arrhythmias	Diseases of liver	1.72	1.04	2.84	0.0359	21176	236
Deep vein thrombosis	Diseases of liver	1.71	0.64	4.60	0.2893	21176	236
Arrhythmias	Sleep disorders	1.71	1.36	2.14	<.0001	20845	1271
Angina pectoris	Arrhythmias	1.71	1.30	2.23	0.0001	20923	1068
Myocardial infarction	Diabetes	1.70	1.31	2.21	0.0001	20434	3255
Osteoarthritis	Gout	1.70	1.04	2.77	0.0341	21183	109
Angina pectoris	Gout	1.70	0.81	3.55	0.1599	21183	109
Angina pectoris	Diabetes	1.70	1.40	2.05	<.0001	20434	3255
Kidney cancer	Cerebral infarction	1.69	0.24	12.11	0.6007	21183	225
Osteoarthritis	Back pain	1.69	1.10	2.60	0.0166	21090	197
Hypertension	Myocardial infarction	1.69	1.30	2.19	0.0001	21148	253
Angina pectoris	Kidney cancer	1.69	0.51	5.63	0.3955	21225	41
Back pain	Angina pectoris	1.68	0.75	3.76	0.2100	20996	315
Gout	Hypertension	1.68	0.87	3.23	0.1233	18018	1988
Back pain	Arrhythmias	1.68	1.09	2.58	0.0194	20923	1068
Asthma	Bacterial infections	1.66	1.36	2.03	<.0001	20838	852
Diabetes	Bacterial infections	1.66	1.39	1.97	<.0001	20838	852
Sleep disorders	Diabetes	1.65	1.45	1.89	<.0001	20434	3255
Gout	Pancreatitis	1.64	0.23	11.92	0.6250	21176	87
Back pain	Osteoarthritis	1.64	1.15	2.32	0.0058	20383	1837
Hypertension	Heart failure	1.63	1.27	2.11	0.0002	21187	254
Heart failure	Deep vein thrombosis	1.63	0.40	6.61	0.4963	21142	166
Cerebral infarction	Angina pectoris	1.62	0.67	3.94	0.2845	20996	315
Bacterial infections	Kidney cancer	1.62	0.50	5.28	0.4247	21225	41
Back pain	Deep vein thrombosis	1.62	0.52	5.07	0.4099	21142	166
Diseases of liver	Diabetes	1.59	1.10	2.31	0.0142	20434	3255
Osteoarthritis	Infections and excema	1.59	1.03	2.47	0.0371	21090	184
Osteoarthritis	Deep vein thrombosis	1.59	1.02	2.48	0.0419	21142	166
Sleep disorders	Hypertension	1.59	1.30	1.94	<.0001	18018	1988
Sleep disorders	Back pain	1.58	0.87	2.87	0.1324	21090	197
Diseases of liver	Cerebral infarction	1.58	0.51	4.95	0.4318	21183	225
Pancreatitis	Cerebral infarction	1.58	0.39	6.36	0.5206	21183	225
Hypertension	Bacterial infections	1.56	1.35	1.81	<.0001	20838	852
Cerebral infarction	Gout	1.56	0.38	6.38	0.5341	21183	109
Anemia	Angina pectoris	1.56	0.39	6.28	0.5315	20996	315
Sleep disorders	Arrhythmias	1.55	1.25	1.92	0.0001	20923	1068
Hypertension	Cerebral infarction	1.55	1.18	2.04	0.0016	21183	225
Sleep disorders	Angina pectoris	1.55	1.05	2.27	0.0262	20996	315
Infections and excema	Back pain	1.54	0.49	4.83	0.4583	21090	197
Deep vein thrombosis	Sleep disorders	1.53	0.96	2.44	0.0727	20845	1271
Bacterial infections	Arrhythmias	1.52	1.19	1.93	0.0007	20923	1068
Diabetes	Hypertension	1.48	1.27	1.73	<.0001	18018	1988
Bacterial infections	Asthma	1.47	1.09	1.99	0.0118	19851	929
Sleep disorders	Infections and excema	1.46	0.81	2.66	0.2108	21090	184
Gout	Asthma	1.45	0.60	3.50	0.4108	19851	929
Arrhythmias	Hypertension	1.45	1.15	1.82	0.0019	18018	1988
Pulmonary embolism	Diabetes	1.44	1.02	2.05	0.0389	20434	3255
Asthma	Cerebral infarction	1.44	0.96	2.16	0.0745	21183	225
Back pain	Infections and excema	1.44	0.46	4.50	0.5333	21090	184
Back pain	Diseases of liver	1.43	0.53	3.86	0.4746	21176	236
Cerebral infarction	Diseases of liver	1.43	0.46	4.49	0.5393	21176	236
Sleep disorders	Pulmonary embolism	1.43	0.79	2.60	0.2434	21179	166
Hypertension	Sleep disorders	1.42	1.26	1.61	<.0001	20845	1271
Cerebral infarction	Pancreatitis	1.42	0.20	10.29	0.7287	21176	87
Back pain	Asthma	1.42	0.84	2.40	0.1959	19851	929
Deep vein thrombosis	Arrhythmias	1.41	0.88	2.28	0.1573	20923	1068
Sleep disorders	Cerebral infarction	1.41	0.87	2.28	0.1595	21183	225
Deep vein thrombosis	Osteoarthritis	1.41	0.92	2.14	0.1124	20383	1837
Pancreatitis	Gout	1.40	0.20	10.08	0.7368	21183	109
Asthma	Deep vein thrombosis	1.38	0.86	2.23	0.1859	21142	166
Deep vein thrombosis	Heart failure	1.38	0.51	3.72	0.5224	21187	254
Diabetes	Myocardial infarction	1.38	1.01	1.88	0.0435	21148	253
Arrhythmias	Myocardial infarction	1.38	0.84	2.24	0.2003	21148	253
Pancreatitis	Deep vein thrombosis	1.38	0.19	9.84	0.7503	21142	166
Hypertension	Arrhythmias	1.37	1.20	1.55	<.0001	20923	1068
Angina pectoris	Pancreatitis	1.36	0.42	4.40	0.6034	21176	87
Gout	Cerebral infarction	1.36	0.43	4.29	0.5966	21183	225
Cerebral infarction	Diabetes	1.36	0.96	1.92	0.0798	20434	3255

Osteoarthritis	Myocardial infarction	1.36	0.95	1.94	0.0890	21148	253
Osteoarthritis	Anemia	1.35	0.78	2.34	0.2802	21193	101
Infections and excema	Cerebral infarction	1.35	0.43	4.23	0.6048	21183	225
Asthma	Infections and excema	1.35	0.85	2.15	0.2079	21090	184
Sleep disorders	Deep vein thrombosis	1.35	0.68	2.67	0.3928	21142	166
Asthma	Osteoarthritis	1.35	1.16	1.56	0.0001	20383	1837
Osteoarthritis	Angina pectoris	1.34	0.98	1.84	0.0671	20996	315
Deep vein thrombosis	Pancreatitis	1.34	0.19	9.62	0.7736	21176	87
Diabetes	Kidney cancer	1.33	0.62	2.87	0.4637	21225	41
Infections and excema	Diabetes	1.33	0.97	1.82	0.0780	20434	3255
Arrhythmias	Diabetes	1.33	1.14	1.55	0.0004	20434	3255
Arrhythmias	Asthma	1.32	0.94	1.86	0.1039	19851	929
Heart failure	Hypertension	1.31	0.78	2.23	0.3111	18018	1988
Osteoarthritis	Sleep disorders	1.31	1.11	1.55	0.0014	20845	1271
Diseases of liver	Sleep disorders	1.30	0.70	2.43	0.4075	20845	1271
Back pain	Myocardial infarction	1.30	0.48	3.48	0.6078	21148	253
Diabetes	Cerebral infarction	1.29	0.93	1.79	0.1310	21183	225
Sleep disorders	Pancreatitis	1.28	0.55	2.98	0.5679	21176	87
Diabetes	Back pain	1.27	0.83	1.95	0.2662	21090	197
Angina pectoris	Hypertension	1.27	0.93	1.75	0.1377	18018	1988
Kidney cancer	Heart failure	1.27	0.18	9.08	0.8113	21187	254
Osteoarthritis	Asthma	1.27	1.02	1.57	0.0293	19851	929
Kidney cancer	Bacterial infections	1.26	0.31	5.06	0.7439	20838	852
Osteoarthritis	Heart failure	1.26	0.90	1.76	0.1849	21187	254
Infections and excema	Pulmonary embolism	1.25	0.31	5.04	0.7553	21179	166
Bacterial infections	Diabetes	1.25	1.06	1.47	0.0089	20434	3255
Hypertension	Asthma	1.24	1.06	1.45	0.0061	19851	929
Asthma	Arrhythmias	1.24	1.02	1.50	0.0337	20923	1068
Myocardial infarction	Hypertension	1.23	0.79	1.92	0.3544	18018	1988
Hypertension	Osteoarthritis	1.23	1.11	1.36	0.0001	20383	1837
Kidney cancer	Diabetes	1.22	0.61	2.45	0.5712	20434	3255
Hypertension	Pancreatitis	1.22	0.75	1.99	0.4224	21176	87
Arrhythmias	Osteoarthritis	1.22	0.97	1.52	0.0852	20383	1837
Myocardial infarction	Sleep disorders	1.21	0.77	1.91	0.4122	20845	1271
Infections and excema	Arrhythmias	1.21	0.70	2.08	0.5028	20923	1068
Pulmonary embolism	Osteoarthritis	1.20	0.71	2.04	0.4906	20383	1837
Hypertension	Back pain	1.20	0.85	1.68	0.2947	21090	197
Pancreatitis	Back pain	1.20	0.17	8.53	0.8592	21090	197
Gout	Sleep disorders	1.20	0.64	2.23	0.5756	20845	1271
Pancreatitis	Infections and excema	1.19	0.17	8.50	0.8627	21090	184
Pulmonary embolism	Asthma	1.18	0.53	2.64	0.6818	19851	929
Asthma	Diabetes	1.18	1.05	1.33	0.0055	20434	3255
Asthma	Angina pectoris	1.17	0.80	1.71	0.4287	20996	315
Osteoarthritis	Diseases of liver	1.17	0.79	1.73	0.4377	21176	236
Pancreatitis	Pulmonary embolism	1.17	0.16	8.35	0.8774	21179	166
Asthma	Diseases of liver	1.15	0.75	1.77	0.5275	21176	236
Renal failure	Osteoarthritis	1.15	0.48	2.77	0.7567	20383	1837
Sleep disorders	Osteoarthritis	1.14	0.93	1.41	0.2136	20383	1837
Sleep disorders	Kidney cancer	1.14	0.35	3.77	0.8281	21225	41
Osteoarthritis	Pancreatitis	1.14	0.56	2.30	0.7238	21176	87
Diseases of liver	Hypertension	1.13	0.62	2.04	0.6888	18018	1988
Infections and excema	Osteoarthritis	1.13	0.72	1.77	0.6105	20383	1837
Osteoarthritis	Bacterial infections	1.12	0.91	1.39	0.2899	20838	852
Osteoarthritis	Kidney cancer	1.12	0.46	2.70	0.8013	21225	41
Asthma	Hypertension	1.11	0.95	1.31	0.1990	18018	1988
Infections and excema	Pancreatitis	1.11	0.15	7.97	0.9194	21176	87
Osteoarthritis	Arrhythmias	1.10	0.93	1.31	0.2796	20923	1068
Renal failure	Diabetes	1.10	0.52	2.31	0.8052	20434	3255
Osteoarthritis	Hypertension	1.10	0.93	1.29	0.2598	18018	1988
Kidney cancer	Sleep disorders	1.09	0.35	3.40	0.8767	20845	1271
Myocardial infarction	Asthma	1.09	0.54	2.20	0.8065	19851	929
Diabetes	Arrhythmias	1.09	0.93	1.27	0.3095	20923	1068
Anemia	Osteoarthritis	1.08	0.54	2.16	0.8273	20383	1837
Pulmonary embolism	Myocardial infarction	1.08	0.27	4.34	0.9165	21148	253
Diseases of liver	Arrhythmias	1.08	0.56	2.08	0.8271	20923	1068
Osteoarthritis	Pulmonary embolism	1.08	0.67	1.72	0.7585	21179	166
Arrhythmias	Pulmonary embolism	1.07	0.52	2.20	0.8481	21179	166
Hypertension	Pulmonary embolism	1.06	0.75	1.50	0.7285	21179	166
Angina pectoris	Diseases of liver	1.06	0.50	2.28	0.8745	21176	236
Sleep disorders	Myocardial infarction	1.06	0.66	1.71	0.8037	21148	253
Diseases of liver	Gout	1.06	0.15	7.63	0.9534	21183	109
Osteoarthritis	Diabetes	1.06	0.95	1.18	0.2906	20434	3255
Diabetes	Asthma	1.05	0.85	1.29	0.6671	19851	929
Infections and excema	Angina pectoris	1.05	0.34	3.27	0.9372	20996	315
Bacterial infections	Hypertension	1.04	0.81	1.34	0.7624	18018	1988
Diabetes	Pancreatitis	1.04	0.55	1.95	0.9164	21176	87
Anemia	Myocardial infarction	1.03	0.15	7.38	0.9741	21148	253
Pancreatitis	Sleep disorders	1.03	0.49	2.17	0.9332	20845	1271
Angina pectoris	Asthma	1.02	0.62	1.69	0.9278	19851	929
Diseases of liver	Deep vein thrombosis	1.01	0.14	7.26	0.9891	21142	166
Diabetes	Osteoarthritis	1.01	0.88	1.16	0.9236	20383	1837
Back pain	Pulmonary embolism	1.01	0.25	4.07	0.9917	21179	166
Hypertension	Deep vein thrombosis	0.99	0.69	1.43	0.9694	21142	166
Pancreatitis	Arrhythmias	0.99	0.44	2.21	0.9840	20923	1068
Bacterial infections	Osteoarthritis	0.99	0.78	1.26	0.9263	20383	1837
Osteoarthritis	Cerebral infarction	0.99	0.66	1.47	0.9467	21183	225
Diseases of liver	Myocardial infarction	0.99	0.25	3.97	0.9845	21148	253

Gout	Back pain	0.96	0.13	6.92	0.9682	21090	197
Asthma	Pulmonary embolism	0.96	0.55	1.66	0.8820	21179	166
Back pain	Diabetes	0.96	0.69	1.33	0.7962	20434	3255
Infections and excema	Hypertension	0.96	0.59	1.54	0.8545	18018	1988
Diabetes	Pulmonary embolism	0.94	0.60	1.47	0.7831	21179	166
Cerebral infarction	Asthma	0.93	0.38	2.24	0.8670	19851	929
Diseases of liver	Back pain	0.92	0.13	6.61	0.9376	21090	197
Myocardial infarction	Pulmonary embolism	0.92	0.23	3.75	0.9114	21179	166
Asthma	Myocardial infarction	0.92	0.57	1.49	0.7362	21148	253
Deep vein thrombosis	Hypertension	0.92	0.55	1.53	0.7414	18018	1988
Deep vein thrombosis	Gout	0.89	0.12	6.41	0.9105	21183	109
Myocardial infarction	Pancreatitis	0.89	0.12	6.45	0.9058	21176	87
Gout	Infections and excema	0.89	0.12	6.37	0.9044	21090	184
Anemia	Diabetes	0.88	0.49	1.59	0.6748	20434	3255
Back pain	Sleep disorders	0.88	0.51	1.52	0.6442	20845	1271
Deep vein thrombosis	Asthma	0.86	0.41	1.80	0.6846	19851	929
Deep vein thrombosis	Diabetes	0.86	0.59	1.24	0.4055	20434	3255
Asthma	Anemia	0.84	0.41	1.73	0.6330	21193	101
Pulmonary embolism	Hypertension	0.84	0.43	1.61	0.5890	18018	1988
Back pain	Hypertension	0.83	0.53	1.30	0.4074	18018	1988
Angina pectoris	Pulmonary embolism	0.82	0.30	2.24	0.7011	21179	166
Anemia	Hypertension	0.81	0.34	1.95	0.6351	18018	1988
Angina pectoris	Sleep disorders	0.80	0.54	1.18	0.2550	20845	1271
Heart failure	Osteoarthritis	0.80	0.45	1.41	0.4324	20383	1837
Diseases of liver	Osteoarthritis	0.79	0.41	1.53	0.4908	20383	1837
Cerebral infarction	Osteoarthritis	0.75	0.41	1.36	0.3404	20383	1837
Kidney cancer	Hypertension	0.75	0.19	2.98	0.6773	18018	1988
Angina pectoris	Anemia	0.74	0.18	3.02	0.6714	21193	101
Diabetes	Deep vein thrombosis	0.73	0.42	1.26	0.2609	21142	166
Pancreatitis	Asthma	0.73	0.23	2.26	0.5823	19851	929
Back pain	Gout	0.73	0.10	5.22	0.7521	21183	109
Angina pectoris	Osteoarthritis	0.73	0.52	1.02	0.0620	20383	1837
Kidney cancer	Arrhythmias	0.72	0.18	2.88	0.6400	20923	1068
Asthma	Pancreatitis	0.71	0.29	1.75	0.4577	21176	87
Arrhythmias	Deep vein thrombosis	0.69	0.25	1.87	0.4632	21142	166
Pancreatitis	Myocardial infarction	0.67	0.09	4.80	0.6922	21148	253
Pancreatitis	Heart failure	0.66	0.09	4.73	0.6813	21187	254
Infections and excema	Asthma	0.66	0.30	1.48	0.3135	19851	929
Anemia	Sleep disorders	0.66	0.21	2.05	0.4722	20845	1271
Myocardial infarction	Osteoarthritis	0.66	0.39	1.11	0.1183	20383	1837
Cerebral infarction	Pulmonary embolism	0.65	0.09	4.68	0.6708	21179	166
Deep vein thrombosis	Infections and excema	0.64	0.09	4.55	0.6530	21090	184
Pulmonary embolism	Diseases of liver	0.62	0.09	4.45	0.6378	21176	236
Deep vein thrombosis	Angina pectoris	0.61	0.15	2.45	0.4857	20996	315
Pancreatitis	Hypertension	0.60	0.25	1.44	0.2525	18018	1988
Asthma	Kidney cancer	0.57	0.14	2.36	0.4379	21225	41
Angina pectoris	Deep vein thrombosis	0.52	0.13	2.11	0.3592	21142	166
Pulmonary embolism	Angina pectoris	0.43	0.06	3.04	0.3951	20996	315
Arrhythmias	Kidney cancer	0.39	0.05	2.89	0.3593	21225	41
Kidney cancer	Osteoarthritis	0.29	0.04	2.09	0.2216	20383	1837

Supplement table 15 shows that among the first four diseases in obese participants, there were 140 different disease combinations, each of low prevalence (6.2% or less, N<12). The obesity-related diseases that occurred most frequently were diabetes (75.4%, N=147), hypertension (671.8%, N=140), sleep disorders (42.6%, N=83), osteoarthritis (42.1%, N=82), arrhythmias (34.4%, N=67), bacterial infections (31.3%, N=61) and asthma (22.1%, N=43) (supplement table 16).

Supplementary table 15. Frequency of disease combinations in complex multimorbidity (the first 4 diseases irrespective of temporal order), N of participants with obesity and complex multimorbidity = 195

Disease combination in multimorbidity	N	%
diabetes-sleep disorders-hypertension-osteoarthritis	12	6.2
diabetes-sleep disorders-hypertension-bacterial infections	6	3.1
diabetes-hypertension-osteoarthritis-asthma	6	3.1
diabetes-hypertension-osteoarthritis-arrhythmias	6	3.1
diabetes-arrhythmias-myocardial infarction-angina pectoris	<5	2.1 or less
diabetes-asthma-diseases of liver-arrhythmias	<5	2.1 or less
diabetes-bacterial infections-arrhythmias-angina pectoris	<5	2.1 or less
diabetes-bacterial infections-diseases of liver-anaemia	<5	2.1 or less
diabetes-bacterial infections-myocardial infarction-angina pectoris	<5	2.1 or less
diabetes-gout-hypertension-asthma	<5	2.1 or less
diabetes-heart failure-bacterial infections-angina pectoris	<5	2.1 or less
diabetes-heart failure-hypertension-arrhythmias	<5	2.1 or less
diabetes-heart failure-hypertension-asthma	<5	2.1 or less
diabetes-heart failure-hypertension-cerebral infarction	<5	2.1 or less
diabetes-heart failure-hypertension-osteoarthritis	<5	2.1 or less
diabetes-heart failure-osteoarthritis-angina pectoris	<5	2.1 or less
diabetes-heart failure-osteoarthritis-arrhythmias	<5	2.1 or less
diabetes-heart failure-osteoarthritis-asthma	<5	2.1 or less
diabetes-heart failure-osteoarthritis-back pain	<5	2.1 or less
diabetes-heart failure-osteoarthritis-bacterial infections	<5	2.1 or less
diabetes-heart failure-pulmonary embolism-arrhythmias	<5	2.1 or less
diabetes-heart failure-skin infections and excema-bacterial infections	<5	2.1 or less
diabetes-hypertension-arrhythmias-cerebral infarction	<5	2.1 or less
diabetes-hypertension-arrhythmias-myocardial infarction	<5	2.1 or less
diabetes-hypertension-asthma-anaemia	<5	2.1 or less
diabetes-hypertension-asthma-angina pectoris	<5	2.1 or less
diabetes-hypertension-asthma-pancreatitis	<5	2.1 or less
diabetes-hypertension-back pain-angina pectoris	<5	2.1 or less
diabetes-hypertension-back pain-cerebral infarction	<5	2.1 or less
diabetes-hypertension-bacterial infections-anaemia	<5	2.1 or less
diabetes-hypertension-bacterial infections-angina pectoris	<5	2.1 or less
diabetes-hypertension-bacterial infections-arrhythmias	<5	2.1 or less
diabetes-hypertension-bacterial infections-asthma	<5	2.1 or less
diabetes-hypertension-deep vein thrombosis-cerebral infarction	<5	2.1 or less
diabetes-hypertension-diseases of liver-anaemia	<5	2.1 or less
diabetes-hypertension-diseases of liver-angina pectoris	<5	2.1 or less
diabetes-hypertension-diseases of liver-arrhythmias	<5	2.1 or less
diabetes-hypertension-myocardial infarction-angina pectoris	<5	2.1 or less
diabetes-hypertension-osteoarthritis-angina pectoris	<5	2.1 or less
diabetes-hypertension-osteoarthritis-back pain	<5	2.1 or less
diabetes-hypertension-osteoarthritis-bacterial infections	<5	2.1 or less
diabetes-hypertension-osteoarthritis-cerebral infarction	<5	2.1 or less
diabetes-hypertension-osteoarthritis-diseases of liver	<5	2.1 or less
diabetes-hypertension-osteoarthritis-myocardial infarction	<5	2.1 or less
diabetes-hypertension-osteoarthritis-skin infections and excema	<5	2.1 or less
diabetes-hypertension-pancreatitis-arrhythmias	<5	2.1 or less
diabetes-hypertension-pancreatitis-back pain	<5	2.1 or less
diabetes-hypertension-pulmonary embolism-angina pectoris	<5	2.1 or less
diabetes-hypertension-renal failure-diseases of liver	<5	2.1 or less
diabetes-hypertension-skin infections and excema-bacterial infections	<5	2.1 or less
diabetes-osteoarthritis-back pain-arrhythmias	<5	2.1 or less

diabetes-osteoarthritis-bacterial infections-myocardial infarction	<5	2.1 or less
diabetes-osteoarthritis-myocardial infarction-angina pectoris	<5	2.1 or less
diabetes-osteoarthritis-pulmonary embolism-angina pectoris	<5	2.1 or less
diabetes-pulmonary embolism-arrhythmias-kidney cancer	<5	2.1 or less
diabetes-pulmonary embolism-bacterial infections-arrhythmias	<5	2.1 or less
diabetes-pulmonary embolism-bacterial infections-diseases of liver	<5	2.1 or less
diabetes-renal failure-bacterial infections-anaemia	<5	2.1 or less
diabetes-renal failure-bacterial infections-asthma	<5	2.1 or less
diabetes-renal failure-skin infections and excema-diseases of liver	<5	2.1 or less
diabetes-skin infections and excema-myocardial infarction-angina pectoris	<5	2.1 or less
diabetes-sleep disorders-arrhythmias-myocardial infarction	<5	2.1 or less
diabetes-sleep disorders-asthma-arrhythmias	<5	2.1 or less
diabetes-sleep disorders-asthma-diseases of liver	<5	2.1 or less
diabetes-sleep disorders-bacterial infections-arrhythmias	<5	2.1 or less
diabetes-sleep disorders-bacterial infections-myocardial infarction	<5	2.1 or less
diabetes-sleep disorders-gout-heart failure	<5	2.1 or less
diabetes-sleep disorders-heart failure-arrhythmias	<5	2.1 or less
diabetes-sleep disorders-heart failure-osteoarthritis	<5	2.1 or less
diabetes-sleep disorders-hypertension-anaemia	<5	2.1 or less
diabetes-sleep disorders-hypertension-angina pectoris	<5	2.1 or less
diabetes-sleep disorders-hypertension-arrhythmias	<5	2.1 or less
diabetes-sleep disorders-hypertension-asthma	<5	2.1 or less
diabetes-sleep disorders-hypertension-back pain	<5	2.1 or less
diabetes-sleep disorders-hypertension-cerebral infarction	<5	2.1 or less
diabetes-sleep disorders-hypertension-diseases of liver	<5	2.1 or less
diabetes-sleep disorders-hypertension-kidney cancer	<5	2.1 or less
diabetes-sleep disorders-hypertension-myocardial infarction	<5	2.1 or less
diabetes-sleep disorders-hypertension-pulmonary embolism	<5	2.1 or less
diabetes-sleep disorders-hypertension-skin infections and excema	<5	2.1 or less
diabetes-sleep disorders-osteoarthritis-asthma	<5	2.1 or less
diabetes-sleep disorders-osteoarthritis-bacterial infections	<5	2.1 or less
diabetes-sleep disorders-osteoarthritis-deep vein thrombosis	<5	2.1 or less
diabetes-sleep disorders-osteoarthritis-myocardial infarction	<5	2.1 or less
diabetes-sleep disorders-osteoarthritis-pulmonary embolism	<5	2.1 or less
diabetes-sleep disorders-pulmonary embolism-asthma	<5	2.1 or less
gout-bacterial infections-arrhythmias-anaemia	<5	2.1 or less
gout-heart failure-asthma-arrhythmias	<5	2.1 or less
gout-heart failure-hypertension-myocardial infarction	<5	2.1 or less
gout-heart failure-myocardial infarction-angina pectoris	<5	2.1 or less
gout-hypertension-bacterial infections-angina pectoris	<5	2.1 or less
heart failure-arrhythmias-myocardial infarction-angina pectoris	<5	2.1 or less
heart failure-arrhythmias-myocardial infarction-cerebral infarction	<5	2.1 or less
heart failure-bacterial infections-diseases of liver-back pain	<5	2.1 or less
heart failure-hypertension-bacterial infections-arrhythmias	<5	2.1 or less
heart failure-hypertension-bacterial infections-myocardial infarction	<5	2.1 or less
heart failure-osteoarthritis-arrhythmias-cerebral infarction	<5	2.1 or less
heart failure-pulmonary embolism-myocardial infarction-cerebral infarction	<5	2.1 or less
hypertension-arrhythmias-angina pectoris-cerebral infarction	<5	2.1 or less
hypertension-asthma-back pain-arrhythmias	<5	2.1 or less
hypertension-bacterial infections-asthma-pancreatitis	<5	2.1 or less
hypertension-bacterial infections-diseases of liver-arrhythmias	<5	2.1 or less
hypertension-osteoarthritis-asthma-arrhythmias	<5	2.1 or less

hypertension-osteoarthritis-asthma-back pain	<5	2.1 or less
hypertension-osteoarthritis-asthma-myocardial infarction	<5	2.1 or less
hypertension-osteoarthritis-bacterial infections-back pain	<5	2.1 or less
hypertension-osteoarthritis-bacterial infections-diseases of liver	<5	2.1 or less
hypertension-osteoarthritis-bacterial infections-pancreatitis	<5	2.1 or less
hypertension-osteoarthritis-cerebral infarction-kidney cancer	<5	2.1 or less
hypertension-osteoarthritis-deep vein thrombosis-asthma	<5	2.1 or less
hypertension-renal failure-arrhythmias-angina pectoris	<5	2.1 or less
hypertension-renal failure-osteoarthritis-asthma	<5	2.1 or less
hypertension-skin infections and excema-bacterial infections-pancreatitis	<5	2.1 or less
hypertension-skin infections and excema-pancreatitis-back pain	<5	2.1 or less
osteoarthritis-arrhythmias-myocardial infarction-angina pectoris	<5	2.1 or less
osteoarthritis-pulmonary embolism-deep vein thrombosis-back pain	<5	2.1 or less
renal failure-skin infections and excema-bacterial infections-arrhythmias	<5	2.1 or less
skin infections and excema-bacterial infections-diseases of liver-arrhythmias	<5	2.1 or less
skin infections and excema-bacterial infections-myocardial infarction-angina pectoris	<5	2.1 or less
sleep disorders-asthma-arrhythmias-myocardial infarction	<5	2.1 or less
sleep disorders-bacterial infections-asthma-arrhythmias	<5	2.1 or less
sleep disorders-bacterial infections-back pain-arrhythmias	<5	2.1 or less
sleep disorders-gout-heart failure-pulmonary embolism	<5	2.1 or less
sleep disorders-heart failure-asthma-arrhythmias	<5	2.1 or less
sleep disorders-heart failure-hypertension-bacterial infections	<5	2.1 or less
sleep disorders-heart failure-osteoarthritis-arrhythmias	<5	2.1 or less
sleep disorders-hypertension-arrhythmias-angina pectoris	<5	2.1 or less
sleep disorders-hypertension-arrhythmias-cerebral infarction	<5	2.1 or less
sleep disorders-hypertension-asthma-angina pectoris	<5	2.1 or less
sleep disorders-hypertension-bacterial infections-asthma	<5	2.1 or less
sleep disorders-hypertension-osteoarthritis-arrhythmias	<5	2.1 or less
sleep disorders-hypertension-osteoarthritis-bacterial infections	<5	2.1 or less
sleep disorders-hypertension-osteoarthritis-cerebral infarction	<5	2.1 or less
sleep disorders-hypertension-osteoarthritis-deep vein thrombosis	<5	2.1 or less
sleep disorders-hypertension-osteoarthritis-myocardial infarction	<5	2.1 or less
sleep disorders-hypertension-osteoarthritis-skin infections and excema	<5	2.1 or less
sleep disorders-osteoarthritis-asthma-angina pectoris	<5	2.1 or less
sleep disorders-osteoarthritis-asthma-arrhythmias	<5	2.1 or less
sleep disorders-osteoarthritis-bacterial infections-cerebral infarction	<5	2.1 or less
sleep disorders-osteoarthritis-deep vein thrombosis-asthma	<5	2.1 or less
sleep disorders-skin infections and excema-asthma-arrhythmias	<5	2.1 or less

Supplementary table 16. Proportions of obesity-related diseases in obese participants by number of obesity-related diseases

Health condition	Proportion (%) of disease<br>Number of diseases<br>0 (N=9195)	1 (N=3614)	2 (N=1413)	3 (N=450)	4 or more† (N=195)
Diabetes	0.0	23.3	49.0	61.1	75.4
Hypertension	0.0	16.7	44.2	56.0	71.8
Sleep disorders	0.0	9.3	18.5	26.7	42.6
Osteoarthritis	0.0	17.4	24.4	34.0	42.1
Arrhythmias	0.0	6.8	12.9	25.8	34.4
Bacterial infections	0.0	5.1	11.1	17.1	31.3
Asthma	0.0	9.3	14.7	18.9	22.1
Angina pectoris	0.0	0.9	3.9	8.4	20.0
Heart failure	0.0	0.4	2.2	6.4	17.9
Myocardial infarction	0.0	1.2	1.6	7.6	15.9
Diseases of liver	0.0	1.5	2.0	5.1	10.3
Back pain	0.0	1.5	2.3	4.7	9.2
Pulmonary embolism	0.0	1.0	1.9	6.9	8.2
Skin infections and excema	0.0	1.2	2.2	2.9	8.2
Cerebral infarction	0.0	1.1	2.5	4.9	7.7
Renal failure	0.0	0.1	0.4	2.0	6.7
Gout	0.0	0.2	0.8	3.3	5.1
Anaemia	0.0	0.6	1.6	1.6	4.6
Pancreatitis	0.0	0.9	0.8	2.0	3.6
Deep vein thrombosis	0.0	1.3	2.5	4.2	3.1
Kidney cancer	0.0	0.2	0.5	0.4	1.5

*6372 participants with prevalent obesity-related diseases at baseline were excluded from the analysis. Diseases are shown by descending incidence in the 4 or more diseases category.

†Proportions counted for the first 4 diseases only

Sensitivity analyses also show that the associations of BMI with simple and complex multimorbidity were replicable using alternative indicators of multimorbidity, such as count of obesity-related diseases (supplement table 17) and additive hazards for multimorbidity (supplement table 18).

Supplementary table 17. Associations of BMI category with the rate of obesity-related diseases in Finnish cohorts

BMI category	N	Number of obesity-related diseases
**Finnish cohorts**		**Rate per 10,000 person-years (95% CI)**
Normal weight	55857	162 (150-175)
Overweight	24495	246 (227-266)
Obese	14867	445 (411-481)
Obese, class 1	12496	409 (378-443)
Obese, class 2	1839	597 (544-654)
Obese, class 3	532	775 (689-871)
		**Rate ratio (95% CI)***
Normal weight	55857	1.00 (reference)
Overweight	24495	1.52 (1.47-1.56)
Obese	14867	2.74 (2.67-2.82)
Obese, class 1	12496	2.52 (2.45-2.60)
Obese, class 2	1839	3.68 (3.48-3.89)
Obese, class 3	532	4.78 (4.37-5.23)

*Adjusted for age, sex, cohort, education, neighbourhood socioeconomic deprivation.

Supplementary table 18. Difference in hazard of multimorbidity between participants with overweight and obesity compared to normal weight in Finnish cohorts

		Hazard difference per 10,000 person-years (95% CI)*			
BMI category	N	1st disease	2nd disease	3rd disease	4th disease
Normal weight	55857	0 (reference)	0 (reference)	0 (reference)	0 (reference)
Overweight	24495	81 (74-88)	22 (19-25)	6 (4-7)	1 (1-2)
Obese	14867	289 (277-301)	109 (103-115)	35 (32-39)	11 (9-13)
Obese, class 1	12496	260 (247-273)	95 (89-102)	29 (25-32)	8 (7-10)
Obese, class 2	1839	420 (381-459)	161 (141-181)	61 (50-72)	24 (18-31)
Obese, class 3	532	568 (484-652)	248 (202-294)	96 (70-123)	26 (12-39)

*Adjusted for age, sex, ethnicity(UK Biobank) and cohort (Finnish multicohort), education, neighbourhood socioeconomic deprivation.

External replication in UK Biobank

As shown in Supplement figure 3, UK Biobank participants were older than participants in the Finnish cohorts. Supplement table 19 shows that all the 21 associations with obesity-related diseases and, with few exceptions, diseases not robustly associated with obesity in the Finnish cohorts were replicated in UK Biobank.

Supplement figure 3. Age distribution at BMI assessment in the Finnish cohorts and UK Biobank

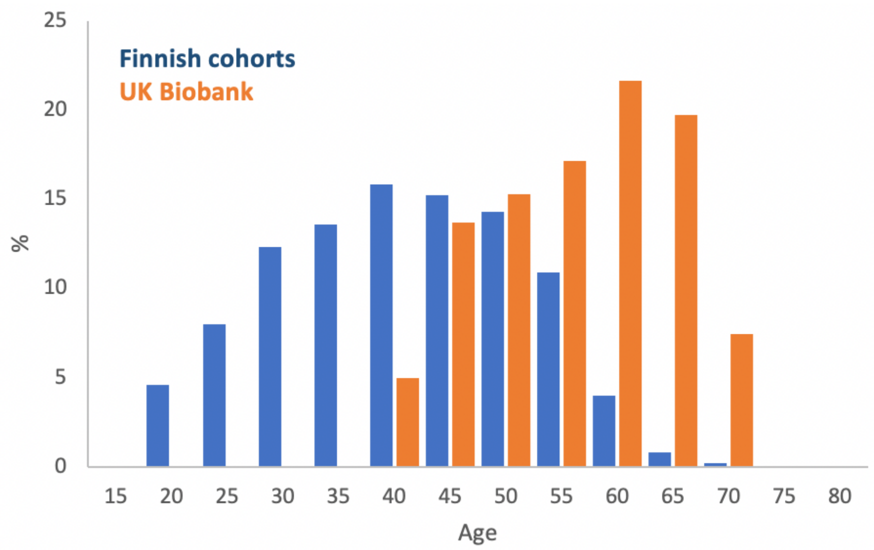

Supplement table 19. Associations of obesity versus normal weight with 78 health outcomes in UK Biobank

Disease outcome	N (total)	I (incident case	Hazard ratio*	Lower CL	Upper CL	P-value
**Infections**	281750	13005	1.37	1.32	1.42	<.0001
Bacterial infections	283294	11133	1.43	1.37	1.48	<.0001
Viral infections	283286	1723	1.20	1.09	1.32	0.0002
**Cancer**	271296	35140	1.06	1.04	1.08	<.0001
Colorectal cancer	283600	3395	1.26	1.18	1.35	<.0001
Lung cancer	284464	2743	0.84	0.78	0.90	<.0001
Melanoma	281311	10484	0.84	0.80	0.87	<.0001
Breast cancer	165822	6144	1.19	1.13	1.25	<.0001
Prostate cancer	113619	4241	0.87	0.81	0.92	<.0001
Kidney cancer	284402	855	2.00	1.73	2.31	<.0001
Brain cancer	284585	521	0.91	0.76	1.08	0.2761
Leukaemia, lymphoma	283757	2616	1.11	1.03	1.20	0.0085
**Diseases of the blood**	280942	11087	1.42	1.36	1.47	<.0001
Anaemia	281897	9521	1.46	1.40	1.52	<.0001
**Endocrine diseases**	282003	3490	2.04	1.90	2.18	<.0001
Diabetes	283700	1240	4.53	3.93	5.23	<.0001
**Mental and behavioural disorders**	282206	3176	0.98	0.91	1.05	0.4911
Dementia	284592	1224	0.76	0.68	0.86	<.0001
Disorders due to substance abuse	284078	606	0.76	0.64	0.89	0.001
Psychotic disorders	284278	238	1.15	0.88	1.49	0.3083
Mood disorders	283660	673	1.16	0.99	1.35	0.0672
Neurotic disorders	284071	727	1.12	0.97	1.30	0.135
**Diseases of the nervous system**	274468	14437	1.62	1.57	1.68	<.0001
Parkinson disease	284585	485	0.76	0.63	0.92	0.0038
Multiple sclerosis	284287	196	1.04	0.78	1.40	0.7751
Epilepsy	284164	596	0.90	0.76	1.06	0.2088
Headaches	283923	1346	1.31	1.17	1.46	<.0001
TIA	283983	1747	1.30	1.18	1.43	<.0001
Sleep disorders	282994	1567	4.10	3.63	4.62	<.0001
**Diseases of the eye**	273560	31290	1.18	1.15	1.21	<.0001
**Diseases of the ear**	282093	2979	1.23	1.14	1.32	<.0001
**Diseases of the circulatory system**	256747	36359	1.51	1.48	1.55	<.0001
Hypertension	284056	1364	1.98	1.77	2.21	<.0001
Ischemic heart diseases	275595	13732	1.75	1.69	1.82	<.0001
Angina pectoris	280891	3644	2.00	1.87	2.15	<.0001
Myocardial infarction	282033	5387	1.62	1.53	1.71	<.0001
Pulmonary embolism	283854	2661	2.34	2.15	2.54	<.0001
Arrhythmias	281465	8184	1.61	1.54	1.68	<.0001
Heart failure	284224	2601	3.24	2.96	3.55	<.0001
Cerebrovascular diseases	282924	5892	1.26	1.20	1.33	<.0001
Stroke	283250	5144	1.27	1.20	1.34	<.0001
Intracerebral haemorrhage	284484	754	0.98	0.84	1.13	0.7415
Cerebral infarction	283989	3678	1.43	1.34	1.53	<.0001
Arteriosclerosis	284411	650	1.23	1.05	1.44	0.0104
Deep vein thrombosis	283375	2152	2.07	1.89	2.26	<.0001
**Diseases of the respiratory system**	273181	20302	1.30	1.27	1.34	<.0001
Influenza and Pneumonia	282894	9234	1.29	1.24	1.34	<.0001
Chronic obstructive bronchitis	283865	3141	0.88	0.82	0.95	0.0006
Asthma	283464	1244	2.23	1.98	2.51	<.0001
**Diseases of the digestive system**	229094	64087	1.22	1.20	1.24	<.0001
Appendicitis	283424	1282	1.10	0.98	1.23	0.1102
Inflammatory bowel disease	278989	6083	1.12	1.06	1.18	<.0001
Diseases of liver	283965	1637	2.22	2.00	2.46	<.0001
Alcoholic liver disease	284470	407	1.45	1.18	1.77	0.0003
Pancreatitis	284030	1185	2.23	1.97	2.53	<.0001
**Diseases of the skin**	270951	16464	1.37	1.33	1.41	<.0001
Infections and excema	280627	6085	2.31	2.19	2.44	<.0001
**Diseases of the musculoskeletal system**	250127	46585	1.65	1.62	1.68	<.0001
Rheumatoid arthritis and related disorders	283023	2684	1.49	1.38	1.61	<.0001
Gout	284512	398	5.27	4.00	6.95	<.0001
Osteoarthritis	275947	22168	2.56	2.49	2.64	<.0001
Sciatica	282310	4131	1.71	1.60	1.82	<.0001
Back pain	280379	6012	1.83	1.73	1.93	<.0001
Soft tissue disorders	273998	16957	1.50	1.45	1.54	<.0001
**Diseases of the genitourinary system**	243199	29919	1.39	1.36	1.43	<.0001
Renal failure	284198	2658	2.32	2.13	2.52	<.0001
**Pregnancy complications**	165450	167	0.83	0.58	1.18	0.3022
Spontaneous abortion	168799	58	0.69	0.37	1.28	0.2411
Hypertension in pregnancy	169282	18	1.66	0.62	4.43	0.3098
Diabetes in pregnancy	169858	6	3.01	0.58	15.55	0.1879
**Miscellaneous**						
Circulatory and respiratory symptoms	268495	21015	1.54	1.50	1.58	<.0001
Digestive and abdominal symptoms	267986	24181	1.10	1.08	1.13	<.0001
Injury	270739	22318	0.99	0.96	1.01	0.2838
Poisoning	282913	1438	1.34	1.21	1.49	<.0001
Road accidents			–	–	–	–
Falls			–	–	–	–
Self-harm			–	–	–	–
**Death**	284633	20032	1.25	1.22	1.29	<.0001

*Adjusted for age, sex, ethnicity, education and neighbourhood deprivation.

The associations of BMI with simple and complex multimorbidity and the dose-response association across severity levels of obesity in UK Biobank were robust to additional adjustment for lifestyle factors (supplement table 19). Supplement table 20 provides a summary of consistencies and inconsistencies in the results from the Finnish cohorts and UK Biobank.

Supplement table 20. Lifestyle-adjusted associations of BMI category with incident obesity-related disease and multimorbidity in UK Biobank

		1st disease			2nd disease			3rd disease			4th disease		
**BMI category**	**N (total)**	**N (cases)**	**HR (95% CI)***	**PAF (95% CI)**	**N (cases)**	**HR (95% CI)***	**PAF (95% CI)**	**N (cases)**	**HR (95% CI)***	**PAF (95% CI)**	**N (cases)**	**HR (95% CI)***	**PAF (95% CI)**
**UK Biobank**													
Underweight	2387	423	1.06 (0.96-1.17)	0.02 (-0.02-0.07)	74	1.05 (0.83-1.32)	0.02 (-0.06-0.11)	20	1.38 (0.89-2.16)	0.13 (-0.04-0.36)	5	–	–
Normal weight	150423	25787	1.00 (reference)	0.00 (reference)	4620	1.00 (reference)	0.00 (reference)	949	1.00 (reference)	0.00 (reference)	203	1.00 (reference)	0.00 (reference)
Overweight	188761	43734	1.28 (1.26-1.30)	9.0 (8.5-9.5)	9149	1.37 (1.32-1.42)	10.5 (9.3-11.6)	2032	1.40 (1.30-1.52)	10.5 (8.2-12.6)	482	1.50 (1.27-1.77)	11.3 (7-15.2)
Obese	100510	31931	1.88 (1.85-1.91)	15.2 (14.8-15.5)	8638	2.47 (2.38-2.56)	22.4 (21.7-23.1)	2390	3.08 (2.85-3.32)	28.7 (27.2-30.1)	691	3.91 (3.33-4.58)	35.2 (32.2-37.7)
Obese, class 1	73494	21912	1.71 (1.68-1.74)	8.9 (8.7-9.2)	5569	2.12 (2.04-2.21)	12.4 (11.9-12.9)	1433	2.46 (2.26-2.67)	14.6 (13.5-15.5)	388	2.93 (2.47-3.48)	16.8 (14.8-18.5)
Obese, class 2	19767	7048	2.24 (2.18-2.30)	4.2 (4-4.3)	2051	3.14 (2.98-3.31)	6.4 (6.1-6.7)	623	4.31 (3.89-4.77)	8.9 (8.3-9.4)	198	6.02 (4.93-7.34)	11.8 (10.7-12.7)
Obese, class 3	7249	2971	2.90 (2.79-3.01)	2.4 (2.2-2.5)	1018	4.89 (4.57-5.24)	4.3 (4-4.5)	334	7.32 (6.45-8.32)	6.2 (5.8-6.7)	105	10.15 (7.98-12.91)	7.9 (7-8.8)

*Adjusted for age, sex, ethnicity, education, neighbourhood socioeconomic deprivation, smoking, alcohol consumption and physical activity.

Supplement table 21. Comparison of findings between Finnish cohorts and UK Biobank

Health outcome or Analysis	Finnish cohorts	UK Biobank
**Multimorbidity components**		
Diabetes	Robust association	Robust association replicated
Skin infections and eczema	Robust association	Robust association replicated
Bacterial infections	Robust association	Association replicated, HR=1.43, p<0.0001
Sleep disorders	Robust association	Robust association replicated
Anaemia	Robust association	Association replicated, HR=1.46, p<0.0001
Pancreatitis	Robust association	Robust association replicated
Diseases of liver	Robust association	Robust association replicated
Heart failure	Robust association	Robust association replicated
Hypertension	Robust association	Robust association replicated
Pulmonary embolism	Robust association	Robust association replicated
Deep vein thrombosis	Robust association	Robust association replicated
Arrhythmias	Robust association	Robust association replicated
Myocardial infarction	Robust association	Robust association replicated
Angina pectoris	Robust association	Robust association replicated
Cerebral infarction	Robust association	Association replicated, HR=1.43, p<0.0001
Gout	Robust association	Robust association replicated
Osteoarthritis	Robust association	Robust association replicated
Back pain	Robust association	Robust association replicated
Asthma	Robust association	Robust association replicated
Renal failure	Robust association	Robust association replicated
Kidney cancer	Association HR=1.57, p=0.03	Robust association replicated
**Multimorbidity analysis**		
One obesity-related disease	Strong association	Strong association
Two obesity-related diseases (simple multimorbidity)	Stronger association	Stronger association
Three obesity-related diseases	Even stronger association	Even stronger association
Four obesity-related diseases (complex multimorbidity)	The strongest association	The strongest association
**Relation with severity of obesity**		
One obesity-related disease	Dose-response	Dose-response
Two obesity-related diseases (simple multimorbidity)	Dose-response	Dose-response
Three obesity-related diseases	Dose-response	Dose-response
Four obesity-related diseases (complex multimorbidity)	Dose-response	Dose-response
**Death analysis**	Moderate association	Moderate association
**Diseases not included in obesity-related multimorbidity definition**		
Diabetes in pregnancy	Robust association, but overlapping condition	No robust association
Hypertension in pregnancy	Robust association, but overlapping condition	No robust association
Viral infections	No robust association	No robust association
TIA	No robust association	No robust association
Epilepsy	No robust association	No robust association
Multiple sclerosis	No robust association	No robust association
Headaches	No robust association	No robust association
Parkinson disease	No robust association	No robust association
Alcoholic liver disease	No robust association	No robust association
Inflammatory bowel disease	No robust association	No robust association
Appendicitis	No robust association	No robust association
Intracerebral haemorrhage	No robust association	No robust association
Stroke	No robust association	No robust association
Ischemic heart diseases	No robust association	Robust association, but overlap with MI & angina
Cerebrovascular diseases	No robust association	No robust association
Arteriosclerosis	No robust association	No robust association
Rheumatoid arthritis and related disorders	No robust association	No robust association
Soft tissue disorders	No robust association	No robust association
Sciatica	No robust association	Robust association
Influenza and pneumonia	No robust association	No robust association
Chronic obstructive bronchitis	No robust association	No robust association
Spontaneous abortion	No robust association	No robust association
Digestive and abdominal symptoms	No robust association	No robust association
Circulatory and respiratory symptoms	No robust association	Robust association
Poisoning	No robust association	No robust association
Self-harm	No robust association	No data
Falls	No robust association	No data
Violence	No robust association	No data
Injury	No robust association	No robust association
Road accidents	No robust association	No data
Kidney cancer	No robust association	No robust association
Leukaemia, lymphoma	No robust association	No robust association
Colorectal cancer	No robust association	No robust association
Melanoma	No robust association	No robust association
Prostate cancer	No robust association	No robust association
Breast cancer	No robust association	No robust association
Brain cancer	No robust association	No robust association
Lung cancer	No robust association	No robust association
Mood disorders	No robust association	No robust association
Neurotic disorders	No robust association	No robust association
Psychotic disorders	No robust association	No robust association
Disorders due to substance abuse	No robust association	No robust association
Dementia	No robust association	No robust association

*Robust association refers to HR for obesity vs normal weight > 1.5 and $P < 6.3 \times 10^{-4}$

Discussion

Mendelian randomization, an approach to evaluate causality, uses genetic variants that serve as a proxy for modifiable risk factors, such as obesity. This approach avoids some of the key limitations of observational studies, since allocation of genetic variants is random with regard to potential confounders, and genotype is not modified by disease (abolishing reverse causality). Except for anaemia and pancreatitis, Mendelian randomization studies support a causal association between BMI and the 21 obesity-related diseases included in our analysis of obesity-related complex multimorbidity (supplement table 21).[21-30] We found no large-scale Mendelian randomization studies on obesity, anaemia and pancreatitis.

Supplement table 22. Mendelian randomisation evidence on causality for observed obesity-related diseases

Obesity-related disease in the current study	Evidence from Mendelian randomisation studies	Effect estimate	Reference
Diabetes	Causal association with type 2 diabetes supported	HR per SD 2.03 (95% CI 1.88–2.19)	Larsson et al (2021)
Skin infections and eczema	Causal association with skin infections supported	HR 1.12 (95% CI 1.03–1.22)	Winter-Jenssen et al (2020)
Bacterial infections	Causal association with bloodstream infections supported	HR 1.78 (95% CI: 1.40–2.27)	Rogne et al (2020)
Sleep disorders	Causal association with snoring supported	b per unit = 0.01025 (SE 0.001652) p = 5.27×10^{-10}	Campos et al (2020)
Anaemia	–	–	
Pancreatitis	–	–	
Diseases of liver	Causal association with NALFD supported	HR per SD 1.81 (95% CI 1.22–2.69)	Larsson et al (2021)
Heart failure	Causal association supported	HR per SD 1.69 (95% CI 1.57–1.82)	Larsson et al (2021)
Hypertension	Causal association supported	HR per SD 1.68 (95% CI 1.59–1.78)	Larsson et al (2021)
Pulmonary embolism	Causal association supported	HR per SD 1.34 (95% CI 1.18–1.52)	Larsson et al (2021)
Deep vein thrombosis	Causal association supported	HR per SD 1.59 (95% CI 1.43–1.77)	Larsson et al (2021)
Arrhythmias	Causal association supported	HR per SD 1.62 (95% CI 1.50–1.75)	Larsson et al (2021)
Myocardial infarction	Causal association with coronary artery disease supported	HR per SD 1.49 (95% CI 1.39–1.60)	Gill et al (2021)
	Causal association of body fat mass with myocardial infarction supported	HR per SD 1.40 (95% CI 1.25–1.57)	Si et al (2020)
Angina pectoris	Causal association of visceral adiposity tissue with heart attack/angina	HR per SD 1.78 (95% CI 1.71–1.86)/1.50 (95% CI 1.47–1.54) in women/men	Karlsson et al (2019)
Cerebral infarction	Causal association supported	HR per SD 1.21 (95% CI 1.02–1.44)	Larsson et al (2021)
Gout	Causal association supported	HR per SD 1.92 (95% CI 1.60–2.30)	Larsson et al (2021)
Osteoarthritis	Causal association supported	HR per SD 1.55 (95% CI 1.43–1.69)	Larsson et al (2021)
Back pain	Causal association supported	HR per SD 1.15 (95% CI 1.06–1.25)	Elgaeva et al (2019)
Asthma	Causal association supported	HR per SD 1.36 (95% CI 1.29–1.43)	Larsson at al (2021)
Renal failure	Causal association with chronic kidley disease supported	HR per SD 1.45 (95% CI 1.20–1.75)	Li et sal (2021)
	Causal association with log eGFR supported	b per unit = -0.036 (95%CI -0.032 to -0.027) P = 1×10^{-43}	Kjaergaard et al (2021)
Kidney cancer	Causal association supported	HR per SD 1.49 (95% CI 1.38–1.69)	Larsson et al (2021)

b, beta; CI, confidence interval; eGFR, estimated glomelural filtration rate; HR, hazard ratio; NAFLD, non-alcoholic fatty liver disease; SD, standard deviation; SE, standard error.

Appendix references

1. Korkeila K, Suominen S, Ahvenainen J, et al. Non-response and related factors in a nation-wide health survey. *Eur J Epidemiol* 2001; **17**(11): 991-9.

2. Kivimaki M, Lawlor DA, Davey Smith G, et al. Socioeconomic position, co-occurrence of behavior-related risk factors, and coronary heart disease: the Finnish Public Sector study. *Am J Public Health* 2007; **97**(5): 874-9.

3. Conroy M, Sellors J, Effingham M, et al. The advantages of UK Biobank's open-access strategy for health research. *J Intern Med* 2019; **286**(4): 389-97.

4. WHO. https://www.euro.who.int/en/health-topics/disease-prevention/nutrition/a-healthy-lifestyle/body-mass-index-bmi. (last accessed 15 Dec 2021).

5. Maukonen M, Mannisto S, Tolonen H. A comparison of measured versus self-reported anthropometrics for assessing obesity in adults: a literature review. *Scand J Public Health* 2018; **46**(5): 565-79.

6. Kivimaki M, Batty GD, Pentti J, et al. Association between socioeconomic status and the development of mental and physical health conditions in adulthood: a multi-cohort study. *Lancet Public Health* 2020; **5**(3): e140-e9.

7. Mackenbach JP, Stirbu I, Roskam AJ, et al. Socioeconomic inequalities in health in 22 European countries. *N Engl J Med* 2008; **358**(23): 2468-81.

8. Kivimaki M, Vahtera J, Tabák AG, et al. Neighbourhood socioeconomic disadvantage, risk factors, and diabetes: a cohort study from childhood to middle age. *Lancet Public Health* 2018; **3**(8): e365-e73.

9. Ludwig J, Sanbonmatsu L, Gennetian L, et al. Neighborhoods, obesity, and diabetes--a randomized social experiment. *N Engl J Med* 2011; **365**(16): 1509-19.

10. Townsend P, Phillimore P, Beattie A. Health and Deprivation: Inequality and the North. London: Routledge; 1988.

11. Nyberg ST, Singh-Manoux A, Pentti J, et al. Association of healthy lifestyle with years lived without major chronic diseases. *JAMA Intern Med* 2020; **180**(5): 760-8.

12. Kivimaki M, Singh-Manoux A, Pentti J, et al. Physical inactivity, cardiometabolic disease, and risk of dementia: an individual-participant meta-analysis. *BMJ* 2019; **365**: l1495.

13. Kivimaki M, Batty GD, Pentti J, et al. Modifications to residential neighbourhood characteristics and risk of 79 common health conditions: a prospective cohort study. *Lancet Public Health* 2021; **6**(6): e396-e407.

14. Ervasti J, Pentti J, Nyberg ST, et al. Long working hours and risk of 50 health conditions and mortality outcomes: a multicohort study in four European countries. *Lancet Regional Health – Europe* 2021; 100212.

15. Ioannidis JP. Why most published research findings are false. *PLoS Med* 2005; **2**(8): e124.

16. Ho IS, Azcoaga-Lorenzo A, Akbari A, et al. Examining variation in the measurement of multimorbidity in research: a systematic review of 566 studies. *Lancet Public Health* 2021 [ePub ahead of print].

17. Academy of Medical Sciences. Multimorbidity: a priority for global health research. London: Academy of Medical Sciences, 2018.

18. Aalen O. A model for non-parametric regression analysis of counting processes. In: Klonecki W, Kozek A, eds. Lecture Notes in Statistics, Mathematical Statistics and Probability Theory. New York: Springer-Verlag; 1980.

19. Aalen OO, Scheike TH. Aalen's additive regression model, Encyclopedia of biostatistics. *Wiley Online Library* 2005; **1**.

20. Global BMIMC. Body-mass index and all-cause mortality: individual-participant-data meta-analysis of 239 prospective studies in four continents. *Lancet* 2016; **388**(10046): 776-86.

21. Larsson SC, Burgess S. Causal role of high body mass index in multiple chronic diseases: a systematic review and meta-analysis of Mendelian randomization studies. *BMC Med* 2021; **19**(1): 320.

22. Winter-Jensen M, Afzal S, Jess T, Nordestgaard BG, Allin KH. Body mass index and risk of infections: a Mendelian randomization study of 101,447 individuals. *Eur J Epidemiol* 2020; **35**(4): 347-54.

23. Rogne T, Solligard E, Burgess S, et al. Body mass index and risk of dying from a bloodstream infection: A Mendelian randomization study. *PLoS Med* 2020; **17**(11): e1003413.

24. Campos AI, Garcia-Marin LM, Byrne EM, Martin NG, Cuellar-Partida G, Renteria ME. Insights into the aetiology of snoring from observational and genetic investigations in the UK Biobank. *Nat Commun* 2020; **11**(1): 817.

25. Gill D, Zuber V, Dawson J, et al. Risk factors mediating the effect of body mass index and waist-to-hip ratio on cardiovascular outcomes: Mendelian randomization analysis. *Int J Obes* 2021; **45**(7): 1428-38.

26. Si S, Tewara MA, Li Y, et al. Causal Pathways from Body Components and Regional Fat to Extensive Metabolic Phenotypes: A Mendelian Randomization Study. *Obesity* 2020; **28**(8): 1536-49.

27. Karlsson T, Rask-Andersen M, Pan G, Höglund J, Wadelius C, Ek WE, Johansson Å. Contribution of genetics to visceral adiposity and its relation to cardiovascular and metabolic disease. *Nat Med* 2019; **25**: 1390-95.

28. Elgaeva EE, Tsepilov Y, Freidin MB, Williams FMK, Aulchenko Y, Suri P. Examining causal effects of body mass index on back pain: a Mendelian randomization study. *Eur Spine J* 2020; **29**: 686-91.

29. Li D, Zou Y. Causal effects of life course adiposity on chronic kidney disease: a Mendelian randomization study. *Ann Palliat Med* 2021; **10**(10): 10861-9.

30. Kjaergaard AD, Teumer A, Witte DR, et al. Obesity and Kidney Function: A Two-Sample Mendelian Randomization Study. *Clin Chem* 2021 [ePub ahead of print].
